# Supplementary material for: Costs of community-wide mass drug administration and school-based deworming for soil-transmitted helminths: evidence from a randomised controlled trial in Benin, India and Malawi
Source: BMJ Open. 2022 Jul 8;12(7):e059565. doi: 10.1136/bmjopen-2021-059565 (PMC9272108; doi:10.1136/bmjopen-2021-059565)
Supplement: Supplementary data [file bmjopen-2021-059565supp001.pdf]

APPENDIX

Costs of community-wide mass drug administration and school-based deworming for soil-transmitted helminths: evidence from a randomized-controlled trial in Benin, India, and Malawi

TABLE OF CONTENTS

Appendix 1: Additional details on DeWorm3 activities implemented 2

Appendix 1: Table 1: Implementation characteristics of the DeWorm3 trial at study sites 2

Appendix 1: Table 2: Narrative description of DeWorm3 mass drug administration activities 3

Appendix 2: Additional details on costing methodology 6

Appendix 2: Table 1: DeWorm3 instruments for cost collection 6

Appendix 2: Table 2: Summary of resources included in community-wide mass drug administration and school-based deworming costing analyses, by routine and supportive program costs 7

Appendix 2: Table 3: Key costing inputs (non-exhaustive) for community-wide mass drug administration and school-based deworming, per country 9

Appendix 2: Table 4: Key assumptions regarding unit cost analysis for community-wide mass drug administration and school-based deworming 11

Appendix 3: Costs in local currency 13

Appendix 3: Table 1: Total economic costs and number of treatments administered through community-wide mass drug administration and school-based deworming, per country-round, in local currency 13

Appendix 3: Table 2: Average unit costs (2019 local currency) for community-wide mass drug administration across two years ... 14

Appendix 3: Table 3: Average unit costs (2019 local currency) for school-based deworming across two years 15

Appendix 3: Table 4: Annual costs of additional deworming program activities, including censuses, prevalence surveys, and coverage surveys, across two years of implementation (2019 local currency) 16

Appendix 4: Additional details of sensitivity analysis methodology 17

Appendix 5: Additional costing results 18

Appendix 5: Table 1: Percentage of DeWorm3 activity unit costs that are fixed vs. variable, by activity and country 18

Appendix 5: Figure 1: Total financial and opportunity costs of a) community-wide mass drug administration and b) school-based deworming by input-classification (including routine and supportive program costs). 19

Appendix 5: Table 2: Community-wide mass drug administration costs by input classification, by routine vs. supportive costs 20

Appendix 5: Table 3: School-based deworming costs by input classification, by routine vs. supportive costs 20

Appendix 5: Figure 2: Mean and range of unit costs per sub-activity across four rounds of community-wide mass drug administration in Benin, India, and Malawi; two rounds of school-based deworming in Benin and Malawi; and, four rounds of school-based deworming in India. 21

Appendix 5: Table 4: Annual costs of additional deworming program activities, including censuses, prevalence surveys and coverage surveys, across two years of implementation (2019 USD ($)) 22

Appendix 6: Description of cost differences across countries 23

Appendix 6: Table 1: Drivers of heterogeneity in costs across sites 23

Appendix 7: Appendix references 25

Morozoff C, *et al. BMJ Open* 2022; 12:e059565. doi: 10.1136/bmjopen-2021-059565

Appendix 1: Additional details on DeWorm3 activities implemented

In the below table, we provide additional details on how community-wide mass drug administration (cMDA) and school-based deworming (SBD) were implemented in each country.

Appendix 1: Table 1: Implementation characteristics of the DeWorm3 trial at study sites

	Benin	India	Malawi
Study location	Come Commune	Tamil Nadu State (Vellore and Thiruvanamalai districts)	Mangochi District
Implementing organizations	• Institut de Recherche Clinique du Benin<br>• Institut de Recherche pour le Développement<br>• Ministry of Health, Benin	• Christian Medical College, Vellore<br>• Ministry of Health and Family Welfare, New Delhi and Directorate of Public Health, Chennai	• Blantyre Institute for Community Outreach<br>• London School of Tropical Medicine and Hygiene<br>• Ministry of Health and Education, Malawi
cMDA strategy (20 intervention clusters)	• Bi-annual cMDA in all ages.<br>• Community drug distributors (volunteers) delivered drugs<br>• Implemented by DeWorm3	• Bi-annual cMDA in all ages; following National Deworming Day (described below)<br>• Community drug distributors (volunteers) delivered drugs<br>• Implemented by DeWorm3	• Bi-annual cMDA in all ages<br>• Health Surveillance Assistants, employed by the government, delivered drugs<br>• Implemented by DeWorm3
SBD strategy (20 intervention clusters and 20 control clusters)[a]	• SBD conducted annually<br>• Treatment of children 5-14 years old<br>• Implemented by the Ministry of Health (with a subcontract from DeWorm3)	• National Deworming Days, conducted bi-annually in schools and Anganwadi centers (pre-schools)<br>• Treatment of children 1-19 years old<br>• Implemented by the Ministry of Health and Family Welfare, New Delhi and Directorate of Public Health, Chennai	• SBD conducted annually, integrated with "Child Health Days"<br>• Community mop-up for non-enrolled children<br>• Treatment of children 1-14 years old<br>• Implemented by DeWorm3
Additional trial activities	• Planning meetings<br>• Annual census<br>• Two prevalence surveys<br>• Bi-annual coverage survey (after each round of MDA)	• Planning meetings<br>• Annual census<br>• Two prevalence surveys<br>• Bi-annual coverage survey (after each round of MDA)	• Planning meetings<br>• Annual census<br>• One prevalence survey<br>• Bi-annual coverage survey (after each round of MDA)

Acronyms: mass drug administration (MDA), community-wide MDA (cMDA), school-based deworming (SBD).

[a] SBD was implemented in the entire Dw3 study area (40 clusters) per each country's national deworming strategy, however SBD was only costed in control clusters (n=20).

Appendix 1: Table 2: Narrative description of DeWorm3 mass drug administration activities

MALAWI		
**Sub-activity**	**Community-wide mass drug administration (cMDA)**	**School-based delivery (SBD)**
Supply chain	• *Shipment to country*: Drugs were donated, ordered through the WHO. One shipment for both cMDA and SBD was made for 1.5 million doses and sent by ship, which supplied all years of the project. The stock was kept at the Central Drug Stores in Lilongwe and then dispensed to the study.<br>• *Storage and disbursement*: Albendazole for each MDA round was stored in the Deworm3 office in Namwera. During cMDA, albendazole was dispensed daily to the enumerators, and the remaining stock was returned to the office each evening. Drug supply was monitored using stock control cards and excel files of stock issued to enumerators.	• *Shipment:* Same shipment as cMDA.<br>• *Storage and disbursement*: Albendazole for each MDA round was stored in the Deworm3 office in Namwera. Field officers (employed by DeWorm3) transported the drugs between the office and schools during SBD.
Sensitization	Several committees and community boards were engaged for MDA sensitization. In year 2, the DeWorm3 team employed additional sensitization measures to improve community engagement and maximize treatment coverage. Activities included:<br>• Area Development Council meetings with group village headmen and/or representatives from Village Development Committees.<br>• Village-level community meetings were conducted by Health Surveillance Associates (HSAs) and volunteers.<br>• Village dramas and public announcements (year 2 only).<br>• Religious and Traditional Authority leaders of the Community Advisory Board visited communities that displayed signs of community tension or low participation to resolve any communication issues (year 2 only).	Sensitization for SBD was combined with cMDA sensitization activities.
Training	• *Health staff and volunteers*: DeWorm3 field officers trained HSAs at health centers and halls. Training sessions were one day long, though they were conducted over the course of two days to accommodate all health center staff. Afterward, HSAs oriented volunteers.<br>• *Enumerators:* The DeWorm3 trial coordinator and field officers trained enumerators for two days, followed by a three-day pilot of data collection instruments used during MDA.	• *Health staff and volunteers:* Training for SBD was combined with cMDA training activities.<br>• *Enumerators:* Training for SBD was combined with cMDA training activities.<br>• *Teachers and other school staff:* Training of teachers and principal education assistants was conducted by field officers supported by the Ministry of Health STH Programme Manager.
Drug delivery	Drug delivery was conducted twice per year, in intervention clusters only (n=20), by teams of enumerators, HSAs, and volunteers. HSAs were responsible for a relatively large number of households. HSAs supervised volunteers (about 4 volunteers per HSA). Enumerators were driven daily from Namwera to the community with their drug stocks, and HSAs were picked up along the way. Area Development Council members helped in mobilizing the community on the day of MDA.	School-based deworming was conducted once per year in all DeWorm3 clusters (n=40); in intervention clusters, SBD was conducted prior to cMDA. Treatment was administered at each school by the link HSA, with administrative support from two schoolteachers and the headteacher.<br><br>Children were also treated for schistosomiasis, using praziquantel. Costs of praziquantel were excluded from this costing analysis.
Supervision	Supervision was conducted by the DeWorm3 trial coordinator, DeWorm3 field officers, local health officers (Environmental Health Officers, Assistant Environmental Health Officers, District Environmental Health Officer, District Health Officer), District Council Representative, District STH Coordinator, and the Ministry of Health STH Programme Manager.	Supervision was conducted by the DeWorm3 trial coordinator, DeWorm3 field officers, local health officers (Environmental Health Officers, Assistant Environmental Health Officers, District Health Officer), District Council Representative, District STH Coordinator, Primary Education Authorities, and a representative from the Ministry of Education.
Mop-up	Malawi did not have a distinct mop-up period for cMDA. Instead, progress on coverage was tracked by a DeWorm3 monitoring dashboard, informed by electronic data collection forms. MDA was only considered complete once the dashboard indicated that all households had been treated or visited three times; all individuals who were absent from the household, but not migrated, at the first visit were followed up at least two further times.<br><br>Mop-up costs were estimated in the analysis as approximately 1-2 days of work, to indicate the individuals who were followed up with more than once.	Village level MDA of children who weren't in school was conducted as "mop-up" for two days after SBD.

INDIA		
**Sub-activity**	**Community-wide mass drug administration (cMDA)**	**School-based delivery (SBD)**
Supply chain	• *Shipment to country*: Drugs were donated, ordered through the WHO. Drugs were ordered centrally by the Ministry of Health and Family Welfare, through the national NTD program.<br>• *Storage and disbursement*: Consignment was brought to the central DeWorm3 office in Vellore and subsequently delivered to two subsite field offices. DeWorm3 field supervisors managed the tablets and provided them to fieldworkers daily to take to the villages for community drug distributors (CDDs) to dispense. The remaining tablets were returned to the office at end of the day.	• *Shipment to country*: Same shipment as cMDA.<br>• *Storage and disbursement*: Consignment was brought to the central DeWorm3 office in Vellore, and subsequently delivered to two subsite field offices. Field supervisors managed the tablets and provided them to Village Head Nurses to supply all schools and Anganwadi Centers.
Sensitization	• National Deworming Day sensitization materials were adapted to include information on cMDA; 1000 posters and 200 banners were posted in villages.<br>• Community sensitization meetings were conducted by DeWorm3 field staff using locally designed flipbooks to explain how STH are transmitted and what activities would be undertaken during cMDA.	Cloth banners provided by the government were put up by school staff outside schools and Anganwadi Centers one day before SBD.
Training	• *Health staff and volunteers*: CDDs participated in a half-day training, conducted by the DeWorm3 medical officer.<br>• *Enumerators:* DeWorm3 fieldworkers were trained by the DeWorm3 trial coordinator and data manager, followed by a short pilot period to test forms used during MDA.	• *Health staff and volunteers:* Training for SBD was combined with cMDA training activities.<br>• *Enumerators:* Training for SBD was combined with cMDA training activities.<br>• *Teachers and other school staff:* Workshops were held for teachers, Anganwadi Workers, and Village Health Nurses at every primary health center, conducted by respective primary health center medical officers.
Drug delivery	Drug delivery was conducted twice per year, in intervention clusters only (n=20), by teams of DeWorm3 fieldworkers (serving as enumerators) and CDDs, who walked door to door in the community. Nurses and medical officers supported with adverse events.	School-based deworming (called National Deworming Day) was conducted twice per year in all DeWorm3 clusters (n=40); in intervention clusters, SBD was conducted prior to cMDA. Drugs were delivered by Village Health Nurses in schools and Anganwadi Centers. ASHA workers and volunteers provided support as needed. DeWorm3 fieldworkers attended to deliver ink pens and treatment summary sheets. Nurses and medical officers helped with adverse events.
Supervision	Supervision was conducted by DeWorm3 field supervisors, DeWorm3 field managers, and local health workers (Village Head Nurses, Sector Health Nurses, and Community Health Nurses, and Block Medical Officers).	Supervision was conducted by local health workers (Village Head Nurses, Sector Health Nurses, and Community Health Nurses, and Block Medical Officers), central and sub-national level government health authorities.
Mop-up	After cMDA, a mop-up campaign was conducted for 1-4 days to reach absent individuals. Homes with absent individuals were visited up to three times.	One additional day of mop-up was conducted for children who were absent at school on National Deworming Day.

BENIN		
Sub-activity	**Community-wide mass drug administration (cMDA)**	**School-based delivery (SBD)**
Supply chain	• *Shipment to country*: Drugs were donated, ordered through the WHO. Drugs were ordered centrally by the Ministry of Health, for routine use, and stored in the national storage facility.<br>• *Storage and disbursement:* Drugs were dispatched to the zonal referral hospital in Come, by the National Communicable Disease Control Program (Programme National de Lutte contre les Maladies Transmissibles or PNLMT). Afterward, drugs were transferred to each health center affiliated with DeWorm3, with transit supervised by head doctors at the commune level. Nurses collected drugs for the MDA campaign from the referral hospital after training. Nurses then dispensed drugs to CDDs for cMDA. After cMDA, the remaining drugs were transported from clusters to the central level.	• *Shipment to country*: Same shipment as cMDA.<br>• *Storage and disbursement*: Same disbursement process as cMDA, except nurses dispensed drugs to school headmasters rather than to CDDs.
Sensitization	• Information sessions were held with local authorities (town hall), leaders of opinion, religious leaders, professional associations, and town criers.<br>• Messages were passed to the community through town criers, radio broadcasts, specific groups (i.e. women's associations), and religious centers.<br>• Banners and posters were also placed in the community.	• Sensitization for SBD was combined with cMDA sensitization activities.<br>• Additional activities included: a meeting with the chief of the pedagogical region and his advisors (Ministry of Education responsible for Come commune), sensitization of teachers via meetings (year 2), and flyer distribution in schools.
Training	• *Health staff and volunteers:* Ministry of Health staff trained 10 head health personnel (health center nurses, Chief Medical Officer, and District Medical Coordinator). Head nurses then trained CDDs. Supervision of training was done by PNLMT technical staff, doctors, and some district and departmental level staff.<br>• *Enumerators:* DeWorm3 staff trained enumerators and controllers (supervisors of enumerators).	• *Health staff and volunteers:* Training was combined with cMDA.<br>• *Teachers and school staff:* Ministry of Education officials and school headmasters were trained by 4 PNLMT staff and 2 DeWorm3 staff.
Drug distribution	Drug delivery was conducted twice per year, in intervention clusters only (n=20). Drugs were distributed by CDDs, joined by an enumerator, with the assumption that each CDD/enumerator pair would treat 60 people per day.	School-based deworming was conducted once per year in all DeWorm3 clusters (n=40); in intervention clusters, SBD was conducted prior to cMDA. Teachers administered drugs to children attending school. School directors/headmasters supervised and reported. CDDs treated non-enrolled children, who were invited to go to the closest school. Enumerators observed and filled out a treatment register.
Supervision	Supervision was conducted by DeWorm3 staff, central PNLMT staff, departmental staff, District Chief Doctors, and sub-district health center nurses.	The same supervisory staff as cMDA.
Mop-up	Two days of mop-up was conducted as needed. There was no mop-up in round 1 of cMDA. In round 4, flooding interrupted cMDA, and extensive mop-up was conducted.	No mop-up period.

Acronyms: World Health Organization (WHO), mass drug administration (MDA), community-wide mass drug administration (cMDA), school-based deworming (SBD), soil-transmitted helminths (STH), neglected tropical diseases (NTD), Programme National de Lutte contre les Maladies Transmissibles (PNLMT).

Appendix 2: Additional details on costing methodology

In the following tables, we provide additional details on the DeWorm3 costing methodology, including details on data collection tools and key model assumptions.

Appendix 2: Table 1: DeWorm3 instruments for cost collection

Source	Primary use	Type of cost	Content
DeWorm3 costing tool	Estimate resource use and costs of activities implemented by the DeWorm3 team	Financial and opportunity	Resource line items, corresponding prices, quantities, and expenditure recorded by sub-activity; separate modules for start-up and implementation
Activity table	Understand the purpose of resource use and how costs from the DeWorm3 costing tool relate to the implementation of activities	Financial and opportunity	Description of operational activities and sub-activities, number of project staff and other resources used, number of days
Activity calendar	Allocate shared costs to activities based on time spent on activities, such as staff salaries	N/A	Start, end dates, and duration of operational activities
Ministry of health costing tools	Estimate government costs of school-based deworming in DeWorm3 study area and Ministry of Health involvement in cMDA	Financial and opportunity	Budgets for routine school-based deworming at the national or state level across countries, government-funded employee salaries, and time spent on activities
MDA forms (i.e. digital treatment forms)	Estimate the number of persons treated, and time spent delivering treatments, to determine time spent by CDDs in each household	Opportunity costs, cost per person treated	Time spent per household to deliver community MDA; the number of persons treated
Census	Calculate relative DeWorm3 population size to district or state, in order to allocate district or state level costs to study area	N/A	DeWorm3 population size, control (SBD) and intervention (cMDA) cluster population size and demographic indicators such as age
School survey	Estimate teacher-related costs	Opportunity	Number of teachers trained, number of teachers involved in SBD, and time spent on activities
Literature	Collect relevant information where gaps persist	Financial and opportunity	District and state population sizes, number of schools per district/state, costs of equipment already owned

Acronyms: community-wide mass drug administration (cMDA), mass drug administration (MDA), school-based deworming (SBD), community drug distributor (CDD)

Appendix 2: Table 2: Summary of resources included in community-wide mass drug administration and school-based deworming costing analyses, by routine and supportive program costs

	Routine program costs	Supportive program costs
**Planning**		
Definition	None.	Start-up planning costs for DeWorm3, including developing IEC materials, mobile data collection forms, recruitment, and planning meetings with stakeholders.
Financial costs	None.	DeWorm3 salaries; travel, per-diems, and materials for planning meetings.
**Program management**		
Definition	Estimated operating costs to conduct routine program activities.	Estimated operating costs to conduct supportive program activities such as additional supervision and electronic data collection.
Financial costs	Salaries and overheads for DeWorm3 staff managing the project, including planning and reporting, building rent and utilities, equipment such as computers, vehicles, etc. Borrowed or pre-owned items, annualized across useful life years.	Same as routine program costs.
Opportunity costs	Time costs for government staff involved in the management of deworming programs.	None.
**Community sensitization**		
Definition	Sensitization activities varied across sites and also varied between school-based deworming and community-wide mass drug administration. For a complete list of activities conducted in each country, please see Appendix 1: Table 2. Examples include meetings with local committees/authorities/leaders, engagement with village chiefs, village dramas, door-to-door sensitization, posters and banners, radio advertisements, public criers.	Activities beyond those expected in routine programs, such as sensitizing the community to DeWorm3 research activities.
Financial costs	Per-diems and travel allowances, meeting costs such as refreshments and chair rentals, sensitization materials.	Examples include meeting costs for a Community Advisory Board, resources to hold a soccer competition/community event, and additional teacher sensitization.
Opportunity costs	Time costs for government-funded staff involved in sensitization (Health Surveillance Associates). Uncompensated time for volunteer staff who were involved in sensitization, such as community drug distributors. Time is valued using average national or regional salaries.	None.
**Training**		
Definition	Resources to train community drug distributors, volunteers, and health workers involved in drug delivery.	Resources to train enumerators involved in electronic data collection, as well as additional supervision by deworm3 implementing partners.
Financial costs	Per-diems, printed materials, refreshments, and hall rental.	Per-diems, printed materials, refreshments, and hall rental.

Opportunity costs	Time costs for government-funded staff involved in training (e.g., teachers, supervisors). Uncompensated time for volunteer staff who were trained, such as community drug distributors (Benin and India), ASHAs (India), and volunteers (Malawi). Time is valued using average national or regional salaries.	None.
**Drug delivery**		
Definition	Resources to deliver drugs either in the community or at schools, including mop-up.	Additional resources for enumerators to collect electronic monitoring data, and for supervision by deworm3 implementing partners.
Financial costs	Fuel, car rentals and per-diems for government supervisors, allowances/incentives for drug distributors, drugs for adverse events.	Per-diems, mobile allowances for uploading data, fuel, and car hires.
Opportunity costs	Time costs for government-funded staff involved in drug delivery (e.g., teachers, supervisors). Uncompensated time for volunteer staff, such as community drug distributors (Benin and India), ASHAs (India), and volunteers (Malawi). Time is valued using average national or regional salaries. Costs of donated drugs.	None.

Appendix 2: Table 3: Key costing inputs (non-exhaustive) for community-wide mass drug administration and school-based deworming, per country

	**Benin**	**India**	**Malawi**	**Data source**
**DeWorm3 study site**				
Number villages	52	401	113	DeWorm3 Village Registry
Baseline population	94,969	140,932	121,819	DeWorm3 Census
**Days of drug delivery, including mop-up**				
cMDA: mean days (min-max)	12 (11—15)	13 (11—16)	16 (16)	DeWorm3 activity list
SBD: mean days (min-max)	2 (2)	2 (2)	5 (5)	DeWorm3 activity list
**Drug costs**				
Albendazole: opportunity cost, per tablet	$0.05	$0.01	$0.05	Benin and Malawi: GSK[1], India: National Deworming Day financial guidelines[a]
**Drug Distributors (CDDs and HSAs)**				
Staff involved: mean (min-max)	90 (90)	127 (114—164)	56 (56)	DeWorm3 costing tool
Monthly salary: approximate	$125	$126	$203	Benin: ILO[2]; India: State salary estimates[3], Malawi: DeWorm3 Ministry of Health costing survey
Time spent on cMDA training and sensitization: days	2	2	2	DeWorm3 costing tool
Time spent per cMDA visit, including travel: median minutes	17	11	14	DeWorm3 MDA forms
Number of cMDA visits conducted per drug distributor, per round: median visits	181	177	328	DeWorm3 MDA forms
Daily allowances[b] for drug delivery	$3.41	$3.55	$5.37	DeWorm3 costing tool
**Teachers[c]**				
Number of schools: median (min-max)	55 (54—55)	254 (228—298)	35 (29—40)	SBD forms
Teachers involved in SBD: mean (min-max)	304 (288—320)	339 (331—347)	147 (121—173)	DeWorm3 costing tool and school survey
Monthly salary: approximate[d]	$380	$456	$203	DeWorm3 Ministry of Health costing survey
Time spent on training and reporting: median days	0.25	0.625	0.5	Ministry of Health costing tool
Time spent on drug delivery: median days	0.33	0.25	1	DeWorm3 school survey
Allowances given	None	Per training	Per training, per day of drug delivery	Ministry of Health costing tool, DeWorm3 costing tool
Allowance rate	—	$1.42	$5.41	DeWorm3 costing tool
**Other school staff[c]**				
Position	School Directors	Anganwadi Workers	—	DeWorm3 costing tool and school survey
Number staff involved in SBD: mean (min-max)	55 (54—55)	126 (124—127)	—	DeWorm3 costing tool and school survey
Monthly salary: approximate	$539	$188	—	DeWorm3 Ministry of Health costing survey

Time spent on training and reporting: median days	1.5	0.625	—	Ministry of Health costing tool
Time spent on drug delivery: median days	2	0.25	—	DeWorm3 school survey
Allowances given	Per training	Per training	—	Ministry of Health costing tool, DeWorm3 costing tool
Allowance rate	$17.01	$1.42	—	DeWorm3 costing tool
**DeWorm3 Enumerators**				
Number staff involved: mean (range)	90 (90)	84 (73—107)	57 (50—65)	DeWorm3 costing tool
Daily compensation and allowances	$8.55	$6.50	$14—$34[e]	DeWorm3 costing tool
**DeWorm3 Field Supervisors (Controllers, Field Supervisors, Field Officers)**				
Number staff involved	10	13	4	DeWorm3 costing tool
Daily compensation and allowances: approximate	$21	$12	$20	DeWorm3 costing tool
**DeWorm3 Vehicle Costs**				
Project vehicles	2	1	5	DeWorm3 costing tool
Make of vehicles	Nissan 4x, 5-seater	Mahindra Thar CRDe	Land cruiser 4.2 Diesel 13-seater	DeWorm3 costing tool
Net cost	$37,807	$13,755	$41,137	DeWorm3 costing tool
Useful life years assumed	9	9	9	WHO CHOICE
**DeWorm3 Central Personnel**				
DeWorm3 central key program staff (providing program management and higher-level supervision) involved	11	11	8	DeWorm3 costing tool
DeWorm3 central support staff (drivers, accountants, etc.) involved	10	7	10	DeWorm3 costing tool

Acronyms: community-wide mass drug administration (cMDA), school-based deworming (SBD), Gross Domestic Product (GDP), National Deworming Day (NDD), GlaxoSmithKline (GSK)
Note: Dashes (–) represent situations where no data was observed (e.g. no allowances given, no staff involved).
[a] GlaxoSmithKline (GSK) is currently donating albendazole for lymphatic filariasis and soil-transmitted helminth control. The estimated opportunity costs of donated albendazole is $0.045 per tablet. We have also included the cost of shipping, raising the total estimated costs to $0.047. Costs per tablet administered also include 10% wastage, bringing the total to 0.052. Although GSK-donated albendazole was used in the DeWorm3 project, this analysis used the estimated costs of locally procured albendazole in India, as is routinely used in National Deworming Days. Estimated cost per tablet of locally procured albendazole was acquired from the Tamil Nadu State Budget for National Deworming Day.
[b] Type of allowance varied per country (i.e., lunch allowance, mobile data, travel allowance, etc.). Given the travel nature of the work, and the descriptions of these costs, we have chosen to present these costs as allowances rather than compensation for work done. In some countries, the allowances vary based on number of days involved or number of persons reached.
[c] Information on schools, teachers, and other school staff is specific to control clusters only within the DeWorm3 study. Although SBD was implemented within all clusters in the DeWorm3 study (n=40) per each country's national deworming strategy, SBD was only costed within control clusters (n=20).
[d] Salary varies based on level of school.
[e] Some nurses functioned as enumerators were paid a higher rate.

Morozoff C, *et al. BMJ Open* 2022; 12:e059565. doi: 10.1136/bmjopen-2021-059565

Appendix 2: Table 4: Key assumptions regarding unit cost analysis for community-wide mass drug administration and school-based deworming

Type of cost	Description of costs	Assumptions	Analysis decisions
Trial/research costs	Costs related to conducting the trial component of DeWorm3, such as trial insurance, developing IRB materials, etc.	Trial-related costs exclusively related to research did not affect MDA coverage.	Trial-related costs were excluded from cMDA and SBD unit cost analyses.
Planning costs	Activities related to starting up the trial such as micro-planning, recruitment, procurement, trial sensitization meetings, and development of IEC and training materials.	Planning was relevant to all field activities (census, prevalence survey, cMDA, SBD, and coverage survey).	Planning costs were annualized over 3 years of program implementation and split across activities based on the number of days activities were implemented. When monthly or annual costs needed to be split by days, we assumed 20.5 workdays per month.
Program management costs	Program management costs were fixed costs and included large capital items, rent, and salaried project staff. Program management resources were used in multiple trial activities, (generally) purchased/ employed/ rented/ donated in the planning stages of the trial, and were retained for the duration of the trial.	Program management was relevant to all field activities (Census, prevalence survey, cMDA, SBD, and coverage survey).<br><br>There may have been inefficiencies in resource use. For example, a vehicle that was purchased by DeWorm3 may not be driven every day.<br><br>Some materials that were already owned by the DeWorm3 team would need to be purchased by future implementing organizations.	Capital items were annualized over their useful life years, with a 3% discount rate.<br><br>Costs were split among annual activities based on the number of days spent on each activity. When monthly or annual costs needed to be split by days, we assumed 20.5 workdays per month.<br><br>When costs were shared among multiple programs within the implementing institution, we allocated a percentage of costs towards DeWorm3 (i.e. only a portion of total rent costs for an implementing organization were allocated to DeWorm3, if the organization had multiple grants/projects). When resources were used only by the DeWorm3 project, we assumed full costs of resources, even if not used at full capacity.<br><br>Resources that were already owned by the DeWorm3 team (i.e., vehicles, computers, etc.) were categorized as financial costs in this analysis.
Census costs	All costs to run an annual census conducted prior to MDA in all 40 clusters.	Censuses did not affect MDA coverage.	Census costs were excluded from the cMDA and SBD unit cost analysis and were presented separately.
Prevalence survey costs	An annual prevalence survey was used to assess STH prevalence across the 40 clusters.	In year 1, a longitudinal monitoring cohort (LMC) of approximately 6,000 persons was conducted, in addition to a cross-sectional survey of 20,000 persons, per country. In year 2, only the longitudinal monitoring cohort was conducted in Benin and India (no prevalence surveys were conducted in Malawi year 2). It is therefore assumed that approximately 1/4 of shared prevalence survey costs were relevant to the LMC, and 3/4 to the cross-sectional survey, in year 1.<br><br>Prevalence surveys did not affect MDA coverage.	We have presented only the costs of the LMC in this manuscript. Approximately ¼ of shared prevalence survey costs in year 1 were allocated to the LMC.<br><br>Prevalence survey costs were excluded from the cMDA and SBD unit cost analysis and were presented separately.
Coverage surveys	All costs related to conducting post-MDA coverage surveys: conducted after each round of cMDA, sampling approximately 8,000 individuals from the 40 clusters.	Coverage surveys did not affect cMDA coverage.	Coverage survey costs were excluded from the cMDA and SBD unit cost analysis and were presented separately.
DeWorm3 vehicle costs	DeWorm3 project vehicles and related costs (fuel, maintenance, etc), as well as hired vehicles.	Project and hired vehicles were used for additional supervision by DeWorm3 field staff and enumerator transport.	DeWorm3 project vehicles and hired vehicles used in cMDA and SBD were designated as "supportive" costs unless specified as a routine cost (i.e., vehicle hired for government supervisor, fuel reimbursement for training participant, etc.).

Shared MDA (cMDA/SBD) costs	Resources or costs that were described as shared between cMDA and SBD.	In rounds where cMDA was implemented directly after SBD, many sensitization activities were relevant to both cMDA and SBD.<br><br>In rounds where cMDA was implemented directly after SBD, most training activities were relevant to both cMDA and SBD.	Shared costs were split between cMDA and SBD proportionally based on the number of days of each activity (for example, for training costs), or by population treated (for example, for side effects medication).
Input classification for per-diems and allowances	Costs that were described as per-diems or allowances to implementers, trainers, supervisors, or community members.	Unless specified that costs were incentives or compensation, allowances and per-diems were assumed to be used for their designated purpose (for example, lunch allowances used to purchase lunch, travel allowances used for transport).	Per-diems and allowances that were specified as transport allowances, were assigned “vehicles and overheads” as the input classification.<br><br>Per-diems and allowances that were not specified as transport allowances, were assigned “wages and per-diems” as the input classification.<br><br>Unless specified that costs were incentives or compensation, allowances and per-diems were not considered compensation and were not subtracted from estimated opportunity costs. For example, if CDDs were provided a lunch or travel allowance during fieldwork, this was not considered compensation for work done.

Acronyms: community-wide mass drug administration (cMDA), mass drug administration (MDA), school-based deworming (SBD), community drug distributor (CDD), longitudinal monitoring cohort (LMC).

Appendix 3: Costs in local currency

In the following tables, we present key costing data from the manuscript, presented in local currency. Costs are presented in 2019 West African Francs (XOF) for Benin, 2019 Indian Rupees (INR) for India, and 2019 Malawian Kwacha (MWK) for Malawi.

Appendix 3: Table 1: Total economic costs and number of treatments administered through community-wide mass drug administration and school-based deworming, per country-round, in local currency

Metric	Benin (XOF)		India (INR)		Malawi (MWK)	
	cMDA	SBD	cMDA	SBD	cMDA	SBD
Number of treatments administered[a]						
Round 1	45,280	–	55,953	15,266	49,518	–
Round 2	37,913	9,298	55,758	19,152	38,641	16,077
Round 3	42,398	–	57,353	21,396	52,122	–
Round 4	32,529	10,343	57,398	20,586	49,709	12,964
Total costs[b]						
Round 1	61,148,760	–	5,068,089	975,635	94,544,024	–
Round 2	47,069,592	12,912,370	4,536,205	992,149	71,212,140	16,716,181
Round 3	57,116,293	–	4,656,850	900,980	70,936,162	–
Round 4	54,342,916	14,882,855	4,352,396	898,775	72,724,580	17,999,058
Cost per treatment administered						
Round 1	1,350	–	91	64	1,909	–
Round 2	1,242	1,389	81	52	1,843	1,040
Round 3	1,347	–	81	42	1,361	–
Round 4	1,671	1,439	76	44	1,463	1,388

Acronyms: community-wide mass drug administration (cMDA), school-based deworming (SBD)
Note: Dashes (–) represent situations where no data was collected. SBD was only implemented annually in Benin and Malawi, so no data were available for rounds 1 and 3.
[a] Treatments administered for cMDA includes all eligible individuals who received treatment by DeWorm3 through cMDA in the intervention clusters (Source: DeWorm3 MDA treatment logs). Population treated for SBD includes all children treated in schools within the DeWorm3 control clusters (Source: SBD treatment logs).
[b] Total costs include both financial and opportunity costs.

Appendix 3: Table 2: Average unit costs (2019 local currency) for community-wide mass drug administration across two years
Total economic costs are presented, as well as a breakdown of costs by routine vs. supportive activities, and financial vs. opportunity costs

	Benin (XOF)[a]	India (INR)[a]	Malawi (MWK)[a]
**Planning**	**61**	**3**	**5**
Supportive (financial)	61	3	5
**Program management**	**371**	**28**	**376**
Routine (financial)	164	11	113
Routine (opportunity) – time costs for government staff[b]	6	–	0
Supportive (financial)	200	17	263
**Community sensitization**	**143**	**12**	**125**
Routine (financial)	64	1	46
Routine (opportunity) – time costs for government staff and volunteers	8	0	27
Supportive (financial) – additional sensitization activities	4	0	10
Supportive (financial) – NGO supervision	66	10	42
**Training costs**	**196**	**8**	**190**
Routine (financial)	70	0	49
Routine (opportunity) – time costs for government staff and volunteers	14	2	18
Supportive (financial) – training of electronic data collectors	63	4	41
Supportive (financial) – NGO supervision and training support	50	1	82
**Drug delivery**	**631**	**32**	**948**
Routine (financial)	210	5	152
Routine (opportunity) – time costs for government staff and volunteers	90	8	136
Routine (opportunity) – donated drugs	0	1	0
Supportive (financial) – electronic data capture	172	13	228
Supportive (financial) – NGO supervision	159	5	433
Average unit costs[c]	***1402***	***82***	***1644***
Routine (financial)	*509*	*18*	*360*
Routine (opportunity)	*118*	*11*	*181*
Supportive (financial)	*776*	*53*	*1103*

Acronyms: non-governmental organization (NGO)
Note: Dashes (–) represent situations where no costs were observed.
[a] Analysis includes two years of cMDA. As cMDA was conducted bi-annually in each country, results are presented as the average across four rounds.
[b] Government staff include supervisory and implementing staff whose salaries are paid by the Ministry of Health. Examples include nurses and health officers, HSAs (Malawi only), as well as national and subnational government officials involved in the program.
[c] Routine and supportive activities and related resources are described in Appendix 2: Table 2. Financial costs represent actual expenditure on goods and services purchased by the government or NGO implementing partner. Opportunity costs, on the other hand, include costs forgone by using a resource in a particular way. These opportunity costs recognize and value the cost of using resources, as these resources are then unavailable for productive use elsewhere. Opportunity costs in this analysis include costs of donated albendazole, volunteer time spent on the project (such as volunteer drug distributors), and estimated government staff salary costs.

Appendix 3: Table 3: Average unit costs (2019 local currency) for school-based deworming across two years
Total economic costs are presented, as well as a breakdown of costs by routine program vs. supportive program activities, and financial vs. opportunity costs

	Benin (XOF)[a]	India (INR)[b]	Malawi (MWK)[a]
**Planning**	**43**	**0**	**4**
Supportive (financial)	43	0	4
**Program management**	**406**	**14**	**299**
Routine (financial)	–	–	110
Routine (opportunity) – time costs for government staff[c]	146	8	1
Supportive (financial)	260	6	187
**Community sensitization**	**153**	**0**	**89**
Routine (financial)	83	0	29
Routine (opportunity) – time costs for government staff and volunteers	–	–	39
Supportive (financial) – additional sensitization activities	27	–	5
Supportive (financial) – NGO supervision	44	–	15
**Training costs**	**357**	**13**	**189**
Routine (financial)	157	1	57
Routine (opportunity) – time costs for government staff and volunteers	119	10	83
Supportive (financial) – training of electronic data collectors	34	1	18
Supportive (financial) – NGO supervision and training support	47	1	31
**Drug delivery**	**454**	**23**	**634**
Routine (financial)	72	1	165
Routine (opportunity) – time costs for government staff and volunteers	329	20	130
Routine (opportunity) – donated drugs	0	1	0
Supportive (financial) – electronic data capture	9	1	154
Supportive (financial) – NGO supervision	44	0	186
Average unit costs[d]	***1414***	***50***	***1214***
Routine (financial)	*311*	*2*	*360*
Routine (opportunity)	*594*	*38*	*253*
Supportive (financial)	*508*	*10*	*601*

Acronyms: non-governmental organization (NGO)
Note: Dashes (–) represent situations where no costs were observed.
[a] Analysis includes two years of SBD. In India, SBD was conducted bi-annually, so results are presented as the average across four rounds.
[b] Analysis includes two years of SBD. In Malawi and Benin, SBD was conducted annually, so results are presented as the average of two rounds.
[c] Government staff include supervisory and implementing staff whose salaries are paid by the Ministry of Health. Examples include nurses and health officers, teachers, and national and subnational government officials involved in the program.
[d] Routine and supportive activities and related resources are described in Appendix 2: Table 2. Financial costs represent actual expenditure on goods and services purchased by the government or NGO implementing partner. Opportunity costs, on the other hand, include costs forgone by using a resource in a particular way. These opportunity costs recognize and value the cost of using resources, as these resources are then unavailable for productive use elsewhere. Opportunity costs in this analysis include costs of donated albendazole, volunteer time spent on the project (such as volunteer drug distributors), and estimated government staff salary costs.

Appendix 3: Table 4: Annual costs of additional deworming program activities, including censuses, prevalence surveys, and coverage surveys, across two years of implementation (2019 local currency)

Activity	Metric	Benin (XOF)		India (INR)		Malawi (MWK)	
		Year 1	Year2	Year 1	Year2	Year 1	Year2
Census[a]	Population censused	94,969	88,647	140,932	146,321	121,819	119,418
	Total cost	100,718,503	58,290,985	6,521,106	5,603,244	151,397,733	101,756,373
	Cost per person censused	1,061	658	46	38	1,243	852
Prevalence survey[a]	Population surveyed	6,814	5,283	6,503	6,158	6,935	–
	Total cost	74,426,119	67,624,070	5,487,075	12,480,670	93,810,394	–
	Cost per person surveyed	10,923	12,800	844	2,027	13,527	–
Coverage survey[a]	Population surveyed	16,339	16,130	15,573	14,809	16,796	17,166
	Total cost	44,466,307	38,946,531	1,459,531	1,392,526	51,862,081	41,869,871
	Cost per person surveyed	2,721	2,415	94	94	3,088	2,439

[a]For activities that spanned all 40 clusters, about 50% of the individuals surveyed were from intervention clusters, and the other 50% from control clusters.
Note: Dashes (–) represent situations where no data was collected. A prevalence survey was not conducted in Malawi in year 2.

Appendix 4: Additional details of sensitivity analysis methodology

One-way sensitivity analyses: In one-way sensitivity analyses, opportunity costs of drugs, opportunity costs of volunteer time, and coverage rates were explored.

Opportunity costs for albendazole in the costing analysis were valued using the estimated valuation of donated albendazole from GlaxoSmithKline (GSK) plus estimated shipping costs in Malawi and Benin, and the market price of locally procured albendazole in Tamil Nadu, India.[1] To date, GSK has committed to donating albendazole to combat STH until 2025.[4] After 2025, the cost of albendazole to STH programs is unknown. In sensitivity analyses, costs of albendazole were explored by removing opportunity costs as the low input (to explore financial costs to governments during albendazole donation programs) and doubling the opportunity costs of albendazole as the high input (doubling the global valuation of donated albendazole and doubling the India market price to explore how increases in albendazole costs could affect unit costs).

Opportunity costs for volunteers' time in the costing analysis were valued using national (Benin, Malawi) and subnational (India) average wage rates acquired from labor surveys.[5-6] In sensitivity analyses, volunteer time costs were altered by removing opportunity costs for the low input (with the assumption that lunch and travel allowances were sufficient forms of compensation). For the high input, opportunity costs for community volunteers who played a health-delivery role were valued using the estimated salaries of an equivalent health worker.[7]

Total treatments administered per country-round were used in the costing analysis. In sensitivity analyses, coverage rates (and therefore total treatments administered) were altered by applying the highest and lowest observed cluster coverage in a given country to the eligible population for treatment, demonstrating the observed ranges in coverage possible in a given location.

Two-way sensitivity analyses: Two-way sensitivity analyses were also conducted to determine the influence of reductions in supportive activities or sensitization activities alongside reductions in coverage.

The DeWorm3 Project prioritized high coverage of cMDA, intending to reach 90% coverage in each cluster.[8] To do so, the project employed additional supervision and electronic data collection to track coverage in real-time (e.g. "supportive activities"), and respond with mop-up in low coverage areas. These additional activities were resource-intensive, and may not be included in future routine programs. However, removing these additional activities may affect program coverage. In sensitivity analyses, we have explored a two-way analysis where cMDA routine costs are removed, and cMDA coverage rates were reduced by 30% to align more closely with historic MDA coverage rates.[8] Additionally, although SBD is routinely implemented by the governments of India, Benin, and Malawi, the interventions were altered to different extents for delivery during DeWorm3. For example, in Malawi, SBD was implemented through the DeWorm3 project team, rather than via the government of Malawi, leading to different program management costs. In Benin, the government implemented SBD, though the DeWorm3 team provided additional support in the form of supervision and additional sensitization. In sensitivity analyses, SBD coverage was reduced 10% alongside the removal of supportive activities, to reflect how these supportive activities might be increasing coverage during the trial. The relationship between supportive activity costs and coverage rates has not been validated, and future analyses may explore additional changes to input values.

To reach a goal of 90% coverage in each cluster, the DeWorm3 project implemented multiple community sensitization efforts that may have gone above and beyond activities implemented by the government. In two-way sensitivity analyses, the relationship between sensitization costs and coverage rates was explored. For the high-input: sensitization costs were increased 30% with an increase of 10% in coverage rates (not exceeding 100% coverage of eligible populations). For the low-input, sensitization costs were decreased 30% with a decrease of 10% in coverage rates. The relationship between sensitization costs and coverage rates has not been validated, and future analyses may explore additional changes to input values.

Future directions for sensitivity analyses: Given the many costing resources that were included in this analysis, there are many possibilities of costs that could be altered in sensitivity analyses. Decisions regarding which sensitivity analyses to conduct in this study were based upon field team and expert input regarding influential factors, and differences in implementation across DeWorm3 sites. Future discussions with government stakeholders may provide opportunities to explore how costs may vary in scaled-up programs (e.g. specific allowances for CDDs, frequency and resources needed for training, days of MDA, etc.) allowing for tailored sensitivity analyses.

Morozoff C, *et al. BMJ Open* 2022; 12:e059565. doi: 10.1136/bmjopen-2021-059565

Appendix 5: Additional costing results

In the following tables and figures, we present supplemental costing data not presented in the manuscript, including a further breakdown of costs by supportive vs routine activities, fixed vs variable inputs, and costs across rounds.

Appendix 5: Table 1: Percentage of DeWorm3 activity unit costs that are fixed vs. variable, by activity and country

	Benin		India		Malawi	
activity	Fixed unit cost	Variable unit cost	Fixed unit cost	Variable unit cost	Fixed unit cost	Variable unit cost
**Census1**	26%	74%	27%	73%	15%	85%
**Census2**	59%	41%	28%	72%	26%	74%
**Coverage survey1**	25%	75%	27%	73%	13%	87%
**Coverage survey2**	27%	73%	32%	68%	15%	85%
**Coverage survey3**	16%	84%	28%	72%	17%	83%
**Coverage survey4**	14%	86%	28%	72%	17%	83%
**Prevalence survey1**	34%	66%	40%	60%	14%	86%
**Prevalence survey2**	63%	37%	42%	58%	–	–
**cMDA1**	23%	77%	23%	77%	16%	84%
**cMDA2**	25%	75%	26%	74%	17%	83%
**cMDA3**	33%	67%	23%	77%	19%	81%
**cMDA4**	26%	74%	24%	76%	18%	82%
**SBD1**	–	–	7%	93%	–	–
**SBD2**	16%	84%	9%	91%	19%	81%
**SBD3**	–	–	8%	92%	–	–
**SBD4**	21%	79%	8%	92%	19%	81%

Acronyms: community-wide mass drug administration (cMDA), school-based deworming (SBD)
Note: Dashes (–) represent situations where no data was collected. SBD was only implemented annually in Benin and Malawi, so no data were available for rounds 1 and 3.

Appendix 5: Figure 1: Total financial and opportunity costs of a) community-wide mass drug administration and b) school-based deworming by input-classification (including routine and supportive program costs).

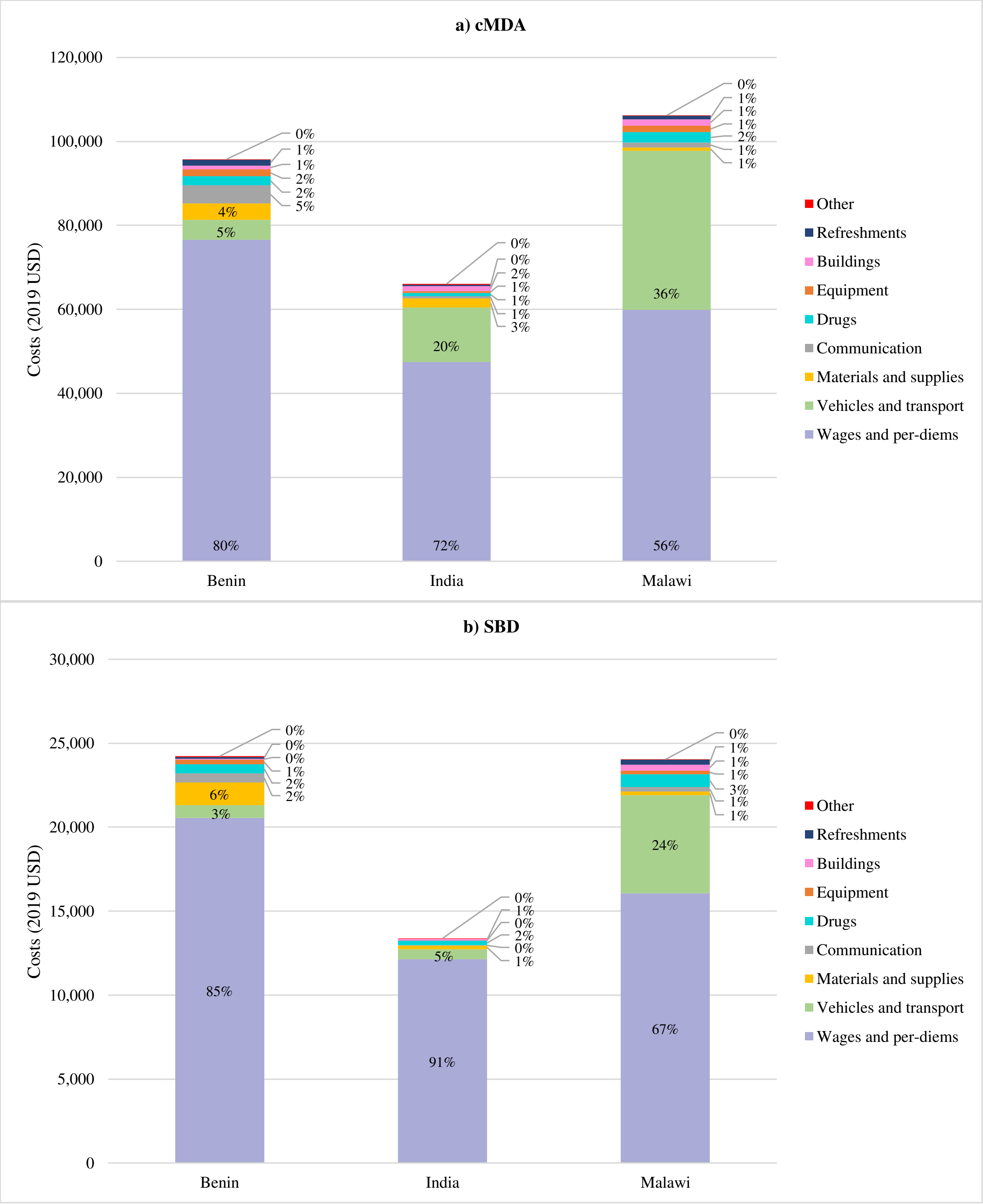

Note: For simplicity, labels were rounded to the nearest whole number. In situations where 0% is listed, category costs contributed less than 0.5% of total costs.

Appendix 5: Table 2: Community-wide mass drug administration costs by input classification, by routine vs. supportive costs

Category	Benin		India		Malawi	
	Routine	Supportive	Routine	Supportive	Routine	Supportive
Buildings and overheads	1%	1%	2%	2%	1%	1%
Communication	5%	5%	1%	1%	1%	1%
Drugs	0%	–	3%	–	0%	–
Equipment and overheads	2%	2%	1%	1%	3%	1%
Materials and supplies	7%	2%	6%	2%	1%	1%
Other	0%	0%	0%	0%	0%	0%
Refreshments	2%	1%	1%	0%	2%	0%
Vehicles and overheads	5%	5%	4%	28%	13%	48%
Wages and per-diems	78%	85%	81%	66%	78%	48%

Note: Dashes (–) represent situations where no costs were observed.

Appendix 5: Table 3: School-based deworming costs by input classification, by routine vs. supportive costs

Category	Benin		India		Malawi	
	Routine	Supportive	Routine	Supportive	Routine	Supportive
Buildings and overheads	0%	1%	0%	3%	1%	2%
Communication	2%	3%	–	1%	1%	1%
Drugs	0%	–	2%	–	0%	–
Equipment and overheads	0%	3%	–	1%	1%	1%
Materials and supplies	7%	3%	1%	2%	1%	1%
Other	0%	0%	0%	0%	0%	0%
Refreshments	0%	0%	–	–	2%	0%
Vehicles and overheads	2%	5%	0%	21%	11%	40%
Wages and per-diems	88%	85%	96%	71%	83%	55%

Note: Dashes (–) represent situations where no costs were observed.

Appendix 5: Figure 2: Mean and range of unit costs per sub-activity across four rounds of community-wide mass drug administration in Benin, India, and Malawi; two rounds of school-based deworming in Benin and Malawi; and, four rounds of school-based deworming in India.

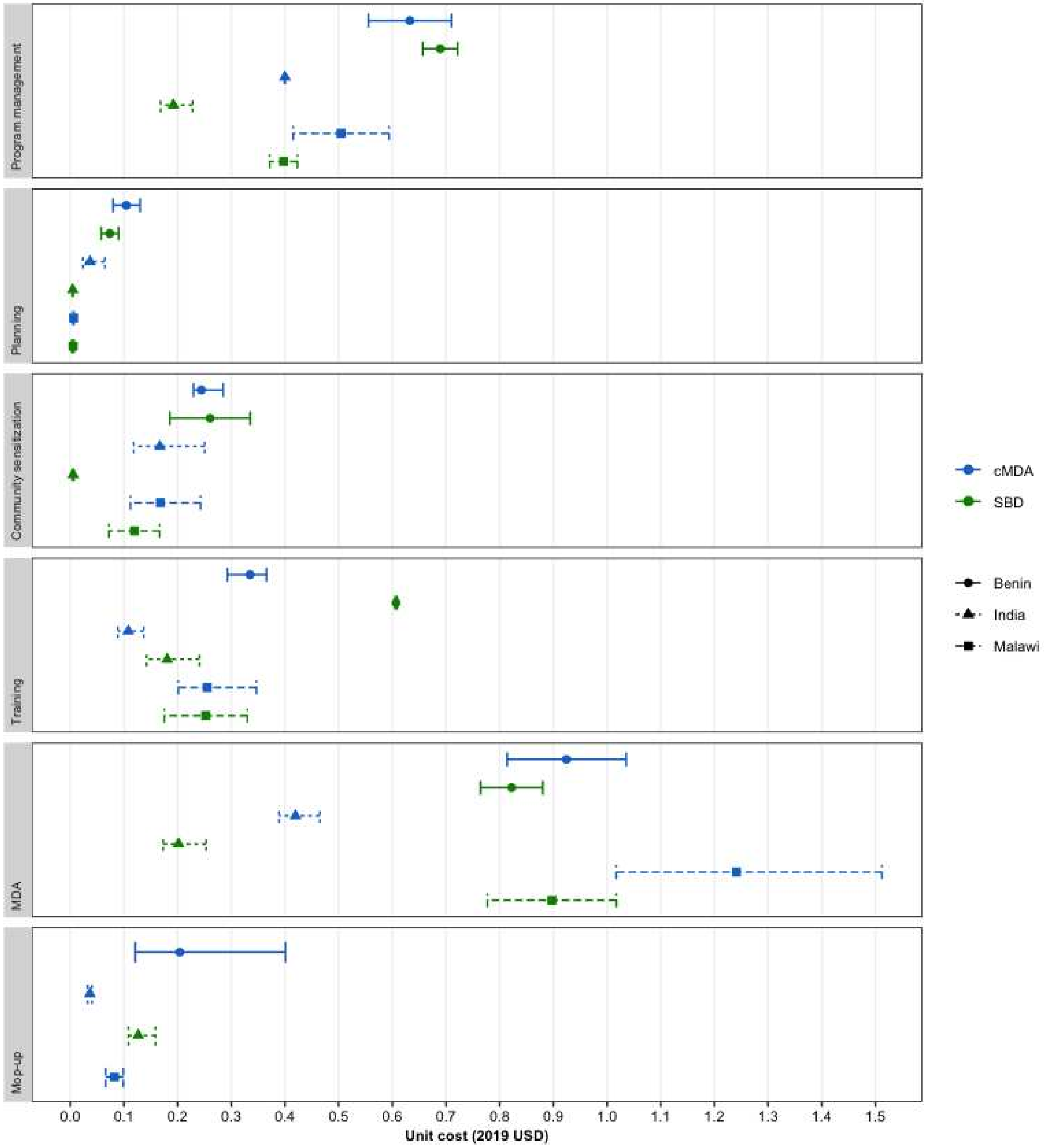

Acronyms: community-wide mass drug administration (cMDA), school-based deworming (SBD)

Appendix 5: Table 4: Annual costs of additional deworming program activities, including censuses, prevalence surveys and coverage surveys, across two years of implementation (2019 USD ($))

	Benin: Year 1	Benin: Year 2	India: Year 1	India: Year 2	Malawi: Year 1	Malawi: Year 2
**Annual census**[a]						
Population censused	94,969	88,647	140,932	146,321	121,819	119,418
Total cost	$ 171,889	$ 99,481	$ 92,603	$ 79,569	$ 203,071	$ 136,487
Cost per person censused	$ 1.81	$ 1.12	$ 0.66	$ 0.54	$ 1.67	$ 1.14
**Annual prevalence survey** following a cohort of approximately 5,000-7,000 individuals[a]						
Population surveyed	6,814	5,283	6,503	6,158	6,935	—
Total cost	$ 127,018	$ 115,409	$ 77,919	$ 177,231	$ 125,829	—
Cost per person surveyed	$ 18.64	$ 21.85	$ 11.98	$ 28.78	$ 18.14	—
**Bi-annual coverage survey** conducted after each round of cMDA, sampling approximately 8,000 individuals[a]						
Population surveyed	16,339	16,130	15,573	14,809	16,796	17,166
Total cost	$ 75,887	$ 66,467	$ 20,726	$ 19,774	$ 69,563	$ 56,160
Cost per person surveyed	$ 4.64	$ 4.12	$ 1.33	$ 1.34	$ 4.14	$ 3.27

[a] Activity spanned all 40 clusters, with about 50% of the individuals surveyed were from intervention clusters, and the other 50% from control clusters.
Note: Dashes (–) represent situations where no data was collected. A prevalence survey was not conducted in Malawi in year 2.

Appendix 6: Description of cost differences across countries

In the following tables, we provide further details and reasoning regarding differences in observed cMDA and SBD costs across countries.

Appendix 6: Table 1: Drivers of heterogeneity in costs across sites

Type of difference	Description	Differences in costs across countries
Number of persons targeted and treated	The number of persons censused in each study site varied, with the smallest population in Benin (approx. 90,000), followed by a larger population in Malawi (approx. 120,000), and the largest censused population in India (approx. 145,000). These differences in overall population sizes affected the total number of persons targeted and treated via community-wide mass drug administration (cMDA).<br><br>Additionally, the age composition in each site varied, leading to variability in the number of targeted children for school-based delivery (SBD). The percent of the population that was pre-school or school-aged was lowest in India, followed by Benin, and highest in Malawi. The number of children who were treated through SBD therefore varied across sites.	The number of persons treated may have affected overall costs per round of treatment, as more resources may have been required to reach a larger targeted population.<br><br>The number of persons treated had a large effect on the unit costs (cost per treatment administered). For example, the total costs per round of SBD were similar in Malawi and Benin (see Table 1, main text), however, the unit costs were much lower in Malawi given the larger number of children treated via SBD (approximately 50% more children were treated in Malawi than in Benin).
Costs per input-classification	Vehicle costs in Malawi were substantially higher than in Benin and India. Reasons for higher costs include more project vehicles (5 total vehicles were used in Malawi, compared to 2 vehicles in Benin and 1 in India). Additionally, the Malawi DeWorm3 team chose to organize travel for enumerators and HSAs centrally, requiring more car hires and fuel. When cars and drivers were hired to support activities, they were hired for more days in Malawi than in other countries, as MDA was generally 3-4 days longer in Malawi (see Appendix 2: Table 3). In India and Benin, enumerators and CDDs were provided travel allowances, which resulted in lower overall vehicle costs.	When examining cMDA and SBD costs by input-classification (Appendix 5: Figure 1), Malawi had a substantially higher percentage of costs that were allocated to vehicles and overheads, compared to India and Benin. Total costs per round of cMDA were generally highest in Malawi, in partial, due to vehicle costs. The highest cost of cMDA was observed in Malawi round 1, driven by a larger number of vehicles rented.
Planning and program management costs	Resources for planning and program management varied across sites.<br><br>More time was spent on planning in Benin, leading to higher planning costs. Additionally, full-time equivalent costs for central DeWorm3 staff were higher in Benin, leading to higher program management costs.<br><br>Involvement of the DeWorm3 team in SBD varied across countries, and therefore the share of DeWorm3 program management costs allocated to SBD varied across countries. In India, SBD was implemented by the government through the bi-annual National Deworming Day (NDD). Therefore, the DeWorm3 team was only minorly involved in SBD delivery, mainly to observe and record data. In Malawi, the DeWorm3 team was solely responsible for implementing SBD in DeWorm3 clusters, with light supervision from the government. In Benin, the implementation of SBD was led by the government, however, the DeWorm3 team was heavily involved in the coordination and supervision.	cMDA planning and supervision costs were highest in Benin, followed by Malawi, and lowest in India, generally driven by wages (Benin) and vehicles (Malawi).<br><br>Program management costs for SBD were much lower in India, given the DeWorm3 team provided less implementation support and supervision compared to the other countries.
Opportunity costs for albendazole	Albendazole used in the DeWorm3 project was donated, however, common practice in costing analyses is to estimate the opportunity costs of drugs (i.e., the costs of the drugs if they were used for other purposes, rather than donated). Albendazole is locally produced in India, so we estimated opportunity costs in India using the local per-tablet price. In Benin and Malawi, albendazole is procured from global suppliers. We estimated opportunity costs in Benin and Malawi as the GlaxoSmithKline valuation of donated albendazole, as $0.045 per-tablet, plus the estimated costs of shipping at $0.0019, for a total value of $0.052.[1] We also estimated approximately 10% buffer stock.	Opportunity costs of albendazole are lower in India than in Benin and Malawi, resulting in about a $0.04 difference in unit costs.

Opportunity costs for government-funded staff and volunteers	The number, type, salaries, and time involved for currently employed government staff and volunteers varied across settings.<br><br>CDDs and volunteers: In Malawi, the primary drug distributors (HSAs), were government-funded staff whereas the primary drug distributors in Benin and India (CDDs) were volunteers. Fewer HSAs were involved in Malawi compared to CDDs in Benin and India, however, salaries were higher in Malawi.<br><br>Teachers and school staff: Malawi had the fewest number of teachers and school staff involved in drug delivery, with more staff involved in Benin, and the greatest number in India. School directors were also involved in SBD in Benin. However, the time spent by teachers on delivery varied, with the smallest amount of time in India, and the greatest amount in Malawi. Monthly salaries for teachers in Benin and India were similar; teacher salaries were approximately 50% lower in Malawi. See Appendix 2: Table 3 for more details.<br><br>Government supervisors: Fewer government staff were involved in the supervision of SBD and cMDA in Malawi, as the DeWorm3 team was the primary implementer.	Opportunity costs for government staff and volunteers were similar across countries for cMDA.<br><br>A large number of school staff in Benin and India (including additional involvement of Anganwadi Workers in India and school directors in Benin) and higher teachers' salaries led to higher staff and volunteer opportunity costs for SBD. School staff opportunity costs were lowest in Malawi.
Financial resources for community sensitization	Community sensitization activities varied across sites.<br><br>Benin activities included community-level meetings, public criers, radio broadcasts, and printed materials. Benin also included teacher sensitization for SBD in year 2.<br><br>India activities focused on distributing printed materials (banners and flyers), and community-level meetings (cMDA only).<br><br>Malawi activities included community-level meetings, public announcements, drama groups, and a football bonanza (round 4).<br><br>See Appendix 1: Table 2, for more details.	Sensitization costs were highest in Benin, due to more activities implemented.<br><br>Costs for SBD sensitization were substantially lower in India, as costs were only related to printed materials.
Financial resources for training	In India, SBD is routinely conducted bi-annually and resources for implementation are kept quite low. The only routine financial costs reported by the government were transport allowances provided to teachers. The DeWorm3 team's involvement in SBD training was minimal.<br><br>In Benin and Malawi, the DeWorm3 team was involved in training, and therefore more financial costs were incurred such as printed materials, refreshments, and equipment and hall hires for training sessions.<br><br>Similarly, Benin and Malawi used more financial resources such as equipment, mobile minutes, and refreshments for cMDA training, compared to India.	Financial costs for SBD and cMDA training were substantially lower in India, likely because it was completely government-led.
Financial resources for drug delivery: SBD	In India, SBD is routinely conducted bi-annually and resources for implementation are kept quite low. In the DeWorm3 project, SBD continued to be implemented through the government routinely. Few financial resources are required during drug delivery, only allowances for some key staff (VHNs, ASHAs, and for supervision).<br><br>In Benin and Malawi, the DeWorm3 team was more involved in drug delivery. Therefore, more resources were used such as fuel, allowances (e.g. for travel, communication, lunch) for CDDs/HSAs, refreshments, and allowances for DeWorm3 coordinating and supervisory staff.	Financial costs for SBD were substantially lower in India, likely because it was completely government-led with involvement from DeWorm3 limited to data collection.
Financial resources for drug delivery: cMDA	In Benin, the DeWorm3 team collaborated closely with the Ministry of Health to implement cMDA. Therefore, many allowances and travel costs were incurred for supervision and coordination efforts by both the Ministry of Health and the DeWorm3 team. Additionally, cMDA mop-up required more resources in Benin, mainly due to a large mop-up campaign in cMDA round 4. In Benin, cMDA round 4 was interrupted by a natural disaster (flooding). To reach higher coverage rates, a more involved mop-up campaign was implemented one month after MDA, with additional sensitization and training.<br><br>In India and Malawi, the DeWorm3 was primarily responsible for cMDA drug delivery, with few allowances paid to government supervisors.	Routine financial costs were higher in Benin, due to more supervision costs and the more involved mop-up campaign in round 4 of MDA.

Appendix 7: Appendix references

1 Turner HC, Bettis AA, Chu BK, et al. Investment Success in Public Health: An Analysis of the Cost-Effectiveness and Cost-Benefit of the Global Programme to Eliminate Lymphatic Filariasis. *Clin Infect Dis* 2017; **64**: 728–35.

2 International Labor Organization. ILOSTAT database. https://ilostat.ilo.org/data (accessed Aug 25, 2021).

3 Tamil Nadu State Government Department of Evaluation and Applied Research. Tamil Nadu - An Economic Appraisal 2011-12 to 2013-14.

4 GlaxoSmithKline. Neglected Tropical Diseases: Our commitment. 2021; published online Jan. https://www.gsk.com/media/6543/ntd-factsheet-jan2021.pdf.

5 International Labor Organization. ILOSTAT database. https://ilostat.ilo.org/data (accessed Aug 25, 2021).

6 Tamil Nadu State Government Department of Evaluation and Applied Research. Tamil Nadu - An Economic Appraisal 2011-12 to 2013-14.

7 Serje J, Bertram MY, Brindley C, et al. Global health worker salary estimates: an econometric analysis of global earnings data. *Cost Eff Resour Alloc* 2018; **16**: 10.

8 Werkman M, Toor J, Vegvari C, et al. Defining stopping criteria for ending randomized clinical trials that investigate the interruption of transmission of soil-transmitted helminths employing mass drug administration. *PLoS Negl Trop Dis* 2018; **12**: e0006864.
