# Supplementary material for: Costs of community-wide mass drug administration and school-based deworming for soil-transmitted helminths: evidence from a randomised controlled trial in Benin, India and Malawi
Source: BMJ Open. 2022 Jul 8;12(7):e059565. doi: 10.1136/bmjopen-2021-059565 (PMC9272108; doi:10.1136/bmjopen-2021-059565)
Supplement: Author's manuscript [file bmjopen-2021-059565.draft_revisions.pdf]

BMJ Open is committed to open peer review. As part of this commitment we make the peer review history of every article we publish publicly available.

When an article is published we post the peer reviewers' comments and the authors' responses online. We also post the versions of the paper that were used during peer review. These are the versions that the peer review comments apply to.

The versions of the paper that follow are the versions that were submitted during the peer review process. They are not the versions of record or the final published versions. They should not be cited or distributed as the published version of this manuscript.

BMJ Open is an open access journal and the full, final, typeset and author-corrected version of record of the manuscript is available on our site with no access controls, subscription charges or pay-per-view fees (http://bmjopen.bmj.com).

If you have any questions on BMJ Open's open peer review process please email info.bmjopen@bmj.com

BMJ Open

Costs of community-wide mass drug administration and school-based deworming for soil-transmitted helminths: evidence from Benin, India, and Malawi

Journal:	*BMJ Open*
Manuscript ID	bmjopen-2021-059565
Article Type:	Original research
Date Submitted by the Author:	23-Nov-2021

Complete List of Authors:	Morozoff, Chloe; University of Washington, Global Health<br>Avokpaho, Euripide; Institut de Recherche Clinique du Benin<br>PUTHUPALAYAM KALIAPPAN, SARAVANAKUMAR; Christian Medical College Vellore, Division of Gastrointestinal Sciences<br>Simwanza, James; Blantyre Institute for Community Outreach<br>Gideon, Samuel; Christian Medical College Vellore, Division of Gastrointestinal Sciences<br>Lungu, Wongani; Blantyre Institute for Community Outreach<br>Houngbegnon, Parfait; Institut de Recherche Clinique du Benin<br>Galactionova, Katya; Swiss Tropical and Public Health Institute; University of Basel<br>Sahu, Maitreyi; University of Washington, Global Health<br>Kalua, Khumbo; Blantyre Institute for Community Outreach<br>Luty, Adrian J. F.; Universite de Paris, MERIT, IRD<br>Ibikounle, Moudachirou; Institut de Recherche Clinique du Benin; Universite d'Abomey-Calavi, Centre de Recherche pour la lutte contre les Maladies Infectieuses Tropicales (CReMIT/TIDRC)<br>Bailey, Robin; London School of Hygiene & Tropical Medicine, Clinical Research Department<br>Pullan, Rachel; London School of Hygiene & Tropical Medicine, Department of Disease Control<br>Ajjampur, Sitara; Christian Medical College Vellore, Division of Gastrointestinal Sciences<br>Walson, Judd; University of Washington, Global Health<br>Rubin Means, Arianna; University of Washington, Global Health

Keywords:	Health economics < HEALTH SERVICES ADMINISTRATION & MANAGEMENT, Tropical medicine < INFECTIOUS DISEASES, Public health < INFECTIOUS DISEASES

SCHOLARONE™
Manuscripts

I, the Submitting Author has the right to grant and does grant on behalf of all authors of the Work (as defined in the below author licence), an exclusive licence and/or a non-exclusive licence for contributions from authors who are: i) UK Crown employees; ii) where BMJ has agreed a CC-BY licence shall apply, and/or iii) in accordance with the terms applicable for US Federal Government officers or employees acting as part of their official duties; on a worldwide, perpetual, irrevocable, royalty-free basis to BMJ Publishing Group Ltd ("BMJ") its licensees and where the relevant Journal is co-owned by BMJ to the co-owners of the Journal, to publish the Work in this journal and any other BMJ products and to exploit all rights, as set out in our licence.

The Submitting Author accepts and understands that any supply made under these terms is made by BMJ to the Submitting Author unless you are acting as an employee on behalf of your employer or a postgraduate student of an affiliated institution which is paying any applicable article publishing charge ("APC") for Open Access articles. Where the Submitting Author wishes to make the Work available on an Open Access basis (and intends to pay the relevant APC), the terms of reuse of such Open Access shall be governed by a Creative Commons licence – details of these licences and which Creative Commons licence will apply to this Work are set out in our licence referred to above.

Other than as permitted in any relevant BMJ Author's Self Archiving Policies, I confirm this Work has not been accepted for publication elsewhere, is not being considered for publication elsewhere and does not duplicate material already published. I confirm all authors consent to publication of this Work and authorise the granting of this licence.

For peer review only

Costs of community-wide mass drug administration and school-based deworming for soil-transmitted helminths: evidence from Benin, India, and Malawi

Chloe Morozoff, MPH[1], Euripide Avokpaho, MD[2], Saravanakumar Puthupalayam Kaliappan, PhD[3], James Simwanza, BA[4], Samuel Paul Gideon, MBA[3], Wongani Lungu, ACCA[4], Parfait Houngbegnon, PhD[2], Katya Galactionova, PhD[5,6], Maitreyi Sahu, MSPH[1], Khumbo Kalua, PhD[4], Adrian J F Luty, PhD[7], Moudachirou Ibikounlé, PhD[2,8], Robin Bailey, PhD[9], Rachel Pullan, PhD[10], Sitara Swarna Rao Ajjampur, PhD[3], Judd Walson, MD[1], Arianna Rubin Means, PhD[1*]

[1] Department of Global Health, University of Washington, Seattle, Washington, United States
[2] Institut de Recherche Clinique du Benin, Abomey-Calavi, Benin
[3] The Wellcome Trust Research Laboratory, Division of Gastrointestinal Sciences, Christian Medical College, Vellore, India
[4] Blantyre Institute for Community Outreach, Blantyre, Malawi
[5] Swiss Tropical and Public Health Institute, Basel, Switzerland
[6] University of Basel, Basel, Switzerland
[7] Université de Paris, MERIT, IRD, Paris, France
[8] Centre de Recherche pour la lutte contre les Maladies Infectieuses Tropicales (CReMIT/TIDRC), Université d'Abomey-Calavi, Bénin
[9] Clinical Research Department, London School of Hygiene & Tropical Medicine, London, United Kingdom
[10] Faculty of Infectious and Tropical Diseases, London School of Hygiene and Tropical Medicine, London, United Kingdom

For peer review only

***Corresponding author:**
Arianna Rubin Means, PhD
Hans Rosling Center Box 351620
3980 15th Ave NE, Seattle, WA 98195
Tel: +1 (206) 370 0225 | Email: aerubin@uw.edu

Word count:
Abstract: 300
Main text: 3,609

ABSTRACT

Objectives: Current guidelines for the control of soil-transmitted helminths (STH) recommend deworming children and other high-risk groups, primarily using school-based deworming (SBD) programs. However, targeting individuals of all ages through community-wide mass drug administration (cMDA) may interrupt STH transmission in some settings. We compared the costs of cMDA to SBD to inform decision making about future updates to STH policy.

Design: We conducted activity-based micro-costing of cMDA and SBD for two years in Benin, India, and Malawi within an ongoing cMDA trial.

Setting: Field sites and collaborating research institutions.

Primary and secondary outcomes: We calculated total financial and opportunity costs and costs per treatment administered (unit costs) from the service provider perspective, including costs related to community drug distributors and other volunteers.

Results: On average, cMDA unit costs were more expensive than SBD in India ($1.17 vs. $0.72) and Malawi ($2.26 vs. $1.69), and comparable in Benin ($2.45 vs. $2.47). cMDA was more expensive than SBD in part because most costs (~60%) were "supportive costs" needed to deliver treatment with high coverage, such as additional supervision and electronic data capture. A smaller fraction of cMDA costs (~30%) were routine expenditures (e.g. drug distributor allowances). The remaining cMDA costs (~10%) were opportunity costs of staff and volunteer time. A larger percentage of SBD costs were opportunity costs for teachers and other government staff (between ~25%-75%). Unit costs varied over time and were sensitive to the number of treatments administered.

Conclusions: cMDA was generally more expensive than SBD. Accounting for local staff time (volunteers, teachers, health workers) in community programs is important and drives higher cost estimates than commonly recognized in the literature. Costs may be lower outside of a trial setting, given a reduction in supportive costs used to drive higher treatment coverage and economies of scale.

Trial registration number: NCT03014167.

For peer review only

STRENGHTS AND LIMITATIONS OF THIS STUDY

- We used rigorous micro-costing methods to collect costs associated with cMDA and SBD and corresponding treatment data, in real time.
- The granularity of data collected provides rich information regarding the resource needs for deworming programs, and how these may vary across countries and delivery modalities (school versus community-based treatment).
- We estimated opportunity costs of the volunteer workforce and currently employed government staff (e.g. teachers, community drug distributors, supervisors), which are often excluded from deworming costing studies.
- Although costs associated with research and trial administration were not included in this study, it is possible that some costs (e.g., program management, planning, and supervision) may be higher in this research setting than what would be observed in routing deworming programs.

For peer review only

INTRODUCTION

Soil-transmitted helminths (STH) are a group of intestinal parasites (*Ascaris lumbricoides*, *Ancylostoma duodenale*, *Necator americanus*, and *Trichuris trichiura*) that globally affect approximately 1.5 billion individuals annually, predominantly in sub-Saharan Africa, East Asia, and Latin America.[1] Moderate-to-heavy infection with STH is associated with diarrhea, malnutrition, anemia, wasting, stunting, and cognitive delay.[1-2] To reduce the burden of STH morbidity, the World Health Organization (WHO) targets elimination of STH as a public health problem by 2030.[3] Current STH control guidelines recommend preventative chemotherapy (deworming using albendazole or mebendazole) for high-risk populations such as children, non-pregnant adolescent girls, and women of reproductive age.[2]

STH control programs include annual or bi-annual school-based deworming (SBD), where teachers and health workers deliver preventative chemotherapy to pre-school and school-aged children.[2] SBD is a low-cost intervention; SBD leverages existing infrastructure (schools) as a delivery platform while drug costs are low due to global drug donation programs.[4] A review of STH treatment costs estimates SBD costs at US $0.30 per child treated, much lower than the cost of screening and treating a single individual for STH annually ($4.89/person).[2] Costs of deworming preschool-aged children or other community members outside of schools, is estimated at $0.63 per person treated.[2,5] Although SBD is a low-cost intervention for controlling STH, non-school attending children may be missed by these programs, and reinfection of children within the community from adult reservoirs may require continuous treatment.[6]

It may be possible to interrupt STH transmission by expanding deworming eligibility to individuals of all ages.[7] The DeWorm3 Project is a cluster-randomized trial testing the feasibility of interrupting STH transmission using community-wide mass drug administration (cMDA) in Benin, India, and Malawi.[8-9] If successful, scaling-up cMDA would require evidence on the relative cost compared to standard-of-care SBD. This study systematically identified, measured, and compared resources for implementation of twelve rounds of cMDA and eight rounds of SBD across the DeWorm3 sites. Determining the costs and cost drivers of expanding STH treatment to all individuals in a community will be essential for shaping future STH policy.

METHODS

Overview of DeWorm3

In each site (Benin, India, Malawi), twenty control clusters were randomized to SBD (either annually or bi-annually, per the country's standard of care) and twenty intervention clusters were randomized to biannual cMDA. During treatment rounds in which SBD was also implemented, cMDA was conducted after SBD (see Figure 1). During SBD, teachers distributed albendazole to children, with support from community health workers, known as Community Drug Distributors (CDDs) in Benin, Accredited Social Health Activists (ASHAs) in India, and Health Surveillance Assistants (HSAs) in Malawi. During cMDA, albendazole was delivered door-to-door in the community by community health workers (CDDs in Benin, CDDs and ASHAs in India, HSAs in Malawi) alongside electronic data collectors, referred to as enumerators. For both treatment strategies, supervision was conducted by DeWorm3 and government staff. Differences in delivery across sites are described in Appendix 1.

In addition to deworming, DeWorm3 also conducted an annual census to enumerate the full population in study catchment areas, annual prevalence surveys to determine STH prevalence and intensity, and post-MDA coverage surveys to assess the reach of cMDA and SBD (Figure 1).[8] These activities are not consistently conducted in national deworming programs but could be indicated in future elimination programs that require more intensive monitoring and evaluation.

For peer review only

Costing study design

We conducted activity-based micro-costing from the service provider perspective (Ministry of Health and/or Education) during the first two years of DeWorm3 implementation (2017-2019) in order to explore heterogeneity in costs across rounds of implementation. This analysis includes opportunity costs associated with all health worker involvement in implementation, including teachers and community volunteers engaging in drug delivery. Costs to the household were not assessed as they are assumed to be negligible.[10-11] We measured all resources required to deliver cMDA and SBD and converted their value into a cost estimate (including borrowed and donated resources).[12-13]

The methodology for costing cMDA is detailed in Galactionova et al, 2021, and additional SBD data collection tools are detailed in Appendix 2.[14] We briefly describe the strategy used. DeWorm3 staff in each site recorded resource use and costs related to the implementation of trial activities within an Excel-based costing tool. Data were collected in real-time and were entered separately for activities including program management (overheads), planning, and each round of the census, prevalence survey, SBD, cMDA, and coverage survey. Within the tool, we also quantified borrowed resources used, such as borrowed vehicles and volunteer time. Other data sources were used to collect or allocate costs not included in the costing tool, such as government expenditures (see Appendix 2).

Following data collection, all costing data were iteratively reviewed for quality and completeness. Costs related to DeWorm3 research only (e.g. qualitative research or school surveys) were not included in the data collection instruments and, if identified, were removed during data cleaning. [15]

Analyzing financial and opportunity costs

Financial costs included actual expenditures on goods and services purchased by the DeWorm3 project or site governments. We analyzed these data in Stata (version 16.1). Costs were converted to USD using the annual average exchange rate based on the year in which the costs were incurred.[16] When costs were shared across multiple activities—such as vehicles or personnel salaries—we allocated costs based on the number of days required to implement each sub-activity. We allocated costs reported at the district or state level via government budgets to the DeWorm3 study area using population proportionate to size estimates. We annualized startup costs over the three-year duration of cMDA and SBD implementation, and capital items based on their useful life years, using a 3% discount rate.[13,17] All costs are presented in 2019 USD; costs incurred before 2019 were inflated using GDP implicit price deflators.[18-19] Costs in local currency are presented in Appendix 4.

Opportunity costs included the costs of donated drugs, volunteer time (CDDs, ASHAs, and community volunteers), and time costs for currently employed government staff. We estimated costs associated with volunteer time spent delivering drugs using the DeWorm3 trial's digital treatment forms (described in Appendix 2). We used country-specific average earnings to estimate the opportunity costs associated with volunteer time (2010-2011 regional annual salary adjusted to relevant year using annual growth rate in India and 2018 national monthly earnings in Benin and Malawi).[20-21] For government staff (e.g. national and district-level personnel, teachers, and health center staff), we collected salaries through Ministry of Health costing surveys. We derived government staff time spent on activities from costing data collection tools, and teacher time spent on SBD from a school survey. We calculated total economic costs (financial plus opportunity costs) per site, per year, and by activity, sub-activity, and input-classification. Key costing inputs such as the number of implementing staff, average salaries, and allowances are described in Appendix 2.

Estimating routine and supportive program costs

Because the DeWorm3 Project included several activities related to the delivery and monitoring of MDA that may not be present in all deworming programs, we classified and distinguished costs as either routine

For peer review only

MDA program costs or supportive program costs. Routine program costs included activities typically implemented by a government (e.g. training of CDDs). Supportive costs included additional activities aimed at optimizing coverage and compliance. For example, electronic data were collected to monitor cMDA coverage in real-time and identify areas in need of additional sensitization and mop-up. Generally, supportive activities included: 1) start-up planning costs; 2) additional supervision from a non-governmental organization (NGO) implementing partner; 3) additional sensitization activities; 4) electronic data collection, and 5) program management costs associated with these supportive activities. Additional details regarding routine and supportive costs are presented in Appendix 2.

Unit costs analysis

The cost per treatment administered (i.e. unit cost) was determined by dividing costs per round by the total number of treatments administered. The number of treatments administered via cMDA was abstracted from MDA treatment forms (household-level forms completed by enumerators during cMDA). The number of treatments administered via SBD was estimated from paper SBD forms filled out by school and/or DeWorm3 field staff, then transferred to an electronic format. One and two-way sensitivity analyses were conducted to explore how the average cost per treatment administered would change due to variation in key costing inputs and coverage levels (methods described in Appendix 3).

Patient and public involvement

Community members living in STH endemic areas were not involved in the design, conduct, reporting, or dissemination of this costing study. Ministry of Health and Education staff were involved in the conduct of this costing study (including data collection and dissemination), and in the design and conduct of the wider DeWorm3 trial.

RESULTS

Total and unit costs of cMDA and SBD

Between February 2018 and December 2019, a total of twelve rounds of cMDA and eight rounds of SBD were delivered across DeWorm3 sites in Benin, India, and Malawi. Table 1 details the number of treatments administered, total costs, and unit costs across treatment strategies, sites, and rounds. The total number of treatments administered for a given round of MDA ranged from 9,298 (Benin SBD round 2) to 57,398 (India cMDA round 4). Total costs of SBD ranged from $12,763 in India (round 4) to $25,933 in Benin (round 4), while total costs of cMDA ranged from $61,806 (India, round 4) to $129,369 (Malawi, round 1). Sub-activity costs also varied across rounds, as detailed in Appendix 5.

cMDA unit costs varied from $1.08 in India (round 4) to $2.90 in Benin (round 4). Within sites, cMDA unit costs varied across the four rounds, fluctuating by $0.73 in Benin and Malawi, and $0.21 in India. SBD was generally less expensive than cMDA, with approximately one-third the number of treatments administered and one-quarter of the total costs. SBD unit costs varied from $0.60 in India (round 3) to $2.51 in Benin (round 4). Within sites, SBD costs fluctuated $0.09 across two rounds in Benin, $0.31 across four rounds in India, and $0.46 across two rounds in Malawi.

For peer review only

Table 1: Total economic costs and number of treatments administered through community-wide mass drug administration and school-based deworming, per country-round, in 2019 USD ($).

Metric	Benin		India		Malawi	
	cMDA	SBD	cMDA	SBD	cMDA	SBD
Number of treatments administered[a]						
Round 1	45,280	–	55,953	15,266	49,518	–
Round 2	37,913	9,298	55,758	19,152	38,641	16,077
Round 3	42,398	–	57,353	21,396	52,122	–
Round 4	32,529	10,343	57,398	20,586	49,709	12,964
Total costs[b]						
Round 1	106,695	–	71,969	13,854	129,369	–
Round 2	82,287	22,516	64,416	14,089	97,512	23,251
Round 3	99,664	–	66,129	12,794	97,838	–
Round 4	94,422	25,933	61,806	12,763	100,112	24,812
Cost per treatment administered						
Round 1	2.36	–	1.29	0.91	2.61	–
Round 2	2.17	2.42	1.16	0.74	2.52	1.45
Round 3	2.35	–	1.15	0.60	1.88	–
Round 4	2.90	2.51	1.08	0.62	2.01	1.91

Acronyms: community-wide mass drug administration (cMDA), school-based deworming (SBD)
[a] Treatments administered for cMDA includes all eligible individuals who received treatment by DeWorm3 through cMDA in the intervention clusters (Source: DeWorm3 MDA treatment logs). Population treated for SBD includes all children treated in schools within the DeWorm3 control clusters (Source: SBD treatment logs).
[b] Total costs include both financial and opportunity costs.

Activity-specific financial and opportunity costs

Activity-specific unit costs for cMDA and SBD are presented in Tables 2 and 3, respectively. Average cMDA unit costs were $2.45 in Benin, $1.17 in India, and $2.26 in Malawi. Routine financial costs were approximately 20-35% of total costs, at $0.87 in Benin, $0.26 in India, and $0.48 in Malawi. The majority of routine financial costs (approximately 70-80%) were allowances for key implementing staff (e.g., lunch, travel, and/or mobile allowances for CDDs, health center staff, district and national government supervisors, sensitization staff). The remaining portion of routine costs were materials and supplies, equipment or building rentals, and vehicle costs for supervision (Appendix 5). Routine opportunity costs, including donated drugs and government and volunteer time, were approximately 10% of unit costs (ranging from $0.16 in India to $0.30 in Malawi). Supportive program costs, including costs of electronic data collection with additional supervision in the DeWorm3 project, comprised the majority of total costs (approximately 60%).

On average, SBD unit costs were $2.47 in Benin, $0.72 in India, and $1.69 in Malawi. Routine financial costs, such as per-diems, fuel, and materials, were approximately 5-30% of total costs and were more expensive in Benin and Malawi ($0.53 and $0.48 respectively) as compared to India ($0.03). Routine opportunity costs, mainly teachers and school-level staff time, represented the largest share of costs in Benin and India (approximately 45% and 75% respectively); the governments of Benin and India were the primary SBD implementers. In Malawi, where SBD was delivered by the DeWorm3 team, routine opportunity costs were only 1/4 of costs whereas supportive activities represented half of total costs.

For peer review only

Across sites, total costs were generally higher for cMDA as compared to SBD, except for Benin. However, routine cMDA costs were consistently less expensive compared to SBD, driven in part by the high opportunity costs of SBD. Across cMDA and SBD, program management and drug delivery were the most expensive activities. Drug delivery included initial drug distribution, as well as mop-up activities (approximately 10-20% of drug delivery costs). The largest resource input was staff wages and per-diems, representing 56%-91% of total costs, followed by vehicle costs (Appendix 5). Routine vehicle costs were used for government supervision and transport for training. However, the majority of vehicle costs were used for supportive activities, mainly field staff supervision and transport of enumerators to field sites each day for mobile data collection. Vehicle costs contributed to a higher share of costs in Malawi, compared to other sites. Approximately 15% of SBD and 25% cMDA costs were fixed or capital costs (Appendix 5), meaning the expenses do not depend on the quantity of treatments delivered. Examples of fixed costs include program overheads such as rent, central staff salaries, etc.

For peer review only

Table 2: Average unit costs (2019 USD ($)) for community-wide mass drug administration across two years
Subtitle: Total economic costs are presented, as well as a breakdown of costs by routine vs. supporting activities, and financial vs. opportunity costs

	Benin[a]	India[a]	Malawi[a]
**Planning**	**$ 0.10**	**$ 0.04**	**$ 0.01**
Supporting (financial)	$ 0.10	$ 0.04	$ 0.01
**Program management**	**$ 0.63**	**$ 0.40**	**$ 0.50**
Routine (financial)	$ 0.28	$ 0.16	$ 0.15
Routine (opportunity) – time costs for government staff[b]	$ 0.01	—	< $ 0.01
Supporting (financial)	$ 0.34	$ 0.24	$ 0.35
**Community sensitization**	**$ 0.24**	**$ 0.17**	**$ 0.17**
Routine (financial)	$ 0.11	$ 0.02	$ 0.06
Routine (opportunity) – time costs for government staff and volunteers	$ 0.01	< $ 0.01	$ 0.04
Supporting (financial) – additional sensitization activities	$ 0.01	< $ 0.01	$ 0.01
Supporting (financial) – NGO supervision	$ 0.11	$ 0.14	$ 0.06
**Training costs**	**$ 0.34**	**$ 0.11**	**$ 0.26**
Routine (financial)	$ 0.12	$ 0.01	$ 0.07
Routine (opportunity) – time costs for government staff and volunteers	$ 0.02	$ 0.03	$ 0.02
Supporting (financial) – training of electronic data collectors	$ 0.11	$ 0.05	$ 0.05
Supporting (financial) – NGO supervision and training support	$ 0.08	$ 0.02	$ 0.11
**Drug delivery**	**$ 1.13**	**$ 0.46**	**$ 1.32**
Routine (financial)	$ 0.36	$ 0.07	$ 0.20
Routine (opportunity) – time costs for government staff and volunteers	$ 0.15	$ 0.11	$ 0.18
Routine (opportunity) – donated drugs	$ 0.05	$ 0.01	$ 0.05
Supporting (financial) – electronic data capture	$ 0.29	$ 0.19	$ 0.31
Supporting (financial) – NGO supervision	$ 0.27	$ 0.07	$ 0.58
***Total costs*[c]**	***$ 2.45***	***$ 1.17***	***$ 2.26***
Routine (financial)	*$ 0.87*	*$ 0.26*	*$ 0.48*
Routine (opportunity)	*$ 0.26*	*$ 0.16*	*$ 0.30*
Supporting (financial)	*$ 1.32*	*$ 0.75*	*$ 1.48*

Acronyms: non-governmental organization (NGO)
[a] Analysis includes two years of cMDA. As cMDA was conducted bi-annually in each country, results are presented as the average across four rounds.
[b] Government staff include supervisory and implementing staff who's salaries are paid by the ministry of health. Examples include: nurses and health officers, HSAs (Malawi only), as well as national and subnational government officials involved in the program.
[c] Routine and supporting activities and related resources are described in Appendix 2. Financial costs represent actual expenditure on goods and services purchased by the government or NGO implementing partner. Opportunity costs, on the other hand, include costs forgone by using a resource in a particular way. These opportunity costs recognize and value the cost of using resources, as these resources are then unavailable for productive use elsewhere. Opportunity costs in this analysis include: costs of donated albendazole, volunteer time spent on the project (such as volunteer drug distributors), and estimated government staff salary costs.

For peer review only

Table 3: Average unit costs (2019 USD ($)) for school-based deworming across two years
Subtitle: Total economic costs are presented, as well as a breakdown of costs by routine program vs. supporting program activities, and financial vs. opportunity costs

	Benin [a]	India [b]	Malawi [a]
**Planning**	**$ 0.07**	**$ 0.00**	**$ 0.01**
Supporting (financial)	$ 0.07	—	$ 0.01
**Program management**	**$ 0.69**	**$ 0.19**	**$ 0.40**
Routine (financial)	—	—	$ 0.15
Routine (opportunity) – time costs for government staff [c]	$ 0.25	$ 0.11	$ 0.00
Supporting (financial)	$ 0.44	$ 0.08	$ 0.25
**Community sensitization**	**$ 0.26**	**$ 0.01**	**$ 0.11**
Routine (financial)	$ 0.14	$ 0.01	$ 0.04
Routine (opportunity) – time costs for government staff and volunteers	—	—	$ 0.05
Supporting (financial) – additional sensitization activities	$ 0.05	—	$ 0.01
Supporting (financial) – NGO supervision	$ 0.07	—	$ 0.02
**Training costs**	**$ 0.61**	**$ 0.18**	**$ 0.25**
Routine (financial)	$ 0.27	$ 0.02	$ 0.08
Routine (opportunity) – time costs for government staff and volunteers	$ 0.20	$ 0.14	$ 0.11
Supporting (financial) – training of electronic data collectors	$ 0.06	$ 0.02	$ 0.02
Supporting (financial) – NGO supervision and training support	$ 0.08	$ 0.01	$ 0.04
**Drug delivery**	**$ 0.83**	**$ 0.33**	**$ 0.91**
Routine (financial)	$ 0.12	$ 0.01	$ 0.22
Routine (opportunity) – time costs for government staff and volunteers	$ 0.56	$ 0.28	$ 0.17
Routine (opportunity) – donated drugs	$ 0.06	$ 0.01	$ 0.06
Supporting (financial) – electronic data capture	$ 0.02	$ 0.02	$ 0.21
Supporting (financial) – NGO supervision	$ 0.07	$ 0.01	$ 0.25
Total costs [d]	***$ 2.47***	***$ 0.72***	***$ 1.69***
Routine (financial)	*$ 0.53*	*$ 0.03*	*$ 0.48*
Routine (opportunity)	*$ 1.07*	*$ 0.54*	*$ 0.40*
Supporting (financial)	*$ 0.87*	*$ 0.14*	*$ 0.81*

Acronyms: non-governmental organization (NGO)
[a] Analysis includes two years of SBD. In India, SBD was conducted bi-annually, so results are presented as the average across four rounds.
[b] Analysis includes two years of SBD. In Malawi and Benin, SBD was conducted annually, so results are presented as the average of two rounds.
[c] Government staff include supervisory and implementing staff whose salaries are paid by the ministry of health. Examples include: nurses and health officers, teachers, and national and subnational government officials involved in the program.
[d] Routine and supporting activities and related resources are described in Appendix 2. Financial costs represent actual expenditure on goods and services purchased by the government or NGO implementing partner. Opportunity costs, on the other hand, include costs forgone by using a resource in a particular way. These opportunity costs recognize and value the cost of using resources, as these resources are then unavailable for productive use elsewhere. Opportunity costs in this analysis include: costs of donated albendazole, volunteer time spent on the project (such as volunteer drug distributors), and estimated government staff salary costs.

For peer review only

Additional programmatic costs

Costs of additional activities, such as an annual census, prevalence surveys, and coverage surveys are not included within cMDA and SBD unit cost estimates, but are detailed in Appendix 5). In brief, costs of annual censuses ranged from $0.54 (India year 2) to $1.81 (Benin year 1) per person censused. Annual prevalence surveys where stool samples were analyzed using Kato-Katz ranged from $11.98 (India year 1) to $28.78 per person surveyed (India year 2); variability in costs was due to cross-country differences and shared laboratory costs in year 1 of the survey. Lastly, coverage surveys conducted post-MDA were estimated between $1.33 (India year 1) to $4.64 (Benin year 1) per person surveyed.

Sensitivity analyses

In one-way and two-way sensitivity analyses (Figure 2), the largest changes in cMDA and SBD unit costs were driven by altering coverage rates and supportive costs. Changing coverage rates in Malawi resulted in the largest change in estimated unit costs. Estimated deworming program coverage rates varied widely across clusters in Malawi (from 19-74% for SBD and 64-96% for cMDA), resulting in unit costs ranging from $1.26 to $4.91 per SBD treatment administered and $1.93 to $2.87 per cMDA treatment administered. Costs decreased in two-way analyses when supportive costs were removed and coverage rates were reduced to approximately 60% cMDA and SBD coverage (assuming that a reduction in support would result in a reduction in coverage); unit costs decreased by 30% or more in most cases. In these sensitivity analyses, the cost of cMDA and SBD became similar, with a net difference of $0.03 to $0.17. Unit costs did not fluctuate substantially in one-way sensitivity analyses exploring opportunity costs of drugs and volunteer time, and two-way sensitivity analyses exploring coverage and sensitization costs.

DISCUSSION

Costs and resource needs are important pieces of evidence for governments considering updating standards of care and related policies, such as a potential shift from SBD to cMDA. The DeWorm3 project provided a unique platform to assess and compare the costs of two deworming treatment strategies (SBD and cMDA) across heterogenous STH-endemic settings. We found the total unit cost per treatment administered to be higher for cMDA compared to SBD in India and Malawi, and comparable in Benin.

Costs of deworming vary considerably in the literature, depending on treatment strategy, resources accounted for, and perspective. Typically, financial costs for SBD are estimated at less than $0.50 per treatment administered.[5] Our SBD routine financial costs align with these estimates, however, our total economic costs are generally higher, due to the inclusion of planning costs, opportunity costs for teachers and other government staff, and supportive supervision and data collection activities. Fewer STH cMDA costs are available in the literature. A study in Kenya estimated total routine program costs of bi-annual cMDA at $0.48 per treatment administered.[22] These estimates fall between DeWorm3 total routine costs in India ($0.42) and Malawi ($0.78), though are notably lower than routine costs in Benin ($1.13). Our cMDA unit costs are comparable to other studies evaluating economic costs of cMDA for NTDs, such as trachoma costs (estimated at $1.53, excluding costs of antibiotics) and LF costs (ranging from $0.40 to $5.87, including drug costs).[23-25]

This study disaggregates routine program costs from supportive costs that are used to increase coverage (additional sensitization, NGO supervision, and electronic data collection). Total routine costs of cMDA were lower than SBD costs across countries. This is largely driven by salary costs for teachers and school directors who generally spend 1-3 days each year supportive SBD. Similar findings were observed in Niger, where deworming was delivered via SBD to children and via community-based treatment to children and at-risk adults (at fixed locations or their homes); unit costs of SBD were higher at $0.76 compared to $0.46 for community treatment. Differences in costs were attributed to CDDs treating more individuals than teachers.[26] These findings highlight the large impact of fully accounting for costs associated with the delivery workforce, including teachers involved in SBD.

For peer review only - http://bmjopen.bmj.com/site/about/guidelines.xhtml

As it is not possible to disentangle the precise impact of supportive activities on coverage, sensitivity analyses were conducted to explore the potential impact of reducing supportive activities on unit costs. If supportive activities were removed and coverage reduced as a result, unit costs were estimated to drop between 10-50%. Although opportunities for electronic data collection during MDA are increasing (ex. ESPEN Collect), not all programs may choose to proceed with more resource-intensive mobile data collection.[27] However, evidence suggests high coverage of cMDA may be necessary to interrupt transmission, and thus the total costs presented in this study may be representative of costs incurred by elimination programs.

Given the experimental nature of cMDA and the DeWorm3 platform upon which it was implemented, cMDA costs may vary if launched within routine health systems. Depending on existing capacity within countries, governments could see a reduction in costs due to cost-sharing between other community-based or NTD programs. Additionally, studies suggest that MDA costs are subject to economies of scale.[28] Costs of cMDA collected over the first two years of implementation in DeWorm3 may have been high due to start-up costs, and therefore costs could reduce over time with experience, as observed in other cMDA programs.[25] Future modeled analyses of DeWorm3 costing data will explore costs of scaling cMDA programs, altering frequencies and sampling strategies for conducting additional program activities (e.g., censuses, prevalence surveys, and coverage surveys), and examining implications on drug costs if cMDA for STH were to be scaled up widely.

Unit costs of cMDA and SBD varied across sites for several reasons, detailed in Appendix 6. Briefly, sites varied in the degree of NGO involvement. In Benin, there was a strong collaboration between the DeWorm3 team and the Ministry of Health, which incurred more staff allowances and opportunity costs. In India, there was a greater separation of responsibilities, with the DeWorm3 team implementing cMDA and the Government of India implementing SBD. The Malawi DeWorm3 team led the implementation of both cMDA and SBD with supervisory support from the Government of Malawi. Given SBD was solely led by the Government of India, routine financial costs were substantially lower than costs incurred during SBD in Benin and Malawi. A second driver of heterogeneity in SBD costs is variation in school staff involvement across sites. Lastly, the different numbers of treatments administered, due to population sizes and coverage rates, affected unit costs. Previous studies have similarly reported differences in unit costs across countries, and wide intra-country variation. The TUMIKIA study reported an average unit cost of $0.76 USD per person treated for biannual cMDA in Kenya, varying from $0.49—$1.85 across clusters.[10] Additionally, during nationwide scale-up of SBD in Uganda, costs varied $0.41—$0.91 across districts.[11] The inter- and intra-country variations highlight the many ways STH treatment strategies can be implemented, and how community-based health campaigns may need to be adjusted to adapt to specific population needs.

In addition to unit costs, other metrics should be considered to determine the relative value of cMDA and SBD. Cost-effectiveness analyses are important to compare costs to health benefits gained. If more children are treated through cMDA than SBD, and/or overall STH prevalence is reduced, costs per infection-year averted may be lower for cMDA compared to SBD.[22] If cMDA interrupts STH transmission, the long-term reduction in STH burden as a result of cMDA could be substantial. After DeWorm3 trial results are unblinded, further analyses will determine the incremental cost-effectiveness of cMDA compared to SBD under multiple time horizons to account for the long-term benefits of elimination.

There are several limitations to this analysis. As described above, there were different degrees of DeWorm3 involvement in SBD across sites; data sources and some driving assumptions thus necessarily varied. Although DeWorm3 trial costs were excluded from this costing analysis, we anticipate that program management, planning, and supervision costs may be higher than what would be observed routinely. Other assumptions are described in detail in Appendix 2.

CONCLUSION

This study provides evidence from a large micro-costing study, with over 8,000 costing data points over twelve rounds of cMDA and eight rounds of SBD in Benin, India, and Malawi DeWorm3 sites. To our knowledge, this is the first study to directly compare costs of SBD to cMDA for STH programs.[5] On average, cost per treatment administered through cMDA was more expensive than SBD in India and Malawi, and comparable in Benin. The largest difference in sub-activity costs was related to drug delivery, where cMDA financial costs for routine resources (e.g. CDD allowances) and supporting activities (e.g. additional supervision) were notably higher than for SBD across all three countries. Although financial costs were higher for cMDA, opportunity costs for government-funded staff and volunteers were higher for SBD, mainly driven by teacher time. Overall, wages and per-diems represented the largest share of costs across countries and treatment strategies. Program planners should consider what changes in staffing and other resources are needed to implement cMDA at scale, knowing that costs may vary given cross-country differences and economies of scale. Future budget-impact and cost-effective analyses will generate additional evidence on the value for money and affordability of cMDA compared to SBD.

DATA AVAILABILITY STATEMENT

Data collected from this study, including resources used, costs incurred, and the number of albendazole treatments administered, and a corresponding data dictionary will be made available upon reasonable request to the corresponding author. For inquiries, contact Dr. Arianna R. Means at aerubin@uw.edu.

RESEARCH ETHICS STATEMENT

This study was affiliated with a trial that was reviewed and approved by the Institut de Recherche Clinique au Bénin (IRCB) through the National Ethics Committee for Health Research (002-2017/CNERS-MS) from the Ministry of Health in Benin, The London School of Hygiene and Tropical Medicine (12013), The College of Medicine Research Ethics Committee (P.04/17/2161) in Malawi, and the Institutional Review Board at Christian Medical College, Vellore (10392). The DeWorm3 Project was also approved by the University of Washington (STUDY00000180).

ACKNOWLEDGEMENTS

The authors wish to thank all of the study participants, communities and community leaders, national NTD program staff, and other local, regional, and national partners who have participated or supported the DeWorm3 study. We also would like to acknowledge the work of all members of the DeWorm3 study teams and affiliated institutions. We give special thanks to Dr. Kumudha Aruldhas, Monrenike Bada, Miranda Benson, Chikondi Chikotichalera, Dr. Sobana Devavaram, Sean Galagan, Kate Halliday, Noel Joyce Mary Hillari, Hugo Legge, Providence Nindi, Chinnaduraipandi Paulsamy, Emily Pearman, Rajeshkumar Rajendiran, Sarah Smith, Elodie Yard, the Ministry of Health and Family Welfare, Delhi, the Directorate of Public Health, Chennai, and Ministère de la Santé du Bénin for assisting with data collection, cleaning, and interpretation.

AUTHOR CONTRIBUTIONS

KG, MS, KK, AL, MI, RB, RP, SSRA, JLW, and ARM conceptualized the project and methodology; JLW secured the funding. EA, SPK, JS, SPG, WL, and PH collected the data. EA, SPK, JS, SPG, WL, KG, MS, and CM curated the data. KG, MS, and CM analyzed the data. EA and MI were involved in the validation and interpretation of results from Benin; SPK and SPG were involved in validation and interpretation of

For peer review only

results from India; JS, WL, and KK were involved in validation and interpretation of results from Malawi. ARM and CM drafted the manuscript and all authors revised and approved the manuscript for content.

COMPETING INTERESTS

The authors of this study declare no competing interests.

FUNDING

This work was funded by the Bill & Melinda Gates Foundation (INV-022149). The funders had no role in study design, data collection, data analysis, data interpretation, or writing of the article. We received no compensation from pharmaceutical companies or other agencies to write this article.

For peer review only

REFERENCES

1 Pullan RL, Smith JL, Jasrasaria R, et al. Global numbers of infection and disease burden of soil transmitted helminth infections in 2010. *Parasit Vectors* 2014; **7**: 37.

2 World Health Organization. Guideline: Preventive chemotherapy to control soil-transmitted helminth infections in at-risk population groups. Geneva, Switzerland: World Health Organization, 2017 http://www.who.int/nutrition/publications/guidelines/deworming/en/ (accessed Jan 5, 2021).

3 World Health Organization. Ending the neglect to attain the sustainable development goals: a road map for neglected tropical diseases 2021–2030: overview. World Health Organization, 2020 https://apps.who.int/iris/handle/10665/332094.

4 London Declaration on Neglected Tropical Diseases. Uniting to Combat Neglected Tropical Diseases, 2012 https://unitingtocombatntds.org.

5 Turner HC, Truscott JE, Hollingsworth TD, et al. Cost and cost-effectiveness of soil-transmitted helminth treatment programmes: systematic review and research needs. *Parasit Vectors* 2015; **8**. DOI:10.1186/s13071-015-0885-3.

6 Jia T-W, Melville S, Utzinger J, et al. Soil-transmitted helminth reinfection after drug treatment: a systematic review and meta-analysis. *PLoS Negl Trop Dis* 2012; **6**: e1621.

7 Anderson R, Truscott J, Hollingsworth TD. The coverage and frequency of mass drug administration required to eliminate persistent transmission of soil-transmitted helminths. *Philos Trans R Soc B Biol Sci* 2014; **369**: 20130435.

8 Ásbjörnsdóttir KH, Ajjampur SSR, Anderson RM, et al. Assessing the feasibility of interrupting the transmission of soil-transmitted helminths through mass drug administration: The DeWorm3 cluster randomized trial protocol. *PLoS Negl Trop Dis* 2018; **12**: e0006166.

9 Brooker SJ, Nikolay B, Balabanova D, et al. Global feasibility assessment of interrupting the transmission of soil-transmitted helminths: a statistical modelling study. *Lancet Infect Dis* 2015; **15**: 941–50.

10 Pullan RL, Halliday KE, Oswald WE, et al. Effects, equity, and cost of school-based and community-wide treatment strategies for soil-transmitted helminths in Kenya: a cluster-randomised controlled trial. *The Lancet* 2019; **393**: 2039–50.

11 Brooker S, Kabatereine N, Fleming F, et al. Cost and cost-effectiveness of nationwide school-based helminth control in Uganda. *Health Policy Plan* 2008; **23**: 24–35.

12 Adamiak G. Methods for the economic evaluation of health care programmes, 3rd ed. *J Epidemiol Community Health* 2006; **60**: 822–3.

13 Claxton K, Revill P, Sculpher M, et al. The Gates Reference Case for Economic Evaluation. The Bill and Melinda Gates Foundation, 2014.

14 Galactionova K, Sahu M, Gideon S, et al. Costing interventions in the field: preliminary cost estimates and lessons from an evaluation of community-wide Mass Drug Administration for elimination of Soil-Transmitted Helminths in the DeWorm3 trial. *BMJ Open* 2021; **11**: e0409734

15 Means AR, Ajjampur SSR, Bailey R, et al. Evaluating the sustainability, scalability, and replicability of an STH transmission interruption intervention: The DeWorm3 implementation science protocol. *PLoS Negl Trop Dis* 2018; **12**: e0005988.

16 World Bank. Official exchange rate (LCU per US$, period average). https://data.worldbank.org/indicator/PA.NUS.FCRF (accessed Jan 27, 2021).

17 Anna Vassall, Sedona Sweeney, Jim Kahn, et al. Reference Case for Estimating the Costs of Global Health Services and Interventions. Global Health Cost Consortium, 2017 https://ghcosting.org/pages/standards/reference_case (accessed June 16, 2021).

18 GDP deflator (base year varies by country) | Data. https://data.worldbank.org/indicator/NY.GDP.DEFL.ZS (accessed June 16, 2021).

19 Turner HC, Lauer JA, Tran BX, et al. Adjusting for Inflation and Currency Changes Within Health Economic Studies. *Value Health* 2019; **22**: 1026–32.

20 Tamil Nadu State Government Department of Evaluation and Applied Research. Tamil Nadu - An Economic Appraisal 2011-12 to 2013-14. .

21 International Labor Organization. ILOSTAT database. https://ilostat.ilo.org/data (accessed Aug 25, 2021).

22 Turner HC, Bundy DAP. Programmatic implications of the TUMIKIA trial on community-wide treatment for soil-transmitted helminths: further health economic analyses needed before a change in policy. *Parasit Vectors* 2020; **13**: 102.

23 Kolaczinski JH, Robinson E, Finn TP. The Cost of Antibiotic Mass Drug Administration for Trachoma Control in a Remote Area of South Sudan. *PLoS Negl Trop Dis* 2011; **5**: e1362.

24 Goldman AS, Guisinger VH, Aikins M, et al. National Mass Drug Administration Costs for Lymphatic Filariasis Elimination. *PLoS Negl Trop Dis* 2007; **1**. DOI:10.1371/journal.pntd.0000067.

25 Goldman AS, Brady MA, Direny A, et al. Costs of Integrated Mass Drug Administration for Neglected Tropical Diseases in Haiti. *Am J Trop Med Hyg* 2011; **85**: 826–33.

For peer review only

26 Leslie J, Garba A, Oliva EB, et al. Schistosomiais and Soil-Transmitted Helminth Control in Niger: Cost Effectiveness of School Based and Community Distributed Mass Drug Administration. *PLoS Negl Trop Dis* 2011; **5**. DOI:10.1371/journal.pntd.0001326.

27 Expanded Special Project for Elimination of Neglected Tropical Diseases (ESPEN). ESPEN Collect. https://espen.afro.who.int/tools-resources/espen-collect (accessed May 25, 2021).

28 Fitzpatrick C, Fleming FM, Madin-Warburton M, et al. Benchmarking the Cost per Person of Mass Treatment for Selected Neglected Tropical Diseases: An Approach Based on Literature Review and Meta-regression with Web-Based Software Application. *PLoS Negl Trop Dis* 2016; **10**: e0005037.

FIGURES

Figure 1: Flow of DeWorm3 activities conducted in intervention and control clusters.
Activities include: census, prevalence survey, school-based deworming, community-wide mass drug administration, and coverage survey
Footnotes:
Acronyms: quarter (Q), school-based deworming (SBD), community-wide mass drug administration (cMDA).
[a] In India, SBD is also conducted in quarter 2, prior to the coverage survey.
[b] In Malawi, no prevalence survey was conducted in year 2.

Figure 2: One- and two-way sensitivity analyses of unit costs (2019 USD ($)).
a) community-wide mass drug administration (cMDA) costs in Benin; b) school-based deworming (SBD) costs in Benin; c) cMDA costs in Malawi; d) SBD costs in Malawi; e) cMDA costs in India; f) SBD costs in India. Details on how each parameter was varied can be found in Appendix 3.

For peer review only

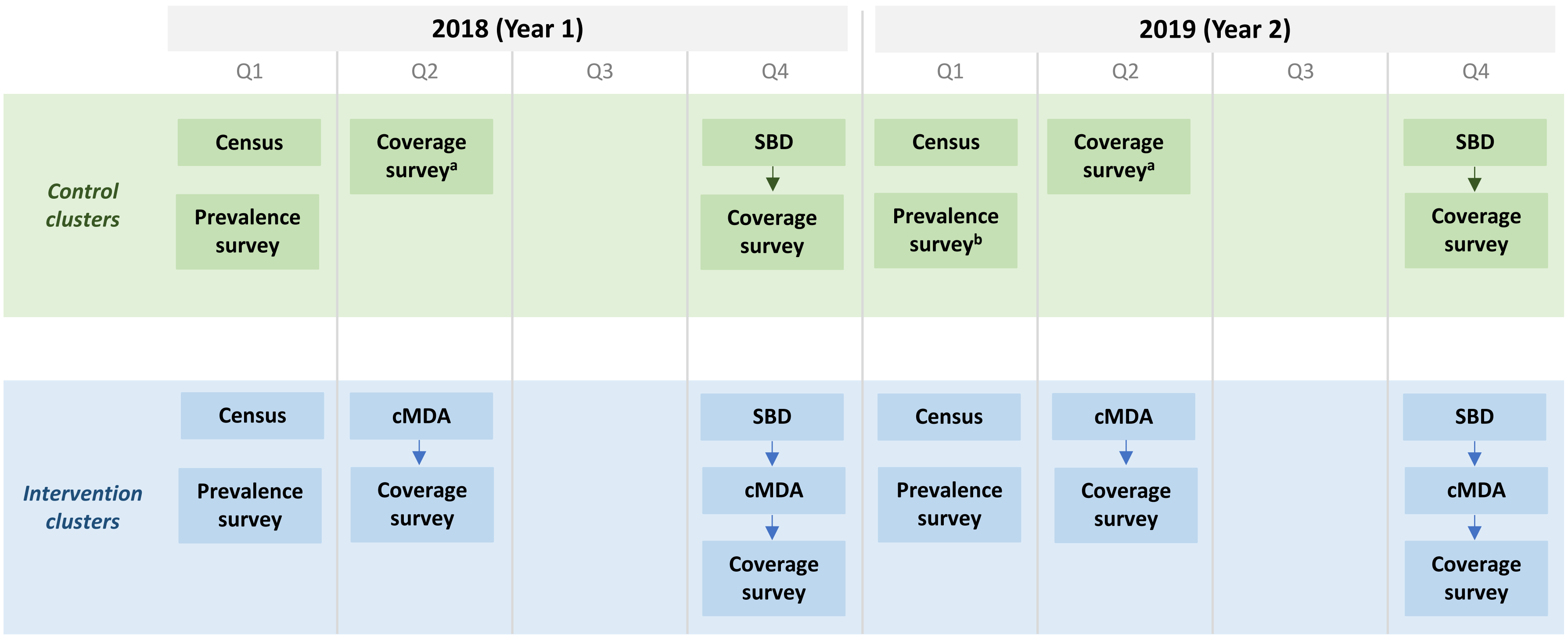
2018 (Year 1)
2019 (Year 2)
Q1
Q2
Q3
Q4
Q1
Q2
Q3
Q4
Control clusters
Census
Prevalence survey
Coverage survey[a]
SBD
Coverage survey
Census
Prevalence survey[b]
Coverage survey[a]
SBD
Coverage survey
Intervention clusters
Census
Prevalence survey
cMDA
Coverage survey
SBD
cMDA
Coverage survey
Census
Prevalence survey
cMDA
Coverage survey
SBD
cMDA
Coverage survey

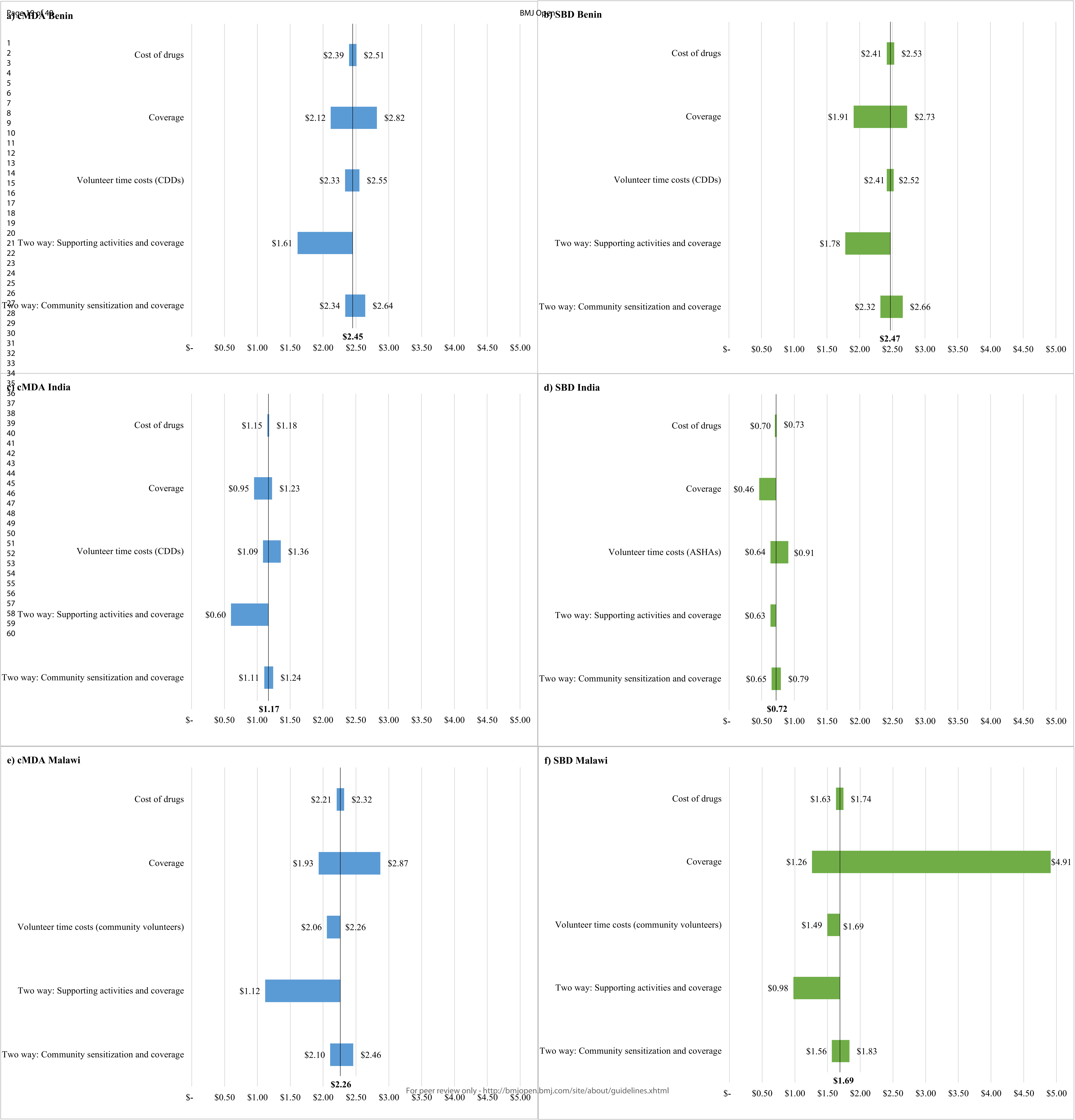
a) cMDA Benin
Cost of drugs
$2.39
$2.51
Coverage
$2.12
$2.82
Volunteer time costs (CDDs)
$2.33
$2.55
Two way: Supporting activities and coverage
$1.61
Two way: Community sensitization and coverage
$2.34
$2.64
$2.45
$- $0.50 $1.00 $1.50 $2.00 $2.50 $3.00 $3.50 $4.00 $4.50 $5.00
b) SBD Benin
Cost of drugs
$2.41
$2.53
Coverage
$1.91
$2.73
Volunteer time costs (CDDs)
$2.41
$2.52
Two way: Supporting activities and coverage
$1.78
Two way: Community sensitization and coverage
$2.32
$2.66
$2.47
$- $0.50 $1.00 $1.50 $2.00 $2.50 $3.00 $3.50 $4.00 $4.50 $5.00
c) cMDA India
Cost of drugs
$1.15
$1.18
Coverage
$0.95
$1.23
Volunteer time costs (CDDs)
$1.09
$1.36
Two way: Supporting activities and coverage
$0.60
Two way: Community sensitization and coverage
$1.11
$1.24
$1.17
$- $0.50 $1.00 $1.50 $2.00 $2.50 $3.00 $3.50 $4.00 $4.50 $5.00
d) SBD India
Cost of drugs
$0.70
$0.73
Coverage
$0.46
Volunteer time costs (ASHAs)
$0.64
$0.91
Two way: Supporting activities and coverage
$0.63
Two way: Community sensitization and coverage
$0.65
$0.79
$0.72
$- $0.50 $1.00 $1.50 $2.00 $2.50 $3.00 $3.50 $4.00 $4.50 $5.00
e) cMDA Malawi
Cost of drugs
$2.21
$2.32
Coverage
$1.93
$2.87
Volunteer time costs (community volunteers)
$2.06
$2.26
Two way: Supporting activities and coverage
$1.12
Two way: Community sensitization and coverage
$2.10
$2.46
$2.26
$- $0.50 $1.00 $1.50 $2.00 $2.50 $3.00 $3.50 $4.00 $4.50 $5.00
f) SBD Malawi
Cost of drugs
$1.63
$1.74
Coverage
$1.26
$4.91
Volunteer time costs (community volunteers)
$1.49
$1.69
Two way: Supporting activities and coverage
$0.98
Two way: Community sensitization and coverage
$1.56
$1.83
$1.69
$- $0.50 $1.00 $1.50 $2.00 $2.50 $3.00 $3.50 $4.00 $4.50 $5.00

APPENDIX

Costs of community-wide mass drug administration and school-based deworming for soil-transmitted helminths: evidence from Benin, India, and Malawi

TABLE OF CONTENTS

Appendix 1: Additional details on DeWorm3 activities implemented 2

Table 1: Implementation characteristics of the DeWorm3 trial at study sites 2

Table 2: Narrative description of DeWorm3 mass drug administration activities 3

Appendix 2: Additional details on costing methodology 6

Table 3: DeWorm3 instruments for cost collection 6

Table 4: Summary of resources included in community-wide mass drug administration and school-based deworming costing analyses, by routine and supporting program costs 7

Table 5: Key costing inputs (non-exhaustive) for community-wide mass drug administration and school-based deworming, per country 9

Table 6: Key assumptions regarding unit cost analysis for community-wide mass drug administration and school-based deworming 11

Appendix 3: Additional details of sensitivity analysis methodology 13

Appendix 4: Costs in local currency 14

Table 7: Total economic costs and number of treatments administered through community-wide mass drug administration and school-based deworming, per country-round, in local currency 14

Table 8: Average unit costs (2019 local currency) for community-wide mass drug administration across two years 15

Table 9: Average unit costs (2019 local currency) for school-based deworming across two years 16

Table 10: Annual costs of additional deworming program activities, including censuses, prevalence surveys, and coverage surveys, across two years of implementation (2019 local currency) 17

Appendix 5: Additional costing results 18

Table 11: Percentage of DeWorm3 activity unit costs that are fixed vs. variable, by activity and country 18

Figure 1: Total financial and opportunity costs of community-wide mass drug administration and school-based deworming by input-classification (including routine and supporting program costs) 19

Table 12: Community-wide mass drug administration costs by input classification, by routine vs. supporting costs 20

Table 13: School-based deworming costs by input classification, by routine vs. supporting costs 20

Figure 2: Share of community-wide mass drug administration costs, by country, sub-activity, and category 21

Figure 3: Box and whisker plots describing variability in median and range of sub-activity costs across four rounds of community-wide mass drug administration in Benin, India, and Malawi; two rounds of school-based deworming in Benin and Malawi; and, four rounds of school-based deworming in India 22

Table 14: Annual costs of additional deworming program activities, including censuses, prevalence surveys and coverage surveys, across two years of implementation (2019 USD ($)) 25

Appendix 6: Description of cost differences across countries 26

Table 15: Drivers of heterogeneity in costs across sites 26

Appendix 7: Appendix references 28

For peer review only - http://bmjopen.bmj.com/site/about/guidelines.xhtml

Appendix 1: Additional details on DeWorm3 activities implemented

In the below table, we provide additional details on how community-wide mass drug administration (cMDA) and school-based deworming (SBD) were implemented in each country.

Table 1: Implementation characteristics of the DeWorm3 trial at study sites

	Benin	India	Malawi
Study location	Come Commune	Tamil Nadu State (Vellore and Thiruvanamalai districts)	Mangochi District
Implementing organizations	• Institut de Recherche Clinique du Benin<br>• Institut de Recherche pour le Développement<br>• Ministry of Health, Benin	• Christian Medical College, Vellore<br>• Ministry of Health and Family Welfare, New Delhi and Directorate of Public Health, Chennai	• Blantyre Institute for Community Outreach<br>• London School of Tropical Medicine and Hygiene<br>• Ministry of Health and Education, Malawi
cMDA strategy (20 intervention clusters)	• Bi-annual cMDA in all ages.<br>• Community drug distributors (volunteers) delivered drugs<br>• Implemented by DeWorm3	• Bi-annual cMDA in all ages; following National Deworming Day (described below)<br>• Community drug distributors (volunteers) delivered drugs<br>• Implemented by DeWorm3	• Bi-annual cMDA in all ages<br>• Health Surveillance Assistants, employed by the government, delivered drugs<br>• Implemented by DeWorm3
SBD strategy (20 intervention clusters and 20 control clusters)	• SBD conducted annually<br>• Treatment of children 5-14 years old<br>• Implemented by the Ministry of Health (with a subcontract from DeWorm3)	• National Deworming Days, conducted bi-annually in schools and Anganwadi centers (pre-schools)<br>• Treatment of children 1-19 years old<br>• Implemented by the Ministry of Health and Family Welfare, New Delhi and Directorate of Public Health, Chennai	• SBD conducted annually, integrated with "Child Health Days"<br>• Community mop-up for non-enrolled children<br>• Treatment of children 1-14 years old<br>• Implemented by DeWorm3
Additional trial activities	• Planning meetings<br>• Annual census<br>• Two prevalence surveys<br>• Bi-annual coverage survey (after each round of MDA	• Planning meetings<br>• Annual census<br>• Two prevalence surveys<br>• Bi-annual coverage survey	• Planning meetings<br>• Annual census<br>• One prevalence survey Bi-annual coverage survey

Acronyms: mass drug administration (MDA), community-wide MDA (cMDA), school-based deworming (SBD)

2

Table 2: Narrative description of DeWorm3 mass drug administration activities

MALAWI		
**Sub-activity**	**Community-wide mass drug administration (cMDA)**	**School-based delivery (SBD)**
Supply chain	• *Shipment to country*: Drugs were donated, ordered through the WHO. One shipment for both cMDA and SBD was made for 1.5 million doses and sent by ship, which supplied all years of the project. The stock was kept at the Central Drug Stores in Lilongwe and then dispensed to the study.<br>• *Storage and disbursement*: Albendazole for each MDA round was stored in the Deworm3 office in Namwera. During cMDA, albendazole was dispensed daily to the enumerators, and the remaining stock was returned to the office each evening. Drug supply was monitored using stock control cards and excel files of stock issued to enumerators.	• *Shipment:* Same shipment as cMDA.<br>• *Storage and disbursement*: Albendazole for each MDA round was stored in the Deworm3 office in Namwera. Field officers (employed by DeWorm3) transported the drugs between the office and schools during SBD.
Sensitization	Several committees and community boards were engaged for MDA sensitization. In year 2, the DeWorm3 team employed additional sensitization measures to improve community engagement and maximize treatment coverage. Activities included:<br>• Area Development Council meetings with group village headmen and/or representatives from Village Development Committees.<br>• Village level community meetings were conducted by Health Surveillance Associates (HSAs) and volunteers.<br>• Village dramas and public announcements (year 2 only).<br>• Religious and Traditional Authority leaders of the Community Advisory Board visited communities that displayed signs of community tension or low participation to resolve any communication issues (year 2 only).	Sensitization for SBD was combined with cMDA sensitization activities.
Training	• *Health staff and volunteers*: Field officers trained HSAs at health centers and halls. Training sessions were one day long, though they were conducted over the course of two days to accommodate all health center staff. Afterward, HSAs oriented volunteers.<br>• *Enumerators:* Trial coordinator and field officers trained enumerators for two days, followed by a three-day pilot of data collection instruments used during MDA.	• *Health staff and volunteers:* Training for SBD was combined with cMDA training activities.<br>• *Enumerators:* Training for SBD was combined with cMDA training activities.<br>• *Teachers and other school staff:* Training of teachers and principal education assistants was conducted by field officers supported by the Ministry of Health STH Programme Manager.
Drug delivery	Drug delivery was conducted twice per year, in intervention clusters only (n=20), by teams of enumerators, HSAs, and volunteers. HSAs were responsible for a relatively large number of households. HSAs supervised volunteers (about 4 volunteers per HSA). Enumerators were driven daily from Namwera to the community with their drug stocks, and HSAs were picked up along the way. Area Development Council members helped in mobilizing the community on the day of MDA.	School-based deworming was conducted once per year in all DeWorm3 clusters (n=40); in intervention clusters, SBD was conducted prior to cMDA. Treatment was administered at each school by the link HSA, with administrative support from two school teachers and the headteacher.<br><br>Children were also treated for schistosomiasis, using praziquantel. Costs of praziquantel were excluded from this costing analysis.
Supervision	Supervision was conducted by the DeWorm3 trial coordinator, DeWorm3 field officers, local health officers (Environmental Health Officers, Assistant Environmental Health Officers, District Environmental Health Officer, District Health Officer), District Council Representative, District STH Coordinator, and the Ministry of Health STH Programme Manager.	Supervision was conducted by the DeWorm3 trial coordinator, DeWorm3 field officers, local health officers (Environmental Health Officers, Assistant Environmental Health Officers, District Health Officer), District Council Representative, District STH Coordinator, Primary Education Authorities, and a representative from the Ministry of Education.
Mop-up	Malawi did not have a distinct mop-up period for cMDA. Instead, progress on coverage was tracked by a DeWorm3 monitoring dashboard, informed by electronic data collection forms. MDA was only considered complete once the dashboard indicated that all households had been treated or visited three times; all individuals who were absent from the household, but not migrated, at the first visit were followed up at least two further times.<br><br>Mop-up costs were estimated in the analysis as approximately 1-2 days of work, to indicate the individuals who were followed up with more than once.	Village level MDA of children who weren't in school was conducted as "mop-up" for two days after SBD.

For peer review only

INDIA		
**Sub-activity**	**Community-wide mass drug administration (cMDA)**	**School-based delivery (SBD)**
Supply chain	• *Shipment to country*: Drugs were donated, ordered through the WHO. Drugs were ordered centrally by the Ministry of Health and Family Welfare, through the national NTD program.<br>• *Storage and disbursement*: Consignment was brought to the central DeWorm3 office in Vellore, and delivered to two subsite field offices. Field supervisors managed the tablets and provided them to fieldworkers daily to take to the villages for community drug distributors (CDDs) to dispense. The remaining tablets were returned to the office at end of the day.	• *Shipment to country*: Same shipment as cMDA.<br>• *Storage and disbursement*: Consignment was brought to the central DeWorm3 office in Vellore, and delivered to two subsite field offices. Field supervisors managed the tablets and provided them to Village Head Nurses to supply all schools and Anganwadi Centers.
Sensitization	• National Deworming Day sensitization materials were adapted to include information on cMDA; 1000 posters and 200 banners were posted in villages.<br>• Community sensitization meetings were conducted by DeWorm3 field staff using locally designed flipbooks to explain how STH are transmitted and what activities would be undertaken during cMDA.	Cloth banners provided by the government were put up by school staff outside schools and Anganwadi Centers one day before SBD.
Training	• *Health staff and volunteers*: CDDs had a half-day training, conducted by the DeWorm3 medical officer.<br>• *Enumerators:* DeWorm3 fieldworkers were trained by the DeWorm3 trial coordinator and data manager, followed by a short pilot period to test forms used during MDA.	• *Health staff and volunteers:* Training for SBD was combined with cMDA training activities.<br>• *Enumerators:* Training for SBD was combined with cMDA training activities.<br>• *Teachers and other school staff:* Workshops were held for teachers, Anganwadi Workers, and Village Health Nurses at every primary health center, conducted by respective primary health center medical officers.
Drug delivery	Drug delivery was conducted twice per year, in intervention clusters only (n=20), by teams of DeWorm3 fieldworkers (serving as enumerators) and CDDs, who walked door to door in the community. Nurses and medical officers supported with adverse events.	School-based deworming (called National Deworming Day) was conducted twice per year in all DeWorm3 clusters (n=40); in intervention clusters, SBD was conducted prior to cMDA. Drugs were delivered by Village Health Nurses in schools and Anganwadi Centers. ASHA workers and volunteers provided support as needed. DeWorm3 fieldworkers attended to deliver ink pens and treatment summary sheets. Nurses and medical officers helped with adverse events.
Supervision	Supervision was conducted by DeWorm3 field supervisors, DeWorm3 field managers, and local health workers (Village Head Nurses, Sector Health Nurses, and Community Health Nurses, and Block Medical Officers).	Supervision was conducted by local health workers (Village Head Nurses, Sector Health Nurses, and Community Health Nurses, and Block Medical Officers), central and sub-national level government health authorities.
Mop-up	After cMDA, a mop-up campaign was conducted for 1-4 days to reach absent individuals. Homes with absent individuals were visited up to three times.	One additional day of mop-up was conducted for children who were absent at school on National Deworming Day.

For peer review only - http://bmjopen.bmj.com/site/about/guidelines.xhtml

BENIN		
Sub-activity	**Community-wide mass drug administration (cMDA)**	**School-based delivery (SBD)**
Supply chain	• *Shipment to country*: Drugs were donated, ordered through the WHO. Drugs were ordered centrally by the Ministry of Health, for routine use, and stored in the national storage facility.<br>• *Storage and disbursement:* Drugs were dispatched to the zonal referral hospital in Come, by the National Communicable Disease Control Program (Programme National de Lutte contre les Maladies Transmissibles or PNLMT). Afterward, drugs were transferred to each health center affiliated with DeWorm3, with transit supervised by head doctors at commune level. Nurses collected drugs for the MDA campaign from the referral hospital after training. Nurses then dispensed drugs to CDDs for cMDA. After cMDA, the remaining drugs were transported from clusters to the central level.	• *Shipment to country*: Same shipment as cMDA.<br>• *Storage and disbursement*: Same disbursement process as cMDA, except nurses dispensed drugs to school headmasters rather than to CDDs.
Sensitization	• Information sessions were held with local authorities (town hall), leaders of opinion, religious leaders, professional associations, and town criers.<br>• Messages were passed to the community through town criers, radio broadcasts, specific groups (i.e. women's associations), and religious centers.<br>• Banners and posters were also placed in the community.	• Sensitization for SBD was combined with cMDA sensitization activities.<br>• Additional activities included: a meeting with the chief of the pedagogical region and his advisors (Ministry of Education responsible for Come commune), sensitization of teachers via meetings (year 2), and flier distribution in schools.
Training	• *Health staff and volunteers:* Ministry of Health staff trained 10 head health personnel (health center nurses, Chief Medical Officer, and District Medical Coordinator). Head nurses then trained CDDs. Supervision of training was done by PNLMT technical staff, doctors, and some district and departmental level staff.<br>• *Enumerators:* DeWorm3 staff trained enumerators and controllers (supervisors of enumerators).	• *Health staff and volunteers:* Training was combined with cMDA.<br>• *Teachers and school staff:* Ministry of Education officials and school headmasters were trained by 4 PNLMT staff and 2 DeWorm3 staff.
Drug distribution	Drug delivery was conducted twice per year, in intervention clusters only (n=20). Drugs were distributed by CDDs, joined by an enumerator, with the assumption that each CDD/enumerator pair would treat 60 people per day.	School-based deworming was conducted once per year in all DeWorm3 clusters (n=40); in intervention clusters, SBD was conducted prior to cMDA. Teachers administered drugs to children attending school. School directors/headmasters supervised and reported. CDDs treated non-enrolled children, who were invited to go to the closest school. Enumerators observed and fill out a treatment register.
Supervision	Supervision was conducted by DeWorm3 staff, central PNLMT staff, departmental staff, District Chief Doctors, and sub-district health center nurses.	The same supervisory staff as cMDA.
Mop-up	Two days of mop-up was conducted as needed. There was no mop-up in round 1 of cMDA. In round 4, flooding interrupted cMDA, and extensive mop-up was conducted.	No mop-up period.

Acronyms: World Health Organization (WHO), mass drug administration (MDA), community-wide mass drug administration (cMDA), school-based deworming (SBD), soil-transmitted helminths (STH), neglected tropical diseases (NTD), Programme National de Lutte contre les Maladies Transmissibles (PNLMT).

For peer review only - http://bmjopen.bmj.com/site/about/guidelines.xhtml

Appendix 2: Additional details on costing methodology

In the following tables, we provide additional details on the DeWorm3 costing methodology, including details on data collection tools and key model assumptions.

Table 3: DeWorm3 instruments for cost collection

Source	Primary use	Type of cost	Content
DeWorm3 costing tool	Estimate resource use and costs of activities implemented by the DeWorm3 team	Financial and opportunity	Resource line items, corresponding prices, quantities, and expenditure recorded by sub-activity; separate modules for start-up and implementation
Activity table	Understand the purpose of resource use and how costs from the DeWorm3 costing tool relate to the implementation of activities	Financial and opportunity	Description of operational activities and sub-activities, number of project staff and other resources used, number of days
Activity calendar	Allocate shared costs to activities based on time spent on activities, such as staff salaries	N/A	Start, end dates, and duration of operational activities
Ministry of health costing tools	Estimate government costs of school-based deworming in DeWorm3 study area and Ministry of Health involvement in cMDA	Financial and opportunity	Budgets for routine school-based deworming at the national or state level across countries, government-funded employee salaries, and time spent on activities
MDA forms (i.e. digital treatment forms)	Estimate the number of persons treated, and time spent delivering treatments, to determine time spent by CDDs in each household	Opportunity costs, cost per person treated	Time spent per household to deliver community MDA; the number of persons treated
Census	Calculate relative DeWorm3 population size to district or state, in order to allocate district or state level costs to study area	N/A	DeWorm3 population size, control (SBD) and intervention (cMDA) cluster population size and demographic indicators such as age
School survey	Estimate teacher-related costs	Opportunity	Number of teachers trained, number of teachers involved in SBD, and time spent on activities
Literature	Collect relevant information where gaps persist	Financial and opportunity	District and state population sizes, number of schools per district/state, costs of equipment already owned

Acronyms: community-wide mass drug administration (cMDA), mass drug administration (MDA), school-based deworming (SBD), community drug distributor (CDD)

only

For peer review only - http://bmjopen.bmj.com/site/about/guidelines.xhtml

Table 4: Summary of resources included in community-wide mass drug administration and school-based deworming costing analyses, by routine and supporting program costs

	Routine program costs	Supporting program costs
**Planning**		
Definition	None.	Start-up planning costs for DeWorm3, including developing IEC materials, mobile data collection forms, recruitment, and planning meetings with stakeholders.
Financial costs	None.	DeWorm3 salaries; travel, per-diems, and materials for planning meetings.
**Program management**		
Definition	Estimated operating costs to conduct routine program activities.	Estimated operating costs to conduct supporting program activities such as additional supervision and electronic data collection.
Financial costs	Salaries and overheads for DeWorm3 staff managing the project, including planning and reporting, building rent and utilities, equipment such as computers, vehicles, etc. Borrowed or pre-owned items, annualized across useful life years.	Same as routine program costs.
Opportunity costs	Time costs for government staff involved in the management of deworming programs.	None.
**Community sensitization**		
Definition	Sensitization activities varied across sites and also varied between school-based deworming and community-wide mass drug administration. For a complete list of activities conducted in each country, please see appendix table 2. Examples include meetings with local committees/authorities/leaders, engagement with village chiefs, village dramas, door-to-door sensitization, posters and banners, radio advertisements, public criers.	Activities beyond those expected in routine programs, such as sensitizing the community to DeWorm3 research activities.
Financial costs	Per-diems and travel allowances, meeting costs such as refreshments and chair rentals, sensitization materials.	Examples include meeting costs for a Community Advisory Board, resources to hold a soccer competition/community event, and additional teacher sensitization.
Opportunity costs	Time costs for government-funded staff involved in sensitization (Health Surveillance Associates). Uncompensated time for volunteer staff who were involved in sensitization, such as community drug distributors. Time is valued using average national or regional salaries.	None.
**Training**		
Definition	Resources to train community drug distributors, volunteers, and health workers involved in drug delivery.	Resources to train enumerators involved in electronic data collection, as well as additional supervision by deworm3 implementing partners.
Financial costs	Per-diems, printed materials, refreshments, and hall rental.	Per-diems, printed materials, refreshments, and hall rental.

For peer review only - http://bmjopen.bmj.com/site/about/guidelines.xhtml

Opportunity costs	Time costs for government-funded staff involved in training (e.g., teachers, supervisors). Uncompensated time for volunteer staff who were trained, such as community drug distributors (Benin and India), ASHAs (India), and volunteers (Malawi). Time is valued using average national or regional salaries.	None.
**Drug delivery**		
Definition	Resources to deliver drugs either in the community or at schools, including mop-up.	Additional resources for enumerators to collect electronic monitoring data, and for supervision by deworm3 implementing partners.
Financial costs	Fuel, car rentals and per-diems for government supervisors, allowances/incentives for drug distributors, drugs for adverse events. Time for government supervisors, teachers.	Per-diems, mobile allowances for uploading data, fuel, and car hires.
Opportunity costs	Time costs for government-funded staff involved in drug delivery (e.g., teachers, supervisors). Uncompensated time for volunteer staff, such as community drug distributors (Benin and India), ASHAs (India), and volunteers (Malawi). Time is valued using average national or regional salaries. Costs of donated drugs.	None.

For peer review only

For peer review only - http://bmjopen.bmj.com/site/about/guidelines.xhtml

Table 5: Key costing inputs (non-exhaustive) for community-wide mass drug administration and school-based deworming, per country

	Benin	India	Malawi	Data source
**Days of drug delivery, including mop-up**				
cMDA: mean days (min-max)	12 (11—15)	13 (11—16)	16 (16)	DeWorm3 activity list
SBD: mean days (min-max)	2 (2)	2 (2)	5 (5)	DeWorm3 activity list
**Drug costs**				
Albendazole: opportunity cost, per tablet	$0.05	$0.01	$0.05	Benin and Malawi: GSK[1], India: National Deworming Day financial guidelines[b]
**Drug Distributors (CDDs and HSAs)**				
Staff involved: mean (min-max)	90 (90)	127 (114—164)	56 (56)	DeWorm3 costing tool
Monthly salary: approximate	$125	$126	$203	Benin: ILO[2]; India: State salary estimates[3], Malawi: DeWorm3 Ministry of Health costing survey
Time spent on cMDA training and sensitization: days	2	2	2	DeWorm3 costing tool
Time spent per cMDA visit, including travel: median minutes	17	11	14	DeWorm3 MDA forms
Number of cMDA visits conducted per drug distributor, per round: median visits	181	177	328	DeWorm3 MDA forms
Daily allowances[a] for drug delivery	$3.41	$3.55	$5.37	DeWorm3 costing tool
**Teachers**				
Number of schools: median (min-max)	55 (54—55)	254 (228—298)	35 (29—40)	SBD forms
Staff involved in SBD: mean (min-max)	304 (288—320)	339 (331—347)	147 (121—173)	DeWorm3 costing tool and school survey
Monthly salary: approximate[c]	$380	$456	$203	DeWorm3 Ministry of Health costing survey
Time spent on training and reporting: median days	0.25	0.625	0.5	Ministry of Health costing tool
Time spent on drug delivery: median days	0.33	0.25	1	DeWorm3 school survey
Allowances given	None	Per training	Per training, per day of drug delivery	Ministry of Health costing tool, DeWorm3 costing tool
Allowance rate	—	$1.42	$5.41	DeWorm3 costing tool
**Other school staff**				
Position	School Directors	Anganwadi Workers	—	DeWorm3 costing tool and school survey
Number staff involved in SBD: mean (min-max)	55 (54—55)	126 (124—127)	—	DeWorm3 costing tool and school survey
Monthly salary: approximate	$539	$188	—	DeWorm3 Ministry of Health costing survey
Time spent on training and reporting: median days	1.5	0.625	—	Ministry of Health costing tool
Time spent on drug delivery: median days	2	0.25	—	DeWorm3 school survey

Allowances given	Per training	Per training	—	Ministry of Health costing tool, DeWorm3 costing tool
Allowance rate	$17.01	$1.42	—	DeWorm3 costing tool
**DeWorm3 Enumerators**				
Number staff involved: mean (range)	90 (90)	84 (73—107)	57 (50—65)	DeWorm3 costing tool
Daily compensation and allowances	$8.55	$6.50	$14—$34[d]	DeWorm3 costing tool
**DeWorm3 Field Supervisors (Controllers, Field Supervisors, Field Officers)**				
Number staff involved	10	13	4	DeWorm3 costing tool
Daily compensation and allowances: approximate	$21	$12	$20	DeWorm3 costing tool
**DeWorm3 Vehicle Costs**				
Project vehicles	2	1	5	DeWorm3 costing tool
Make of vehicles	Nissan 4x, 5-seater	Mahindra Thar CRDe	Land cruiser 4.2 Diesel 13-seater	DeWorm3 costing tool
Net cost	$37,807	$13,755	$41,137	DeWorm3 costing tool
Useful life years assumed	9	9	9	WHO CHOICE
**DeWorm3 Central Personnel**				
DeWorm3 central key program staff (providing program management and higher level supervision) involved	11	11	8	DeWorm3 costing tool
DeWorm3 central support staff (drivers, accountants, etc.) involved	10	7	10	DeWorm3 costing tool

Acronyms: community-wide mass drug administration (cMDA), school-based deworming (SBD), Gross Domestic Product (GDP), National Deworming Day (NDD), GlaxoSmithKline (GSK)
[a] Type of allowance varied per country (i.e., lunch allowance, mobile data, travel allowance, etc.). Given the travel nature of the work, and the descriptions of these costs, we have chosen to present these costs as allowances rather than compensation for work done. In some countries, the allowances vary based on number of days involved or number of persons reached.
[b] GlaxoSmithKline (GSK) is currently donating albendazole for lymphatic filariasis and soil-transmitted helminth control. The estimated opportunity costs of donated albendazole is $0.45 per tablet. We have also included the cost of shipping, raising the total estimated costs to $0.047. Costs per tablet administered also include 10% wastage, bringing the total to 0.052. Although GSK-donated albendazole was used in the DeWorm3 project, this analysis used the estimated costs of locally procured albendazole in India, as is routinely used in National Deworming Days. Estimated cost per tablet of locally procured albendazole was acquired from the Tamil Nadu State Budget for National Deworming Day.
[c] Salary varies based on level of school.
[d] Some nurses functioned as enumerators were paid a higher rate.

For peer review only - http://bmjopen.bmj.com/site/about/guidelines.xhtml

Table 6: Key assumptions regarding unit cost analysis for community-wide mass drug administration and school-based deworming

Type of cost	Description of costs	Assumptions	Analysis decisions
Trial/research costs	Costs related to conducting the trial component of DeWorm3, such as trial insurance, developing IRB materials, etc.	Trial-related costs exclusively related to research did not affect MDA coverage.	Trial-related costs were excluded from cMDA and SBD unit cost analyses.
Planning costs	Activities related to starting up the trial such as micro-planning, recruitment, procurement, trial sensitization meetings, and development of IEC and training materials.	Planning was relevant to all field activities (census, prevalence survey, cMDA, SBD, and coverage survey).	Planning costs were annualized over 3 years of program implementation and split across activities based on the number of days activities were implemented. When monthly or annual costs needed to be split by days, we assumed 20.5 workdays per month.
Program management costs	Program management costs were fixed costs and included large capital items, rent, and salaried project staff. Program management resources were used in multiple trial activities, (generally) purchased/ employed/ rented/ donated in the planning stages of the trial, and were retained for the duration of the trial.	Program management was relevant to all field activities (Census, prevalence survey, cMDA, SBD, and coverage survey).<br><br>There may have been inefficiencies in resource use. For example, a vehicle that was purchased by DeWorm3 may not be driven every day.<br><br>Some materials that were already owned by the DeWorm3 team would need to be purchased by future implementing organizations.	Capital items were annualized over their useful life years, with a 3% discount rate.<br><br>Costs were split among annual activities based on the number of days spent on each activity. When monthly or annual costs needed to be split by days, we assumed 20.5 workdays per month.<br><br>When costs were shared among multiple programs within the implementing institution, we allocated a percentage of costs towards DeWorm3 (i.e. only a portion of total rent costs for an implementing organization were allocated to DeWorm3, if the organization had multiple grants/projects). When resources were used only by the DeWorm3 project, we assumed full costs of resources, even if not used at full capacity.<br><br>Resources that were already owned by the DeWorm3 team (i.e., vehicles, computers, etc.) were categorized as financial costs in this analysis.
Census costs	All costs to run an annual census conducted prior to MDA in all 40 clusters.	Censuses did not affect MDA coverage.	Census costs were excluded from the cMDA and SBD unit cost analysis.
Prevalence survey costs	An annual prevalence survey was used to assess STH prevalence across the 40 clusters.	In year 1, a longitudinal monitoring cohort (LMC) of approximately 6,000 persons was conducted, in addition to a cross-sectional survey of 20,000 persons, per country. In year 2, only the longitudinal monitoring cohort was conducted in Benin and India (no prevalence surveys were conducted in Malawi year 2). It is therefore assumed that approximately 1/4 of shared prevalence survey costs were relevant to the LMC, and 3/4 to the cross-sectional survey, in year 1.<br><br>Prevalence surveys did not affect MDA coverage.	We have presented only the costs of the LMC in this manuscript. Approximately ¼ of shared prevalence survey costs in year 1 were allocated to the LMC.<br><br>Prevalence survey costs were excluded from the cMDA and SBD unit cost analysis and were presented separately.
Coverage surveys	All costs related to conducting post-MDA coverage surveys: conducted after each round of cMDA, sampling approximately 8,000 individuals from the 40 clusters.	Coverage surveys did not affect cMDA coverage.	Coverage survey costs were excluded from the cMDA and SBD unit cost analysis and were presented separately.
DeWorm3 vehicle costs	DeWorm3 project vehicles and related costs (fuel, maintenance, etc), as well as hired vehicles.	Project and hired vehicles were used for additional supervision by DeWorm3 field staff and enumerator transport.	DeWorm3 project vehicles and hired vehicles used in cMDA and SBD were designated as "supporting" costs unless specified as a routine cost (i.e., vehicle hired for government supervisor, fuel reimbursement for training participant, etc.).

Shared MDA (cMDA/SBD) costs	Resources or costs that were described as shared between cMDA and SBD.	In rounds where cMDA was implemented directly after SBD, many sensitization activities were relevant to both cMDA and SBD.<br><br>In rounds where cMDA was implemented directly after SBD, most training activities were relevant to both cMDA and SBD.	Shared costs were split between cMDA and SBD proportionally based on the number of days of each activity (for example, for training costs), or by population treated (for example, for side effects medication).
Input classification for per-diems and allowances	Costs that were described as per-diems or allowances to implementers, trainers, supervisors, or community members.	Unless specified that costs were incentives or compensation, allowances and per-diems were assumed to be used for their designated purpose (for example, lunch allowances used to purchase lunch, travel allowances used for transport).	Per-diems and allowances that were specified as transport allowances, were assigned “vehicles and overheads” as the input classification.<br><br>Per-diems and allowances that were not specified as transport allowances, were assigned “wages and per-diems” as the input classification.<br><br>Unless specified that costs were incentives or compensation, allowances and per-diems were not considered compensation and were not subtracted from estimated opportunity costs. For example, if CDDs were provided a lunch or travel allowance during fieldwork, this was not considered compensation for work done.

Acronyms: community-wide mass drug administration (cMDA), mass drug administration (MDA), school-based deworming (SBD), community drug distributor (CDD), longitudinal monitoring cohort (LMC).

For peer review only

Appendix 3: Additional details of sensitivity analysis methodology

One-way sensitivity analyses: In one-way sensitivity analyses, opportunity costs of drugs, opportunity costs of volunteer time, and coverage rates were explored.

Opportunity costs for albendazole in the costing analysis were valued using the estimated valuation of donated albendazole from GlaxoSmithKline (GSK) plus estimated shipping costs in Malawi and Benin, and the market price of locally procured albendazole in Tamil Nadu, India.[4] To date, GSK has committed to donating albendazole to combat STH until 2025.[5] After 2025, the cost of albendazole to STH programs is unknown. In sensitivity analyses, costs of albendazole were explored by removing opportunity costs as the low input (to explore financial costs to governments during albendazole donation programs) and doubling the opportunity costs of albendazole as the high input (doubling the global valuation of donated albendazole and doubling the India market price to explore how increases in albendazole costs could affect unit costs).

Opportunity costs for volunteers' time in the costing analysis were valued using national (Benin, Malawi) and subnational (India) average wage rates acquired from labor surveys.[6-7] In sensitivity analyses, volunteer time costs were altered by removing opportunity costs for the low input (with the assumption that lunch and travel allowances were sufficient forms of compensation). For the high input, opportunity costs for community volunteers who played a health-delivery role were valued using the estimated salaries of an equivalent health worker.[8]

Total treatments administered per country-round were used in the costing analysis. In sensitivity analyses, coverage rates (and therefore total treatments administered) were altered by applying the highest and lowest observed cluster coverage in a given country to the eligible population for treatment, demonstrating the observed ranges in coverage possible in a given location.

Two-way sensitivity analyses: Two-way sensitivity analyses were also conducted to determine the influence of reductions in supporting activities or sensitization activities alongside reductions in coverage.

The DeWorm3 Project prioritized high coverage of cMDA, intending to reach 90% coverage in each cluster.[9] To do so, the project employed additional supervision and electronic data collection to track coverage in real-time (e.g. "supportive activities"), and respond with mop-up in low coverage areas. These additional activities were resource-intensive, and may not be included in future routine programs. However, removing these additional activities may affect program coverage. In sensitivity analyses, we have explored a two-way analysis where cMDA routine costs are removed, and cMDA coverage rates were reduced by 30% to align more closely with historic deworming coverage rates.[3] Additionally, although SBD is routinely implemented by the governments of India, Benin, and Malawi, the interventions were altered to different extents for delivery during DeWorm3. For example, in Malawi, SBD was implemented through the DeWorm3 project team, rather than via the government of Malawi, leading to different program management costs. In Benin, the government implemented SBD, though the DeWorm3 team provided additional support in the form of supervision and additional sensitization. In sensitivity analyses, SBD coverage was reduced 10% alongside the removal of supportive activities, to reflect how these supportive activities might be increasing coverage during the trial. The relationship between supporting activity costs and coverage rates has not been validated, and future analyses may explore additional changes to input values.

To reach a goal of 90% coverage in each cluster, the DeWorm3 project implemented multiple community sensitization efforts that may have gone above and beyond activities implemented by the government. In two-way sensitivity analyses, the relationship between sensitization costs and coverage rates was explored. For the high-input: sensitization costs were increased 30% with an increase of 10% in coverage rates (not exceeding 100% coverage of eligible populations). For the low-input, sensitization costs were decreased 30% with a decrease of 10% in coverage rates. The relationship between sensitization costs and coverage rates has not been validated, and future analyses may explore additional changes to input values.

Future directions for sensitivity analyses: Given the many costing resources that were included in this analysis, there are endless possibilities of costs that could be altered in sensitivity analyses. Decisions regarding which sensitivity analyses to conduct in this study were based upon field team and expert input regarding influential factors, and differences in implementation across DeWorm3 sites. Future discussions with government stakeholders may provide opportunities to explore how costs may vary in scaled-up programs (e.g. specific allowances for CDDs, frequency and resources needed for training, days of MDA, etc.) allowing for tailored sensitivity analyses.

For peer review only

Appendix 4: Costs in local currency

In the following tables, we present key costing data from the manuscript, presented in local currency. Costs are presented in 2019 West African Francs (XOF) for Benin, 2019 Indian Rupees (INR) for India, and 2019 Malawian Kwacha (MWK) for Malawi.

Table 7: Total economic costs and number of treatments administered through community-wide mass drug administration and school-based deworming, per country-round, in local currency

Metric	Benin (XOF)		India (INR)		Malawi (MWK)	
	cMDA	SBD	cMDA	SBD	cMDA	SBD
Number of treatments administered[a]						
Round 1	45,280	–	55,953	15,266	49,518	–
Round 2	37,913	9,298	55,758	19,152	38,641	16,077
Round 3	42,398	–	57,353	21,396	52,122	–
Round 4	32,529	10,343	57,398	20,586	49,709	12,964
Total costs[b]						
Round 1	61,148,760	–	5,068,089	975,635	94,544,024	–
Round 2	47,069,592	12,912,370	4,536,205	992,149	71,212,140	16,716,181
Round 3	57,116,293	–	4,656,850	900,980	70,936,162	–
Round 4	54,342,916	14,882,855	4,352,396	898,775	72,724,580	17,999,058
Cost per treatment administered						
Round 1	1,350	–	91	64	1,909	–
Round 2	1,242	1,389	81	52	1,843	1,040
Round 3	1,347	–	81	42	1,361	–
Round 4	1,671	1,439	76	44	1,463	1,388

Acronyms: community-wide mass drug administration (cMDA), school-based deworming (SBD)
[a] Treatments administered for cMDA includes all eligible individuals who received treatment by DeWorm3 through cMDA in the intervention clusters (Source: DeWorm3 MDA treatment logs). Population treated for SBD includes all children treated in schools within the DeWorm3 control clusters (Source: SBD treatment logs).
[b] Total costs include both financial and opportunity costs.

For peer review only

Table 8: Average unit costs (2019 local currency) for community-wide mass drug administration across two years
Total economic costs are presented, as well as a breakdown of costs by routine vs. supporting activities, and financial vs. opportunity costs

	Benin (XOF)[a]	India (INR)[a]	Malawi (MWK)[a]
**Planning**	**61**	**3**	**5**
Supporting (financial)	61	3	5
**Program management**	**371**	**28**	**376**
Routine (financial)	164	11	113
Routine (opportunity) – time costs for government staff[b]	6		0
Supporting (financial)	200	17	263
**Community sensitization**	**143**	**12**	**125**
Routine (financial)	64	1	46
Routine (opportunity) – time costs for government staff and volunteers	8	0	27
Supporting (financial) – additional sensitization activities	4	0	10
Supporting (financial) – NGO supervision	66	10	42
**Training costs**	**196**	**8**	**190**
Routine (financial)	70	0	49
Routine (opportunity) – time costs for government staff and volunteers	14	2	18
Supporting (financial) – training of electronic data collectors	63	4	41
Supporting (financial) – NGO supervision and training support	50	1	82
**Drug delivery**	**631**	**32**	**948**
Routine (financial)	210	5	152
Routine (opportunity) – time costs for government staff and volunteers	90	8	136
Routine (opportunity) – donated drugs	0	1	0
Supporting (financial) – electronic data capture	172	13	228
Supporting (financial) – NGO supervision	159	5	433
***Total costs*[c]**	***1402***	***82***	***1644***
Routine (financial)	*509*	*18*	*360*
Routine (opportunity)	*118*	*11*	*181*
Supporting (financial)	*776*	*53*	*1103*

Acronyms: non-governmental organization (NGO)
[a] Analysis includes two years of cMDA. As cMDA was conducted bi-annually in each country, results are presented as the average across four rounds.
[b] Government staff include supervisory and implementing staff whose salaries are paid by the Ministry of Health. Examples include nurses and health officers, HSAs (Malawi only), as well as national and subnational government officials involved in the program.
[c] Routine and supporting activities and related resources are described in Appendix Table 4. Financial costs represent actual expenditure on goods and services purchased by the government or NGO implementing partner. Opportunity costs, on the other hand, include costs forgone by using a resource in a particular way. These opportunity costs recognize and value the cost of using resources, as these resources are then unavailable for productive use elsewhere. Opportunity costs in this analysis include costs of donated albendazole, volunteer time spent on the project (such as volunteer drug distributors), and estimated government staff salary costs.

For peer review only - http://bmjopen.bmj.com/site/about/guidelines.xhtml

Table 9: Average unit costs (2019 local currency) for school-based deworming across two years
Total economic costs are presented, as well as a breakdown of costs by routine program vs. supporting program activities, and financial vs. opportunity costs

	Benin (XOF)[a]	India (INR)[b]	Malawi (MWK)[a]
**Planning**	**43**	**0**	**4**
Supporting (financial)	43	0	4
**Program management**	**406**	**14**	**299**
Routine (financial)	–	–	110
Routine (opportunity) – time costs for government staff[c]	146	8	1
Supporting (financial)	260	6	187
**Community sensitization**	**153**	**0**	**89**
Routine (financial)	83	0	29
Routine (opportunity) – time costs for government staff and volunteers	–	–	39
Supporting (financial) – additional sensitization activities	27	–	5
Supporting (financial) – NGO supervision	44	–	15
**Training costs**	**357**	**13**	**189**
Routine (financial)	157	1	57
Routine (opportunity) – time costs for government staff and volunteers	119	10	83
Supporting (financial) – training of electronic data collectors	34	1	18
Supporting (financial) – NGO supervision and training support	47	1	31
**Drug delivery**	**454**	**23**	**634**
Routine (financial)	72	1	165
Routine (opportunity) – time costs for government staff and volunteers	329	20	130
Routine (opportunity) – donated drugs	0	1	0
Supporting (financial) – electronic data capture	9	1	154
Supporting (financial) – NGO supervision	44	0	186
Total costs[d]	***1414***	***50***	***1214***
Routine (financial)	*311*	*2*	*360*
Routine (opportunity)	*594*	*38*	*253*
Supporting (financial)	*508*	*10*	*601*

Acronyms: non-governmental organization (NGO)
[a] Analysis includes two years of SBD. In India, SBD was conducted bi-annually, so results are presented as the average across four rounds.
[b] Analysis includes two years of SBD. In Malawi and Benin, SBD was conducted annually, so results are presented as the average of two rounds.
[c] Government staff include supervisory and implementing staff whose salaries are paid by the Ministry of Health. Examples include nurses and health officers, teachers, and national and subnational government officials involved in the program.
[d] Routine and supporting activities and related resources are described in Appendix Table 4. Financial costs represent actual expenditure on goods and services purchased by the government or NGO implementing partner. Opportunity costs, on the other hand, include costs forgone by using a resource in a particular way. These opportunity costs recognize and value the cost of using resources, as these resources are then unavailable for productive use elsewhere. Opportunity costs in this analysis include costs of donated albendazole, volunteer time spent on the project (such as volunteer drug distributors), and estimated government staff salary costs.

For peer review only

Table 10: Annual costs of additional deworming program activities, including censuses, prevalence surveys, and coverage surveys, across two years of implementation (2019 local currency)

Activity	Metric	Benin (XOF)		India (INR)		Malawi (MWK)	
		Year 1	Year2	Year 1	Year2	Year 1	Year2
Census[a]	Population censused	94,969	88,647	140,932	146,321	121,819	119,418
	Total cost	100,718,503	58,290,985	6,521,106	5,603,244	151,397,733	101,756,373
	Cost per person censused	1,061	658	46	38	1,243	852
Prevalence survey[a]	Populated surveyed	6,814	5,283	6,503	6,158	6,935	–
	Total cost	74,426,119	67,624,070	5,487,075	12,480,670	93,810,394	–
	Cost per person surveyed	10,923	12,800	844	2,027	13,527	–
Coverage survey[a]	Populated surveyed	16,339	16,130	15,573	14,809	16,796	17,166
	Total cost	44,466,307	38,946,531	1,459,531	1,392,526	51,862,081	41,869,871
	Cost per person surveyed	2,721	2,415	94	94	3,088	2,439

[a]For activities that spanned all 40 clusters, about 50% of the individuals surveyed were from intervention clusters, and the other 50% from control clusters.

For peer review only

For peer review only - http://bmjopen.bmj.com/site/about/guidelines.xhtml

Appendix 5: Additional costing results

In the following tables and figures, we present supplemental costing data not presented in the manuscript, including a further breakdown of costs by supporting vs routine activities, fixed vs variable inputs, and costs across rounds.

Table 11: Percentage of DeWorm3 activity unit costs that are fixed vs. variable, by activity and country

	Benin		India		Malawi	
activity	Fixed unit cost	Variable unit cost	Fixed unit cost	Variable unit cost	Fixed unit cost	Variable unit cost
**Census1**	26%	74%	27%	73%	15%	85%
**Census2**	59%	41%	28%	72%	26%	74%
**Coverage survey1**	25%	75%	27%	73%	13%	87%
**Coverage survey2**	27%	73%	32%	68%	15%	85%
**Coverage survey3**	16%	84%	28%	72%	17%	83%
**Coverage survey4**	14%	86%	28%	72%	17%	83%
**Prevalence survey1**	34%	66%	40%	60%	14%	86%
**Prevalence survey2**	63%	37%	42%	58%	–	–
**cMDA1**	23%	77%	23%	77%	16%	84%
**cMDA2**	25%	75%	26%	74%	17%	83%
**cMDA3**	33%	67%	23%	77%	19%	81%
**cMDA4**	26%	74%	24%	76%	18%	82%
**SBD1**	–	–	7%	93%	–	–
**SBD2**	16%	84%	9%	91%	19%	81%
**SBD3**	–	–	8%	92%	–	–
**SBD4**	21%	79%	8%	92%	19%	81%

Acronyms: community-wide mass drug administration (cMDA), school-based deworming (SBD)

For peer review only

Figure 1: Total financial and opportunity costs of community-wide mass drug administration and school-based deworming by input-classification (including routine and supporting program costs).

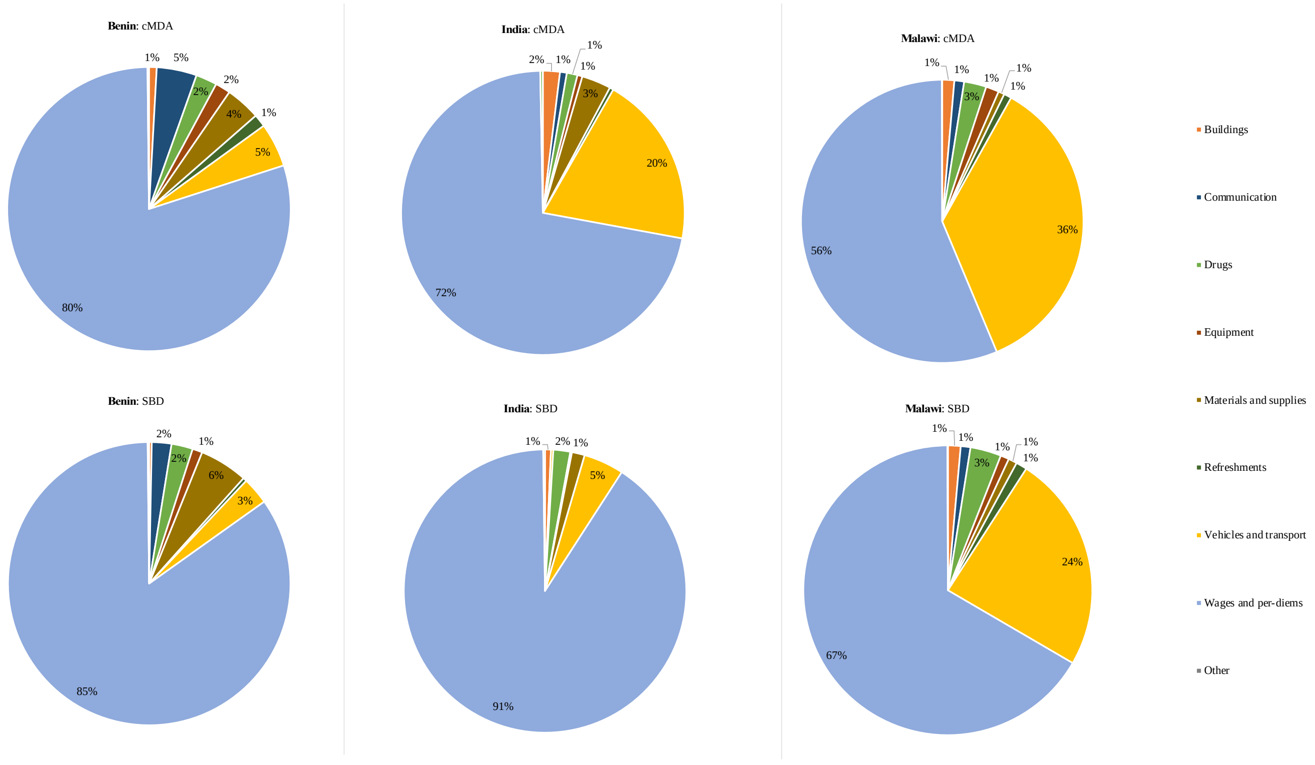

Acronyms: community-wide mass drug administration (cMDA), school-based deworming (SBD)

For peer review only - http://bmjopen.bmj.com/site/about/guidelines.xhtml

Table 12: Community-wide mass drug administration costs by input classification, by routine vs. supporting costs

Category	Benin		India		Malawi	
	Routine	Supporting	Routine	Supporting	Routine	Supporting
Buildings and overheads	1%	1%	2%	2%	1%	1%
Communication	5%	5%	1%	1%	1%	1%
Drugs	0%	–	3%	–	0%	–
Equipment and overheads	2%	2%	1%	1%	3%	1%
Materials and supplies	7%	2%	6%	2%	1%	1%
Other	0%	0%	0%	0%	0%	0%
Refreshments	2%	1%	1%	0%	2%	0%
Vehicles and overheads	5%	5%	4%	28%	13%	48%
Wages and per-diems	78%	85%	81%	66%	78%	48%

Table 13: School-based deworming costs by input classification, by routine vs. supporting costs

Category	Benin		India		Malawi	
	Routine	Supporting	Routine	Supporting	Routine	Supporting
Buildings and overheads	0%	1%	0%	3%	1%	2%
Communication	2%	3%	–	1%	1%	1%
Drugs	0%	–	2%	–	0%	–
Equipment and overheads	0%	3%	–	1%	1%	1%
Materials and supplies	7%	3%	1%	2%	1%	1%
Other	0%	0%	0%	0%	0%	0%
Refreshments	0%	0%	–	–	2%	0%
Vehicles and overheads	2%	5%	0%	21%	11%	40%
Wages and per-diems	88%	85%	96%	71%	83%	55%

For peer review only

For peer review only - http://bmjopen.bmj.com/site/about/guidelines.xhtml

Figure 2: Share of community-wide mass drug administration costs, by country, sub-activity, and category

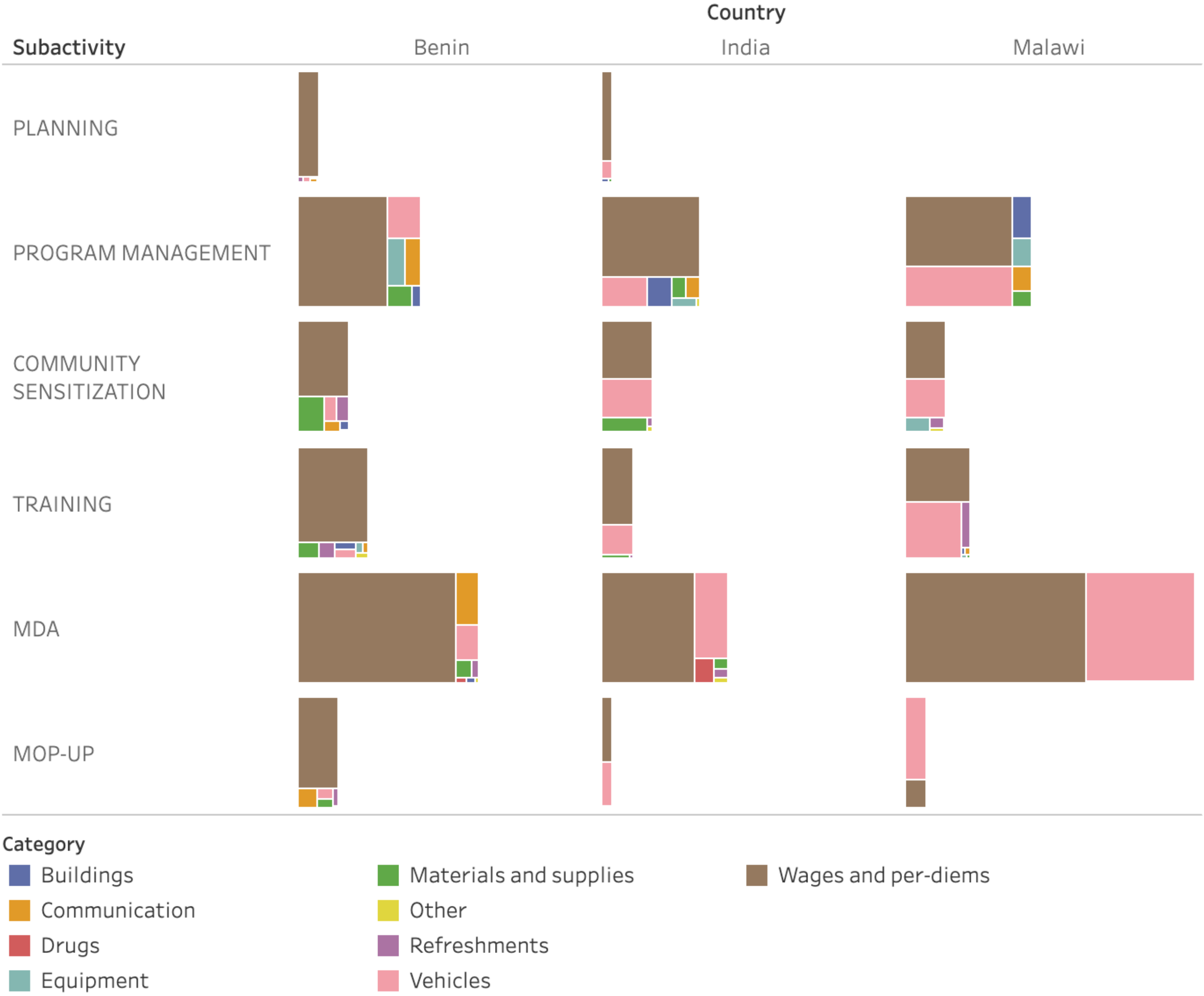

nly

For peer review only - http://bmjopen.bmj.com/site/about/guidelines.xhtml

Figure 3: Box and whisker plots describing variability in median and range of sub-activity costs across four rounds of community-wide mass drug administration in Benin, India, and Malawi; two rounds of school-based deworming in Benin and Malawi; and, four rounds of school-based deworming in India

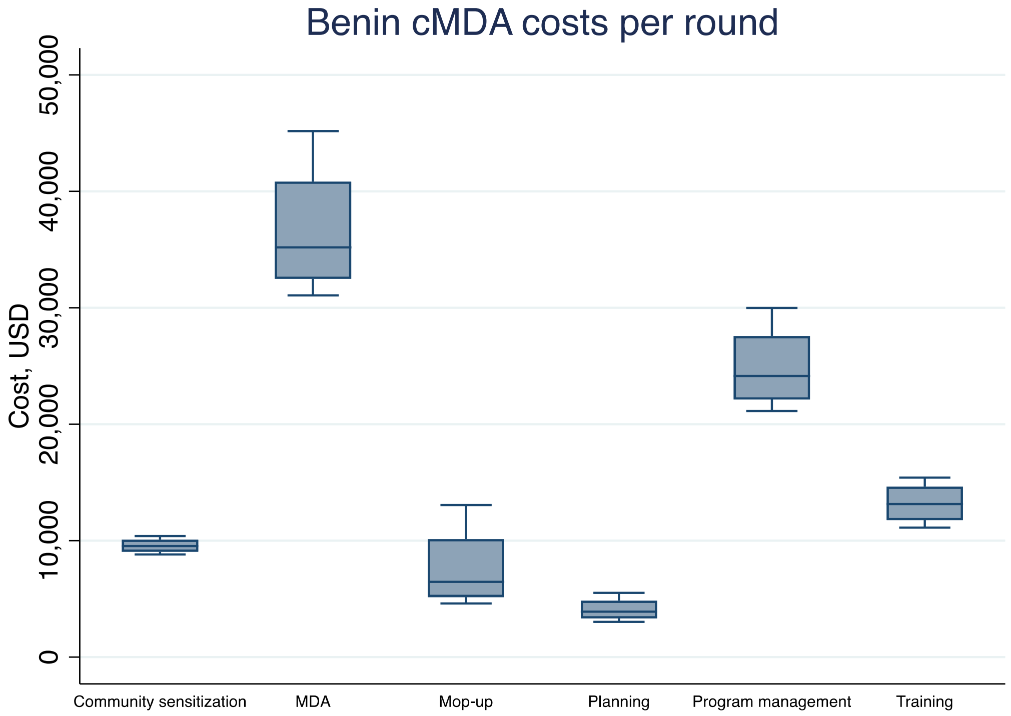

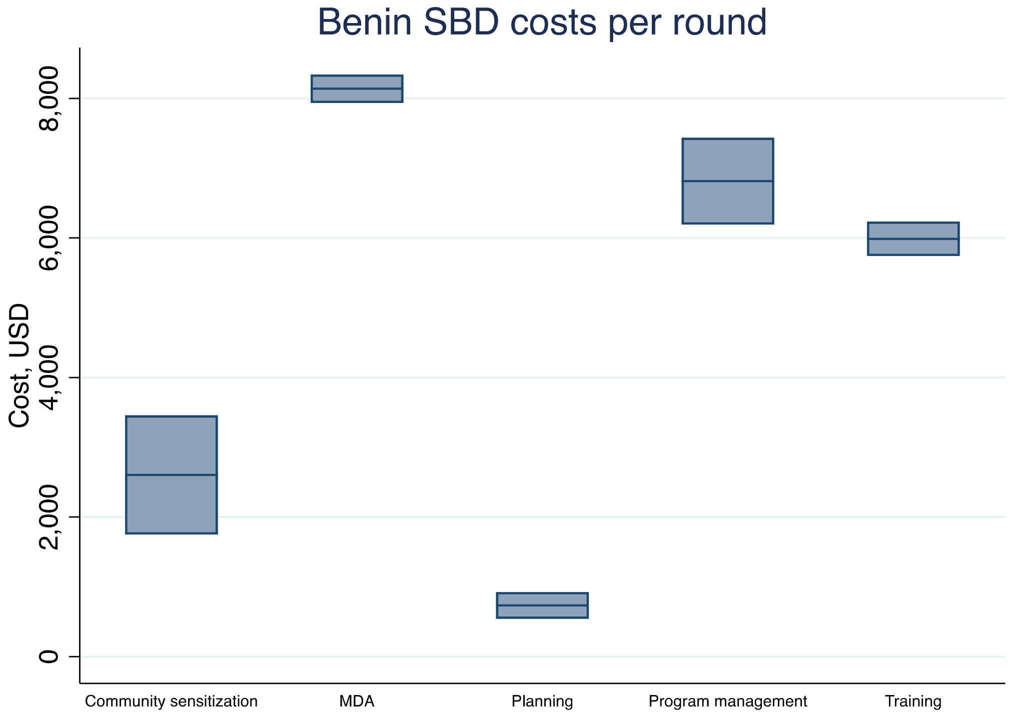

For peer review only - http://bmjopen.bmj.com/site/about/guidelines.xhtml

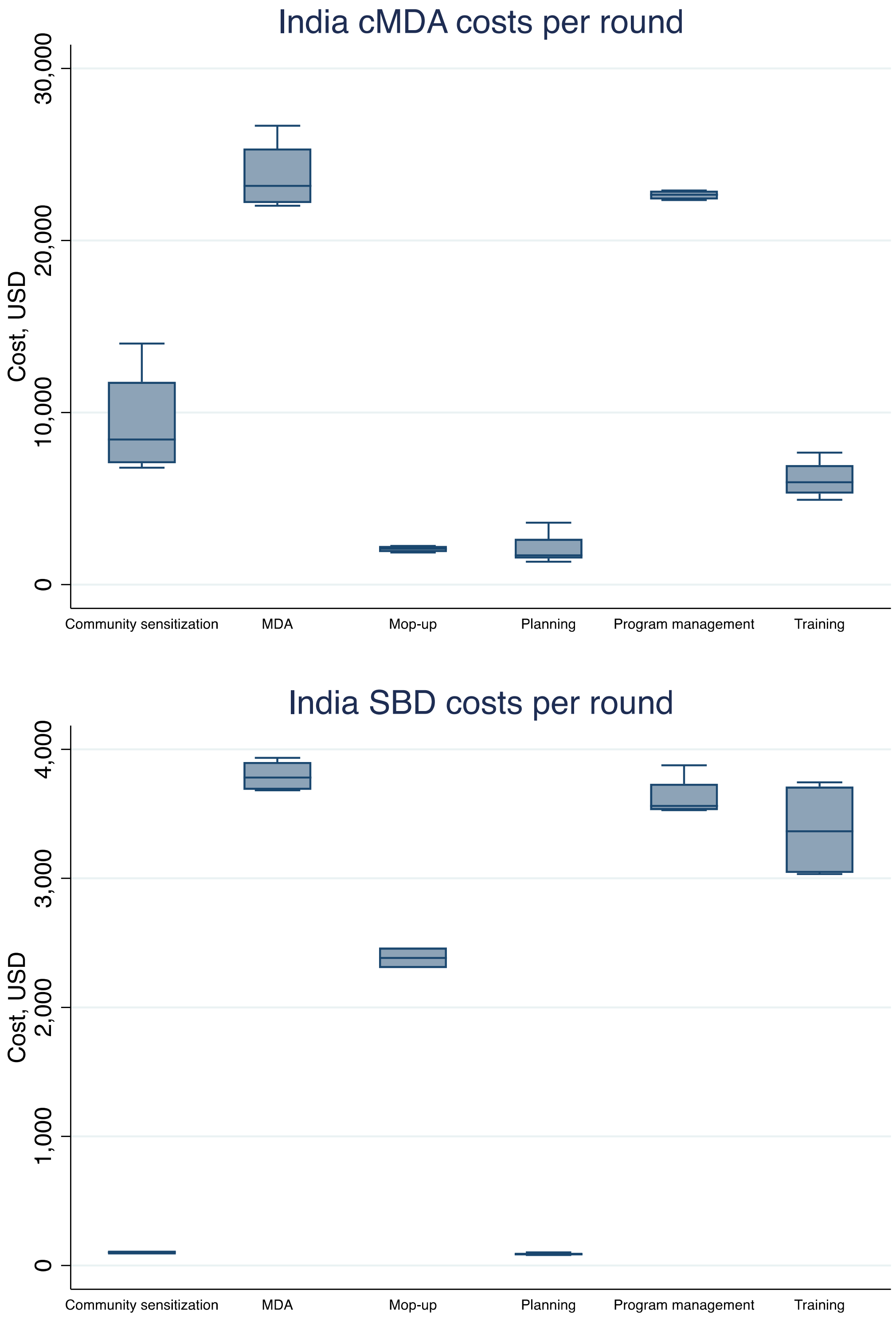
India cMDA costs per round
Cost, USD
30,000
20,000
10,000
0
Community sensitization
MDA
Mop-up
Planning
Program management
Training
India SBD costs per round
Cost, USD
4,000
3,000
2,000
1,000
0
Community sensitization
MDA
Mop-up
Planning
Program management
Training

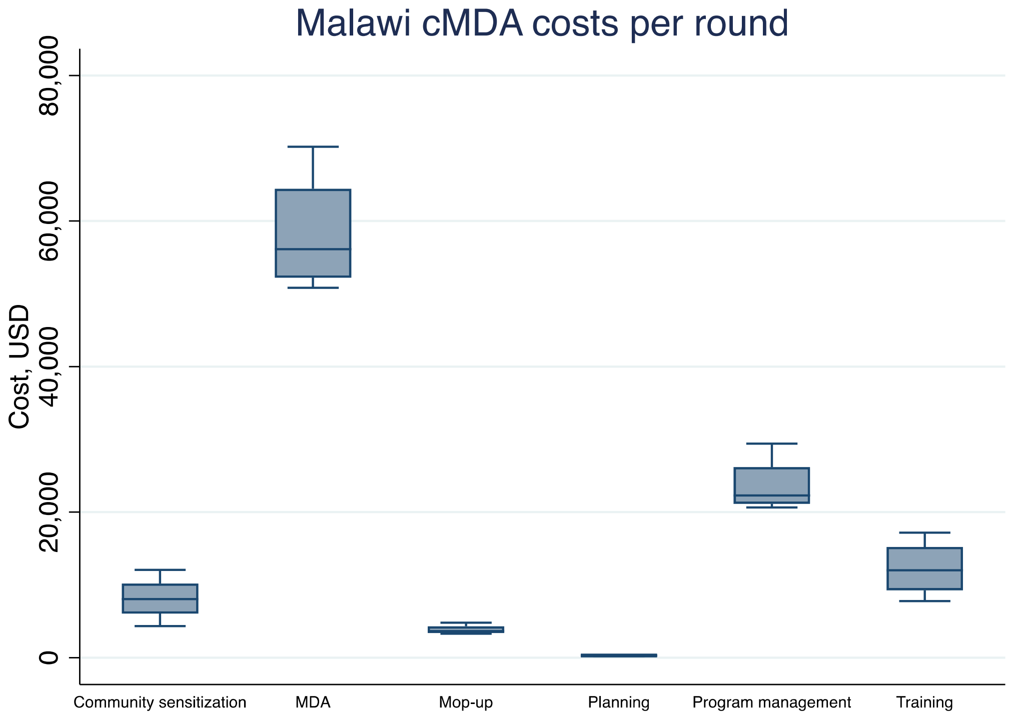

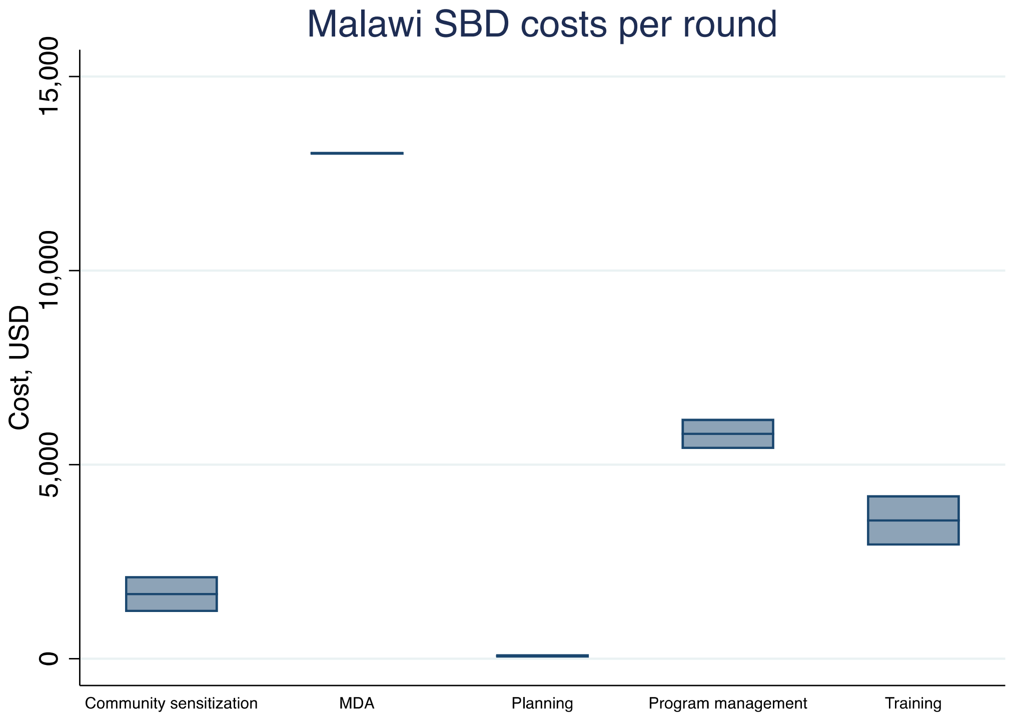

Acronyms: community-wide mass drug administration (cMDA), school-based deworming (SBD)

For peer review only - http://bmjopen.bmj.com/site/about/guidelines.xhtml

Table 14: Annual costs of additional deworming program activities, including censuses, prevalence surveys and coverage surveys, across two years of implementation (2019 USD ($))

	Benin: Year 1	Benin: Year 2	India: Year 1	India: Year 2	Malawi: Year 1	Malawi: Year 2
**Annual census**[a]						
Population censused	94,969	88,647	140,932	146,321	121,819	119,418
Total cost	$ 171,889	$ 99,481	$ 92,603	$ 79,569	$ 203,071	$ 136,487
Cost per person censused	$ 1.81	$ 1.12	$ 0.66	$ 0.54	$ 1.67	$ 1.14
**Annual prevalence survey** following a cohort of approximately 5,000-7,000 individuals[a]						
Populated surveyed	6,814	5,283	6,503	6,158	6,935	—
Total cost	$ 127,018	$ 115,409	$ 77,919	$ 177,231	$ 125,829	—
Cost per person surveyed	$ 18.64	$ 21.85	$ 11.98	$ 28.78	$ 18.14	—
**Bi-annual coverage survey** conducted after each round of cMDA, sampling approximately 8,000 individuals[a]						
Populated surveyed	16,339	16,130	15,573	14,809	16,796	17,166
Total cost	$ 75,887	$ 66,467	$ 20,726	$ 19,774	$ 69,563	$ 56,160
Cost per person surveyed	$ 4.64	$ 4.12	$ 1.33	$ 1.34	$ 4.14	$ 3.27

[a] Activity spanned all 40 clusters, with about 50% of the individuals surveyed were from intervention clusters, and the other 50% from control clusters.

For peer review only - http://bmjopen.bmj.com/site/about/guidelines.xhtml

Appendix 6: Description of cost differences across countries

In the following tables, we provide further details and reasoning regarding differences in observed cMDA and SBD costs across countries.

Table 15: Drivers of heterogeneity in costs across sites

Type of difference	Description	Differences in costs across countries
Number of persons targeted and treated	The number of persons censused in each study site varied, with the smallest population in Benin (approx. 90,000), followed by a larger population in Malawi (approx. 120,000), and the largest censused population in India (approx. 145,000). These differences in overall population sizes affected the total number of persons targeted and treated via community-wide mass drug administration (cMDA).<br><br>Additionally, the age composition in each site varied, leading to variability in the number of targeted children for school-based delivery (SBD). The percent of the population that was pre-school or school-aged was lowest in India, followed by Benin, and highest in Malawi. The number of children who were treated through SBD was lowest in Benin, then increased in India, and was highest in Malawi.	The number of persons treated may have affected overall costs per round of treatment, as more resources may have been required to reach a larger targeted population.<br><br>The number of persons treated had a large effect on the unit costs (cost per treatment administered). For example, the total costs per round of SBD were similar in Malawi and Benin (see Table 1, main text), however, the unit costs were much lower in Malawi given the larger number of children treated via SBD (approximately 50% more children were treated in Malawi than in Benin).
Costs per input-classification	Vehicle costs in Malawi were substantially higher than in Benin and India. Reasons for higher costs include more project vehicles (5 total vehicles were used in Malawi, compared to 2 vehicles in Benin and 1 in India). Additionally, the Malawi DeWorm3 team chose to organize travel for enumerators and HSAs centrally, requiring more car hires and fuel. When cars and drivers were hired to support activities, they were hired for more days in Malawi than in other countries, as MDA was generally 3-4 days longer in Malawi (see Appendix Table 5). In India and Benin, enumerators and CDDs were provided travel allowances, which resulted in lower overall vehicle costs.	When examining cMDA and SBD costs by input-classification (Appendix Figure 1), Malawi had a substantially higher percentage of costs that were allocated to vehicles and overheads, compared to India and Benin. Total costs per round of cMDA were generally highest in Malawi, in partial, due to vehicle costs. The highest cost of cMDA was observed in Malawi round 1, driven by a larger number of vehicles rented.
Planning and program management costs	Resources for planning and program management varied across sites.<br><br>More time was spent on planning in Benin, leading to higher planning costs. Additionally, full-time equivalent costs for central DeWorm3 staff were higher in Benin, leading to higher program management costs.<br><br>Involvement of the DeWorm3 team in SBD varied across countries, and therefore the share of DeWorm3 program management costs allocated to SBD varied across countries. In India, SBD was implemented by the government through the bi-annual National Deworming Day (NDD). Therefore, the DeWorm3 team was only minorly involved in SBD delivery, mainly to observe and record data. In Malawi, the DeWorm3 team was solely responsible for implementing SBD in DeWorm3 clusters, with light supervision from the government. In Benin, the implementation of SBD was led by the government, however, the DeWorm3 team was heavily involved in the coordination and supervision.	cMDA planning and supervision costs were highest in Benin, followed by Malawi, and lowest in India, generally driven by wages (Benin) and vehicles (Malawi).<br><br>Program management costs for SBD were much lower in India, given the DeWorm3 team provided less implementation support and supervision compared to the other countries.
Opportunity costs for albendazole	Albendazole used in the project was donated, however, common practice in costing analyses is to estimate the opportunity costs of drugs (i.e., the costs of the drugs if they were used for other purposes, rather than donated). Albendazole is locally produced in India, so we estimated opportunity costs in India using the local per-tablet price. In Benin and Malawi, albendazole is procured from global suppliers. We estimated opportunity costs in Benin and Malawi as the GlaxoSmithKline valuation of donated albendazole, as $0.045 per-tablet, plus the estimated costs of shipping at $0.0019, for a total value of $0.052.[1] We also estimated approximately 10% buffer stock.	Opportunity costs of albendazole are lower in India than in Benin and Malawi, resulting in about a $0.05 difference in unit costs.

Opportunity costs for government-funded staff and volunteers	The number, type, salaries, and time involved for currently employed government staff and volunteers varied across settings.<br><br>CDDs and volunteers: In Malawi, the primary drug distributors (HSAs), were government-funded staff whereas the primary drug distributors in Benin and India (CDDs) were volunteers. Fewer HSAs were involved in Malawi compared to CDDs in Benin and India, however, salaries were higher in Malawi.<br><br>Teachers and school staff: Malawi had the fewest number of teachers and school staff involved in drug delivery, with more staff involved in Benin, and the greatest number in India. School directors were also involved in SBD in Benin. However, the time spent by teachers on delivery varied, with the smallest amount of time in India, and the greatest amount in Malawi. Monthly salaries for teachers in Benin and India were similar; teacher salaries were approximately 50% lower in Malawi. See Appendix Table 5 for more details.<br><br>Government supervisors: Fewer government staff were involved in the supervision of SBD and cMDA in Malawi, as the DeWorm3 team was the primary implementer.	Opportunity costs were similar across countries for cMDA.<br><br>A large number of school staff in Benin and India (including additional involvement of Anganwadi Workers in India and school directors in Benin) and higher teachers' salaries led to higher opportunity costs for SBD. School staff opportunity costs were lowest in Malawi.
Financial resources for community sensitization	Community sensitization activities varied across sites.<br><br>Benin activities included community-level meetings, public criers, radio broadcasts, and printed materials. Benin also included teacher sensitization for SBD in year 2.<br><br>India activities focused on distributing printed materials (banners and flyers), and community-level meetings (cMDA only).<br><br>Malawi activities included community-level meetings, public announcements, drama groups, and a football bonanza (round 4).<br><br>See Appendix Table 2, for more details.	Sensitization costs were highest in Benin, due to more activities implemented.<br><br>Costs for SBD sensitization were substantially lower in India, as costs were only related to printed materials.
Financial resources for training	In India, SBD is routinely conducted bi-annually and resources for implementation are kept quite low. The only routine financial costs reported by the government were transport allowances provided to teachers. The DeWorm3 team's involvement in SBD training was minimal.<br><br>In Benin and Malawi, the DeWorm3 team was involved in training, and therefore more financial costs were incurred such as printed materials, refreshments, and equipment and hall hires for training sessions.<br><br>Similarly, Benin and Malawi used more financial resources such as equipment, mobile minutes, and refreshments for cMDA training, compared to India.	Financial costs for SBD and cMDA training were substantially lower in India, likely because it was completely government-led.
Financial resources for drug delivery: SBD	In India, SBD is routinely conducted bi-annually and resources for implementation are kept quite low. In the DeWorm3 project, SBD continued to be implemented through the government routinely. Few financial resources are required during drug delivery, only allowances for some key staff (VHNs, ASHAs, and for supervision).<br><br>In Benin and Malawi, the DeWorm3 team was more involved in drug delivery. Therefore, more resources were used such as fuel, allowances (such as travel, communication, lunch) for CDDs/HSAs, refreshments, and allowances for DeWorm3 coordinating and supervisory staff.	Financial costs for SBD were substantially lower in India, likely because it was completely government-led with involvement from DeWorm3 limited to data collection.
Financial resources for drug delivery: cMDA	In Benin, the DeWorm3 team collaborated closely with the Ministry of Health to implement cMDA. Therefore, many allowances and travel costs were incurred for supervision and coordination efforts by both the Ministry of Health and the DeWorm3 team. Additionally, cMDA mop-up required more resources in Benin, mainly due to a large mop-up campaign in cMDA round 4. In Benin, cMDA round 4 was interrupted by a natural disaster (flooding). To reach higher coverage rates, a more involved mop-up campaign was implemented one month after MDA, with additional sensitization and training.<br><br>In India and Malawi, the DeWorm3 was primarily responsible for cMDA drug delivery, with few allowances paid to government supervisors.	Routine financial costs were higher in Benin, due to more supervision costs and the more involved mop-up campaign in round 4 of MDA.

Appendix 7: Appendix references

1 Turner HC, Bettis AA, Chu BK, et al. Investment Success in Public Health: An Analysis of the Cost-Effectiveness and Cost-Benefit of the Global Programme to Eliminate Lymphatic Filariasis. *Clin Infect Dis* 2017; **64**: 728–35.

2 International Labor Organization. ILOSTAT database. https://ilostat.ilo.org/data (accessed Aug 25, 2021).

3 Tamil Nadu State Government Department of Evaluation and Applied Research. Tamil Nadu - An Economic Appraisal 2011-12 to 2013-14.

4 Turner HC, Bettis AA, Chu BK, et al. Investment Success in Public Health: An Analysis of the Cost-Effectiveness and Cost-Benefit of the Global Programme to Eliminate Lymphatic Filariasis. *Clin Infect Dis* 2017; **64**: 728–35.

5 GlaxoSmithKline. Neglected Tropical Diseases: Our commitment. 2021; published online Jan. https://www.gsk.com/media/6543/ntd-factsheet-jan2021.pdf.

6 International Labor Organization. ILOSTAT database. https://ilostat.ilo.org/data (accessed Aug 25, 2021).

7 Tamil Nadu State Government Department of Evaluation and Applied Research. Tamil Nadu - An Economic Appraisal 2011-12 to 2013-14.

8 Serje J, Bertram MY, Brindley C, et al. Global health worker salary estimates: an econometric analysis of global earnings data. *Cost Eff Resour Alloc* 2018; **16**: 10.

9 Werkman M, Toor J, Vegvari C, et al. Defining stopping criteria for ending randomized clinical trials that investigate the interruption of transmission of soil-transmitted helminths employing mass drug administration. *PLoS Negl Trop Dis* 2018; **12**: e0006864.

For peer review only

CHEERS Checklist

Items to include when reporting economic evaluations of health interventions

The **ISPOR CHEERS Task Force Report**, *Consolidated Health Economic Evaluation Reporting Standards (CHEERS)—Explanation and Elaboration: A Report of the ISPOR Health Economic Evaluations Publication Guidelines Good Reporting Practices Task Force*, provides examples and further discussion of the 24-item CHEERS Checklist and the CHEERS Statement. It may be accessed via the *Value in Health* or via the ISPOR Health Economic Evaluation Publication Guidelines – CHEERS: Good Reporting Practices webpage: http://www.ispor.org/TaskForces/EconomicPubGuidelines.asp

Section/item	Item No	Recommendation	Reported on page No/ line No
**Title and abstract**			
Title	1	Identify the study as an economic evaluation or use more specific terms such as "cost-effectiveness analysis", and describe the interventions compared.	
Abstract	2	Provide a structured summary of objectives, perspective, setting, methods (including study design and inputs), results (including base case and uncertainty analyses), and conclusions.	
**Introduction**			
Background and objectives	3	Provide an explicit statement of the broader context for the study. Present the study question and its relevance for health policy or practice decisions.	
**Methods**			
Target population and subgroups	4	Describe characteristics of the base case population and subgroups analysed, including why they were chosen.	
Setting and location	5	State relevant aspects of the system(s) in which the decision(s) need(s) to be made.	
Study perspective	6	Describe the perspective of the study and relate this to the costs being evaluated.	
Comparators	7	Describe the interventions or strategies being compared and state why they were chosen.	
Time horizon	8	State the time horizon(s) over which costs and consequences are being evaluated and say why appropriate.	
Discount rate	9	Report the choice of discount rate(s) used for costs and outcomes and say why appropriate.	
Choice of health outcomes	10	Describe what outcomes were used as the measure(s) of benefit in the evaluation and their relevance for the type of analysis performed.	
Measurement of effectiveness	11a	*Single study-based estimates:* Describe fully the design features of the single effectiveness study and why the single study was a sufficient source of clinical effectiveness data.	

For peer review only

	11b	*Synthesis-based estimates:* Describe fully the methods used for identification of included studies and synthesis of clinical effectiveness data.	
Measurement and valuation of preference based outcomes	12	If applicable, describe the population and methods used to elicit preferences for outcomes.	
Estimating resources and costs	13a	*Single study-based economic evaluation:* Describe approaches used to estimate resource use associated with the alternative interventions. Describe primary or secondary research methods for valuing each resource item in terms of its unit cost. Describe any adjustments made to approximate to opportunity costs.	
	13b	*Model-based economic evaluation:* Describe approaches and data sources used to estimate resource use associated with model health states. Describe primary or secondary research methods for valuing each resource item in terms of its unit cost. Describe any adjustments made to approximate to opportunity costs.	
Currency, price date, and conversion	14	Report the dates of the estimated resource quantities and unit costs. Describe methods for adjusting estimated unit costs to the year of reported costs if necessary. Describe methods for converting costs into a common currency base and the exchange rate.	
Choice of model	15	Describe and give reasons for the specific type of decision-analytical model used. Providing a figure to show model structure is strongly recommended.	
Assumptions	16	Describe all structural or other assumptions underpinning the decision-analytical model.	
Analytical methods	17	Describe all analytical methods supporting the evaluation. This could include methods for dealing with skewed, missing, or censored data; extrapolation methods; methods for pooling data; approaches to validate or make adjustments (such as half cycle corrections) to a model; and methods for handling population heterogeneity and uncertainty.	
**Results**			
Study parameters	18	Report the values, ranges, references, and, if used, probability distributions for all parameters. Report reasons or sources for distributions used to represent uncertainty where appropriate. Providing a table to show the input values is strongly recommended.	
Incremental costs and outcomes	19	For each intervention, report mean values for the main categories of estimated costs and outcomes of interest, as well as mean differences between the comparator groups. If applicable, report incremental cost-effectiveness ratios.	
Characterising uncertainty	20a	*Single study-based economic evaluation:* Describe the effects of sampling uncertainty for the estimated incremental cost and incremental effectiveness parameters, together with the impact	

For peer review only

		of methodological assumptions (such as discount rate, study perspective).	
	20b	*Model-based economic evaluation:* Describe the effects on the results of uncertainty for all input parameters, and uncertainty related to the structure of the model and assumptions.	
Characterising heterogeneity	21	If applicable, report differences in costs, outcomes, or cost-effectiveness that can be explained by variations between subgroups of patients with different baseline characteristics or other observed variability in effects that are not reducible by more information.	
**Discussion**			
Study findings, limitations, generalisability, and current knowledge	22	Summarise key study findings and describe how they support the conclusions reached. Discuss limitations and the generalisability of the findings and how the findings fit with current knowledge.	
**Other**			
Source of funding	23	Describe how the study was funded and the role of the funder in the identification, design, conduct, and reporting of the analysis. Describe other non-monetary sources of support.	
Conflicts of interest	24	Describe any potential for conflict of interest of study contributors in accordance with journal policy. In the absence of a journal policy, we recommend authors comply with International Committee of Medical Journal Editors recommendations.	

For consistency, the CHEERS Statement checklist format is based on the format of the CONSORT statement checklist

The **ISPOR CHEERS Task Force Report** provides examples and further discussion of the 24-item CHEERS Checklist and the CHEERS Statement. It may be accessed via the *Value in Health* link or via the ISPOR Health Economic Evaluation Publication Guidelines – CHEERS: Good Reporting Practices webpage: http://www.ispor.org/TaskForces/EconomicPubGuidelines.asp

The citation for the CHEERS Task Force Report is:
Husereau D, Drummond M, Petrou S, et al. Consolidated health economic evaluation reporting standards (CHEERS)—Explanation and elaboration: A report of the ISPOR health economic evaluations publication guidelines good reporting practices task force. Value Health 2013;16:231-50.

For peer review only

BMJ Open

Costs of community-wide mass drug administration and school-based deworming for soil-transmitted helminths: evidence from a randomized-controlled trial in Benin, India, and Malawi

Journal:	*BMJ Open*
Manuscript ID	bmjopen-2021-059565.R1
Article Type:	Original research
Date Submitted by the Author:	22-Apr-2022

Complete List of Authors:	Morozoff, Chloe; University of Washington, Global Health<br>Avokpaho, Euripide; Institut de Recherche Clinique du Benin<br>PUTHUPALAYAM KALIAPPAN, SARAVANAKUMAR; Christian Medical College Vellore, Division of Gastrointestinal Sciences<br>Simwanza, James; Blantyre Institute for Community Outreach<br>Gideon, Samuel; Christian Medical College Vellore, Division of Gastrointestinal Sciences<br>Lungu, Wongani; Blantyre Institute for Community Outreach<br>Houngbegnon, Parfait; Institut de Recherche Clinique du Benin<br>Galactionova, Katya; Swiss Tropical and Public Health Institute; University of Basel<br>Sahu, Maitreyi; University of Washington, Global Health<br>Kalua, Khumbo; Blantyre Institute for Community Outreach<br>Luty, Adrian J. F.; Universite de Paris, MERIT, IRD<br>Ibikounle, Moudachirou; Institut de Recherche Clinique du Benin; Universite d'Abomey-Calavi, Centre de Recherche pour la lutte contre les Maladies Infectieuses Tropicales (CReMIT/TIDRC)<br>Bailey, Robin; London School of Hygiene & Tropical Medicine, Clinical Research Department<br>Pullan, Rachel; London School of Hygiene & Tropical Medicine, Department of Disease Control<br>Ajjampur, Sitara; Christian Medical College Vellore, Division of Gastrointestinal Sciences<br>Walson, Judd; University of Washington, Global Health<br>Means, Arianna; University of Washington, Global Health

**Primary Subject Heading**:	Global health
Secondary Subject Heading:	Health economics, Infectious diseases
Keywords:	Health economics < HEALTH SERVICES ADMINISTRATION & MANAGEMENT, Tropical medicine < INFECTIOUS DISEASES, Public health < INFECTIOUS DISEASES

For peer review only - http://bmjopen.bmj.com/site/about/guidelines.xhtml

SCHOLARONE™
Manuscripts

I, the Submitting Author has the right to grant and does grant on behalf of all authors of the Work (as defined in the below author licence), an exclusive licence and/or a non-exclusive licence for contributions from authors who are: i) UK Crown employees; ii) where BMJ has agreed a CC-BY licence shall apply, and/or iii) in accordance with the terms applicable for US Federal Government officers or employees acting as part of their official duties; on a worldwide, perpetual, irrevocable, royalty-free basis to BMJ Publishing Group Ltd ("BMJ") its licensees and where the relevant Journal is co-owned by BMJ to the co-owners of the Journal, to publish the Work in this journal and any other BMJ products and to exploit all rights, as set out in our licence.

The Submitting Author accepts and understands that any supply made under these terms is made by BMJ to the Submitting Author unless you are acting as an employee on behalf of your employer or a postgraduate student of an affiliated institution which is paying any applicable article publishing charge ("APC") for Open Access articles. Where the Submitting Author wishes to make the Work available on an Open Access basis (and intends to pay the relevant APC), the terms of reuse of such Open Access shall be governed by a Creative Commons licence – details of these licences and which Creative Commons licence will apply to this Work are set out in our licence referred to above.

Other than as permitted in any relevant BMJ Author's Self Archiving Policies, I confirm this Work has not been accepted for publication elsewhere, is not being considered for publication elsewhere and does not duplicate material already published. I confirm all authors consent to publication of this Work and authorise the granting of this licence.

For peer review only

Costs of community-wide mass drug administration and school-based deworming for soil-transmitted helminths: evidence from a randomized-controlled trial in Benin, India, and Malawi

Chloe Morozoff, MPH[1], Euripide Avokpaho, MD[2], Saravanakumar Puthupalayam Kaliappan, PhD[3], James Simwanza, BA[4], Samuel Paul Gideon, MBA[3], Wongani Lungu, ACCA[4], Parfait Houngbegnon, PhD[2], Katya Galactionova, PhD[5,6], Maitreyi Sahu, MSPH[1], Khumbo Kalua, PhD[4], Adrian J F Luty, PhD[7], Moudachirou Ibikounlé, PhD[2,8], Robin Bailey, PhD[9], Rachel Pullan, PhD[10], Sitara Swarna Rao Ajjampur, PhD[3], Judd Walson, MD[1], Arianna Rubin Means, PhD[1*]

[1] Department of Global Health, University of Washington, Seattle, Washington, United States

[2] Institut de Recherche Clinique du Bénin, Abomey-Calavi, Bénin

[3] The Wellcome Trust Research Laboratory, Division of Gastrointestinal Sciences, Christian Medical College, Vellore, India

[4] Blantyre Institute for Community Outreach, Blantyre, Malawi

[5] Swiss Tropical and Public Health Institute, Basel, Switzerland

[6] University of Basel, Basel, Switzerland

[7] Université de Paris, MERIT, IRD, Paris, France

[8] Centre de Recherche pour la lutte contre les Maladies Infectieuses Tropicales (CReMIT/TIDRC), Université d'Abomey-Calavi, Abomey-Calavi, Bénin

[9] Clinical Research Department, London School of Hygiene & Tropical Medicine, London, United Kingdom

[10] Faculty of Infectious and Tropical Diseases, London School of Hygiene and Tropical Medicine, London, United Kingdom

***Corresponding author:**

Arianna Rubin Means, PhD

Hans Rosling Center Box 351620

3980 15th Ave NE, Seattle, WA 98195

Tel: +1 (206) 370 0225 | Email: aerubin@uw.edu

Word count:

Abstract: 300

Main text: 4,240

For peer review only

ABSTRACT

Objectives: Current guidelines for the control of soil-transmitted helminths (STH) recommend deworming children and other high-risk groups, primarily using school-based deworming (SBD) programs. However, targeting individuals of all ages through community-wide mass drug administration (cMDA) may interrupt STH transmission in some settings. We compared the costs of cMDA to SBD to inform decision making about future updates to STH policy.

Design: We conducted activity-based micro-costing of cMDA and SBD for two years in Benin, India, and Malawi within an ongoing cMDA trial.

Setting: Field sites and collaborating research institutions.

Primary and secondary outcomes: We calculated total financial and opportunity costs and costs per treatment administered (unit costs) from the service provider perspective, including costs related to community drug distributors and other volunteers.

Results: On average, cMDA unit costs were more expensive than SBD in India ($1.17 vs. $0.72) and Malawi ($2.26 vs. $1.69), and comparable in Benin ($2.45 vs. $2.47). cMDA was more expensive than SBD in part because most costs (~60%) were "supportive costs" needed to deliver treatment with high coverage, such as additional supervision and electronic data capture. A smaller fraction of cMDA costs (~30%) were routine expenditures (e.g. drug distributor allowances). The remaining cMDA costs (~10%) were opportunity costs of staff and volunteer time. A larger percentage of SBD costs were opportunity costs for teachers and other government staff (between ~25%-75%). Unit costs varied over time and were sensitive to the number of treatments administered.

Conclusions: cMDA was generally more expensive than SBD. Accounting for local staff time (volunteers, teachers, health workers) in community programs is important and drives higher cost estimates than commonly recognized in the literature. Costs may be lower outside of a trial setting, given a reduction in supportive costs used to drive higher treatment coverage and economies of scale.

Trial registration number: NCT03014167.

For peer review only

STRENGHTS AND LIMITATIONS OF THIS STUDY

- We used rigorous micro-costing methods to collect costs associated with cMDA and SBD and corresponding treatment data, in real time.
- The granularity of data collected provides rich information regarding the resource needs for deworming programs, and how these may vary across countries and delivery modalities (school versus community-based treatment).
- We estimated opportunity costs of the volunteer workforce and currently employed government staff (e.g. teachers, community drug distributors, supervisors), which are often excluded from deworming costing studies.
- Although costs associated with research and trial administration were not included in this study, it is possible that some costs (e.g., program management, planning, and supervision) may be higher in this research setting than what would be observed in routine deworming programs.

For peer review only

INTRODUCTION

Soil-transmitted helminths (STH) are a group of intestinal parasites (*Ascaris lumbricoides*, *Ancylostoma duodenale*, *Necator americanus*, and *Trichuris trichiura*) that globally affect approximately 1.5 billion individuals annually, predominantly in sub-Saharan Africa, East Asia, and Latin America.[1] Moderate-to-heavy infection with STH is associated with diarrhea, malnutrition, anemia, wasting, stunting, and cognitive delay.[1-2] To reduce the burden of STH morbidity, the World Health Organization (WHO) targets elimination of STH as a public health problem by 2030.[3] Current STH control guidelines recommend preventative chemotherapy (deworming using albendazole or mebendazole) for high-risk populations such as children, non-pregnant adolescent girls, and women of reproductive age.[2]

STH control programs include annual or bi-annual school-based deworming (SBD), where teachers and health workers deliver preventative chemotherapy to pre-school and school-aged children.[2] SBD is a low-cost intervention; SBD leverages existing infrastructure (schools) as a delivery platform while drug costs are low due to global drug donation programs.[4] A review of STH treatment costs estimates SBD costs at US $0.30 per child treated, much lower than the cost of screening and treating a single individual for STH annually ($4.89/person in 2015 US$).[2] Costs of deworming preschool-aged children or other community members outside of schools, is estimated at $0.63 per person treated.[2,5] Although SBD is a low-cost intervention for controlling STH, non-school attending children may be missed by these programs, and reinfection of children within the community from adult reservoirs may require continuous treatment.[6]

It may be possible to interrupt STH transmission by expanding deworming eligibility to individuals of all ages.[7] The DeWorm3 project is an ongoing cluster-randomized trial testing the feasibility of interrupting STH transmission using community-wide mass drug administration (cMDA) in Benin, India, and Malawi.[8-9] If successful, scaling-up cMDA would require evidence on the relative cost compared to standard-of-care SBD. Although studies have evaluated the costs of mass drug administration for neglected tropical diseases, costs vary based on country implementation strategy (e.g., use of volunteers or salaried staff), disease control program, age of program, size of population treated, and costing methods used.[10-18] To our knowledge, there are no studies that directly compare costs of cMDA and SBD for STH control, within the same setting and methodological framework.

This study systematically identified, measured, and compared resources for implementation of twelve rounds of cMDA and eight rounds of SBD across the DeWorm3 sites, during implementation of the trial.

For peer review only

Determining the costs and cost drivers of expanding STH treatment to all individuals in a community will be essential for shaping future STH policy.

METHODS

Overview of DeWorm3

The DeWorm3 project was implemented in Come Commune of Benin, Tamil Nadu State of India, and Mangochi District of Malawi. These sites were selected because they had previously implemented lymphatic filariasis programs over five or more rounds with albendazole co-administered with ivermectin or diethylcarbamazine.[8] In each site, twenty control clusters (minimum population size of 1,650 persons per cluster) were randomized to SBD (either annually or bi-annually, per the country's standard of care) and twenty intervention clusters were randomized to biannual cMDA. In intervention clusters, SBD continued to be implemented as per the country's standard of care but was not costed; during treatment rounds in which SBD was also implemented, cMDA was conducted after SBD (see Figure 1). During the DeWorm3 project, cMDA and SBD were implemented for three years, from 2018-2020. During SBD, teachers distributed albendazole to children, with support from community health workers, known as Community Drug Distributors (CDDs) in Benin, Accredited Social Health Activists (ASHAs) in India, and Health Surveillance Assistants (HSAs) in Malawi. During cMDA, albendazole was delivered door-to-door in the community by community health workers (CDDs in Benin, CDDs and ASHAs in India, HSAs in Malawi) alongside electronic data collectors, referred to as enumerators. For both treatment strategies, supervision was conducted by DeWorm3 and government staff. Number of schools, villages, and other site-level contextual attributes are described in Appendix 1 and Appendix 2.

In addition to deworming, DeWorm3 also conducted an annual census to enumerate the full population in study catchment areas, annual prevalence surveys to determine STH prevalence and intensity, and post-MDA coverage surveys to assess the reach of cMDA and SBD (Figure 1).[8] These activities are not consistently conducted in national deworming programs but could be indicated in future elimination programs that require more intensive monitoring and evaluation.

Costing study design

We conducted activity-based micro-costing from the service provider perspective (Ministry of Health and/or Education) during the first two years of DeWorm3 implementation in order to explore heterogeneity in costs across rounds of implementation. Across the three sites, we conducted two years of intensive micro-costing, resulting in data from 12 rounds of cMDA and 8 rounds of SBD. Costing the first

For peer review only - http://bmjopen.bmj.com/site/about/guidelines.xhtml

year of cMDA implementation allowed us to capture costs related to start-up, while the second year provided a more accurate portrayal of costs related to routine implementation. This analysis includes opportunity costs associated with all health worker involvement in implementation, including teachers and community volunteers engaging in drug delivery. Costs to the household were not assessed as they are assumed to be negligible.[10-11] We measured all resources required to deliver cMDA and SBD in DeWorm3 clusters, resulting in over 8,000 data points, and converted their value into a cost estimate (including borrowed and donated resources).[19-20]

The methodology for costing cMDA is detailed in Galactionova et al, 2021, and additional SBD data collection tools are detailed in Appendix 2.[21] We briefly describe the strategy used. DeWorm3 staff in each site recorded resource use and costs related to the implementation of trial activities within an Excel-based costing tool. Data were collected in real-time and were entered separately for activities including program management (overheads), planning, and each round of the census, prevalence survey, SBD, cMDA, and coverage survey. Within the tool, we also quantified borrowed resources used, such as borrowed vehicles and volunteer time. Other data sources were used to collect or allocate costs not included in the costing tool, such as government expenditures (see Appendix 2).

Following data collection, all costing data were iteratively reviewed for quality and completeness. Costs related to DeWorm3 research only (e.g. qualitative research or school surveys) were not included in the data collection instruments and, if identified, were removed during data cleaning.[22]

Analyzing financial and opportunity costs

Financial costs included actual expenditures on goods and services purchased by the DeWorm3 project or site governments. We analyzed these data in Stata (version 16.1). Costs were converted to USD using the annual average exchange rate based on the year in which the costs were incurred.[23] When costs were shared across multiple activities—such as vehicles or personnel salaries—we allocated costs based on the number of days required to implement each sub-activity. We allocated costs reported at the district or state level via government budgets to the DeWorm3 study area using population proportionate to size estimates. We annualized startup costs over the three-year duration of cMDA and SBD implementation, and capital items based on their useful life years, using a 3% discount rate.[20,24] All costs are presented in 2019 USD; costs incurred before 2019 were inflated using GDP implicit price deflators.[25-26] Costs in local currency are presented in Appendix 3.

For peer review only

Opportunity costs included the costs of donated drugs, volunteer time (CDDs, ASHAs, and community volunteers), and time costs for currently employed government staff. We estimated costs associated with volunteer time spent delivering drugs using the DeWorm3 trial's digital treatment forms (described in Appendix 2). We used country-specific average earnings to estimate the opportunity costs associated with volunteer time (2010-2011 regional annual salary adjusted to relevant year using annual growth rate in India and 2018 national monthly earnings in Benin and Malawi).[27-28] For government staff (e.g. national and district-level personnel, teachers, and health center staff), we collected salaries through Ministry of Health costing surveys. We derived government staff time spent on activities from costing data collection tools, and teacher time spent on SBD from a school survey. We calculated total economic costs (financial plus opportunity costs) per site, per year, and by activity, sub-activity, and input-classification. Key costing inputs such as the number of implementing staff, average salaries, and allowances are described in Appendix 2.

Estimating routine and supportive program costs

Because the DeWorm3 Project included several activities related to the delivery and monitoring of MDA that may not be present in all deworming programs, we classified and distinguished costs as either routine MDA program costs or supportive program costs. Routine program costs included activities typically implemented by a government (e.g. training of CDDs). Supportive costs included additional activities aimed at optimizing coverage and compliance. For example, electronic data were collected to monitor cMDA coverage in real-time and identify areas in need of additional sensitization and mop-up. Generally, supportive activities included: 1) start-up planning costs; 2) additional supervision from a non-governmental organization (NGO) implementing partner; 3) additional sensitization activities; 4) electronic data collection, and 5) program management costs associated with these supportive activities. Additional details regarding routine and supportive costs are presented in Appendix 2.

Unit costs analysis

The cost per treatment administered (i.e. unit cost) was determined by dividing costs per round by the total number of treatments administered. The number of treatments administered via cMDA was abstracted from MDA treatment forms (household-level forms completed by enumerators during cMDA). The number of treatments administered via SBD was estimated from paper SBD forms filled out by school and/or DeWorm3 field staff, then transferred to an electronic format. One and two-way sensitivity analyses were conducted to explore how the average cost per treatment administered would change due to variation in key costing inputs and coverage levels (methods described in Appendix 4).

For peer review only

Patient and public involvement

Community members living in STH endemic areas were not involved in the design, conduct, reporting, or dissemination of this costing study. Ministry of Health and Education staff were involved in the conduct of this costing study (including data collection and dissemination), and in the design and conduct of the wider DeWorm3 trial.

RESULTS

Total costs of cMDA and SBD

Between February 2018 and December 2019, a total of twelve rounds of cMDA and eight rounds of SBD were delivered across DeWorm3 sites in Benin, India, and Malawi. Table 1 details the number of treatments administered, total costs, and unit costs across treatment strategies, sites, and rounds. The total number of treatments administered for a given round of MDA ranged from 9,298 (Benin SBD round 2) to 57,398 (India cMDA round 4). Total costs of SBD ranged from $12,763 in India (round 4) to $25,933 in Benin (round 4), while total costs of cMDA ranged from $61,806 (India, round 4) to $129,369 (Malawi, round 1). Sub-activity costs also varied across rounds, as detailed in Appendix 5.

cMDA unit costs varied from $1.08 in India (round 4) to $2.90 in Benin (round 4). Within sites, cMDA unit costs varied across the four rounds, fluctuating by $0.73 in Benin and Malawi, and $0.21 in India. SBD was generally less expensive than cMDA, with approximately one-third the number of treatments administered and one-quarter of the total costs. SBD unit costs varied from $0.60 in India (round 3) to $2.51 in Benin (round 4). Within sites, SBD costs fluctuated $0.09 across two rounds in Benin, $0.31 across four rounds in India, and $0.46 across two rounds in Malawi.

Table 1: Total economic costs and number of treatments administered through community-wide mass drug administration and school-based deworming, per country-round, in 2019 USD ($).

Metric	Benin		India		Malawi	
	cMDA	SBD	cMDA	SBD	cMDA	SBD
Number of treatments administered[a]						
Round 1	45,280	–	55,953	15,266	49,518	–
Round 2	37,913	9,298	55,758	19,152	38,641	16,077
Round 3	42,398	–	57,353	21,396	52,122	–
Round 4	32,529	10,343	57,398	20,586	49,709	12,964
Total costs[b]						
Round 1	106,695	–	71,969	13,854	129,369	–
Round 2	82,287	22,516	64,416	14,089	97,512	23,251
Round 3	99,664	–	66,129	12,794	97,838	–
Round 4	94,422	25,933	61,806	12,763	100,112	24,812
Cost per treatment administered						
Round 1	2.36	–	1.29	0.91	2.61	–
Round 2	2.17	2.42	1.16	0.74	2.52	1.45
Round 3	2.35	–	1.15	0.60	1.88	–
Round 4	2.90	2.51	1.08	0.62	2.01	1.91

Acronyms: community-wide mass drug administration (cMDA), school-based deworming (SBD)
Note: Dashes (–) represent situations where no data was collected. SBD was only implemented annually in Benin and Malawi, so no data were available for rounds 1 and 3.
[a] Treatments administered for cMDA includes all eligible individuals who received treatment by DeWorm3 through cMDA in the intervention clusters (Source: DeWorm3 MDA treatment logs). Population treated for SBD includes all children treated in schools within the DeWorm3 control clusters (Source: SBD treatment logs).
[b] Total costs include both financial and opportunity costs.

Average unit costs of cMDA and SBD

Activity-specific unit costs for cMDA and SBD are presented in Tables 2 and 3, respectively. Average cMDA unit costs were $2.45 in Benin, $1.17 in India, and $2.26 in Malawi. Routine financial costs were approximately 20-35% of unit costs, at $0.87 in Benin, $0.26 in India, and $0.48 in Malawi. The majority of routine financial costs (approximately 70-80%) were allowances for key implementing staff (e.g., lunch, travel, and/or mobile allowances for CDDs, health center staff, district and national government supervisors, sensitization staff). The remaining portion of routine costs were materials and supplies, equipment or building rentals, and vehicle costs for supervision (Appendix 5). Routine opportunity costs, including donated drugs and government and volunteer time, were approximately 10% of unit costs (ranging from $0.16 in India to $0.30 in Malawi). Supportive program costs, including costs of electronic

For peer review only

data collection with additional supervision in the DeWorm3 project, comprised the majority of unit costs (approximately 60%).

On average, SBD unit costs were $2.47 in Benin, $0.72 in India, and $1.69 in Malawi. Routine financial costs, such as per-diems, fuel, and materials, were approximately 5-30% of unit costs and were more expensive in Benin and Malawi ($0.53 and $0.48 respectively) as compared to India ($0.03). Routine opportunity costs, mainly teachers and school-level staff time, represented the largest share of costs in Benin and India (approximately 45% and 75% respectively); the governments of Benin and India were the primary SBD implementers. In Malawi, where SBD was delivered by the DeWorm3 team, routine opportunity costs were only 1/4 of costs whereas supportive activities represented half of unit costs.

Across sites, average unit costs were generally higher for cMDA as compared to SBD, except for Benin. However, routine cMDA costs were consistently less expensive compared to SBD, driven in part by the high opportunity costs of SBD. Across cMDA and SBD, program management and drug delivery were the most expensive activities. Drug delivery included initial drug distribution, as well as mop-up activities (approximately 10-20% of drug delivery costs). The largest resource input was staff wages and per-diems, representing 56%-91% of average unit costs, followed by vehicle costs (Appendix 5). Routine vehicle costs were used for government supervision and transport for training. However, the majority of vehicle costs were used for supportive activities, mainly field staff supervision and transport of enumerators to field sites each day for mobile data collection. Vehicle costs contributed to a higher share of costs in Malawi, compared to other sites. Approximately 15% of SBD and 25% cMDA costs were fixed or capital costs (Appendix 5), meaning the expenses do not depend on the quantity of treatments delivered. Examples of fixed costs include program overheads such as rent, central staff salaries, etc. When examining how unit costs per sub-activity varied across rounds, actual MDA delivery costs were the most variable across sites and rounds, followed by program management costs (Appendix 5). After planning costs, which were annualized across rounds, community sensitization showed the least amount of variability in unit costs across countries, rounds, and treatment strategies.

For peer review only

Table 2: Average unit costs (2019 USD ($)) for community-wide mass drug administration across two years
Subtitle: Total economic costs are presented, as well as a breakdown of costs by routine vs. supporting activities, and financial vs. opportunity costs

	Benin[a]	India[a]	Malawi[a]
**Planning**	**$ 0.10**	**$ 0.04**	**$ 0.01**
Supporting (financial)	$ 0.10	$ 0.04	$ 0.01
**Program management**	**$ 0.63**	**$ 0.40**	**$ 0.50**
Routine (financial)	$ 0.28	$ 0.16	$ 0.15
Routine (opportunity) – time costs for government staff[b]	$ 0.01	—	< $ 0.01
Supporting (financial)	$ 0.34	$ 0.24	$ 0.35
**Community sensitization**	**$ 0.24**	**$ 0.17**	**$ 0.17**
Routine (financial)	$ 0.11	$ 0.02	$ 0.06
Routine (opportunity) – time costs for government staff and volunteers	$ 0.01	< $ 0.01	$ 0.04
Supporting (financial) – additional sensitization activities	$ 0.01	< $ 0.01	$ 0.01
Supporting (financial) – NGO supervision	$ 0.11	$ 0.14	$ 0.06
**Training**	**$ 0.34**	**$ 0.11**	**$ 0.26**
Routine (financial)	$ 0.12	$ 0.01	$ 0.07
Routine (opportunity) – time costs for government staff and volunteers	$ 0.02	$ 0.03	$ 0.02
Supporting (financial) – training of electronic data collectors	$ 0.11	$ 0.05	$ 0.05
Supporting (financial) – NGO supervision and training support	$ 0.08	$ 0.02	$ 0.11
**Drug delivery**	**$ 1.13**	**$ 0.46**	**$ 1.32**
Routine (financial)	$ 0.36	$ 0.07	$ 0.20
Routine (opportunity) – time costs for government staff and volunteers	$ 0.15	$ 0.11	$ 0.18
Routine (opportunity) – donated drugs	$ 0.05	$ 0.01	$ 0.05
Supporting (financial) – electronic data capture	$ 0.29	$ 0.19	$ 0.31
Supporting (financial) – NGO supervision	$ 0.27	$ 0.07	$ 0.58
Average unit costs[c]	***$ 2.45***	***$ 1.17***	***$ 2.26***
Routine (financial)	*$ 0.87*	*$ 0.26*	*$ 0.48*
Routine (opportunity)	*$ 0.26*	*$ 0.16*	*$ 0.30*
Supporting (financial)	*$ 1.32*	*$ 0.75*	*$ 1.48*

Acronyms: non-governmental organization (NGO)
Note: Dashes (–) represent situations where no costs were observed.
[a]Analysis includes two years of cMDA. As cMDA was conducted bi-annually in each country, results are presented as the average across four rounds.
[b] Government staff include supervisory and implementing staff who's salaries are paid by the ministry of health. Examples include: nurses and health officers, HSAs (Malawi only), as well as national and subnational government officials involved in the program.
[c] Routine and supporting activities and related resources are described in Appendix 2. Financial costs represent actual expenditure on goods and services purchased by the government or NGO implementing partner. Opportunity costs, on the other hand, include costs forgone by using a resource in a particular way. These opportunity costs recognize and value the cost of using resources, as these resources are then unavailable for productive use elsewhere. Opportunity costs in this analysis include: costs of donated albendazole, volunteer time spent on the project (such as volunteer drug distributors), and estimated government staff salary costs.

Table 3: Average unit costs (2019 USD ($)) for school-based deworming across two years
Subtitle: Total economic costs are presented, as well as a breakdown of costs by routine program vs. supporting program activities, and financial vs. opportunity costs

	Benin [a]	India [b]	Malawi [a]
**Planning**	**$ 0.07**	**$ 0.00**	**$ 0.01**
Supporting (financial)	$ 0.07	—	$ 0.01
**Program management**	**$ 0.69**	**$ 0.19**	**$ 0.40**
Routine (financial)	—	—	$ 0.15
Routine (opportunity) – time costs for government staff [c]	$ 0.25	$ 0.11	$ 0.00
Supporting (financial)	$ 0.44	$ 0.08	$ 0.25
**Community sensitization**	**$ 0.26**	**$ 0.01**	**$ 0.11**
Routine (financial)	$ 0.14	$ 0.01	$ 0.04
Routine (opportunity) – time costs for government staff and volunteers	—	—	$ 0.05
Supporting (financial) – additional sensitization activities	$ 0.05	—	$ 0.01
Supporting (financial) – NGO supervision	$ 0.07	—	$ 0.02
**Training**	**$ 0.61**	**$ 0.18**	**$ 0.25**
Routine (financial)	$ 0.27	$ 0.02	$ 0.08
Routine (opportunity) – time costs for government staff and volunteers	$ 0.20	$ 0.14	$ 0.11
Supporting (financial) – training of electronic data collectors	$ 0.06	$ 0.02	$ 0.02
Supporting (financial) – NGO supervision and training support	$ 0.08	$ 0.01	$ 0.04
**Drug delivery**	**$ 0.83**	**$ 0.33**	**$ 0.91**
Routine (financial)	$ 0.12	$ 0.01	$ 0.22
Routine (opportunity) – time costs for government staff and volunteers	$ 0.56	$ 0.28	$ 0.17
Routine (opportunity) – donated drugs	$ 0.06	$ 0.01	$ 0.06
Supporting (financial) – electronic data capture	$ 0.02	$ 0.02	$ 0.21
Supporting (financial) – NGO supervision	$ 0.07	$ 0.01	$ 0.25
***Average unit costs* [d]**	***$ 2.47***	***$ 0.72***	***$ 1.69***
Routine (financial)	*$ 0.53*	*$ 0.03*	*$ 0.48*
Routine (opportunity)	*$ 1.07*	*$ 0.54*	*$ 0.40*
Supporting (financial)	*$ 0.87*	*$ 0.14*	*$ 0.81*

Acronyms: non-governmental organization (NGO)
Note: Dashes (–) represent situations where no costs were observed.
[a] Analysis includes two years of SBD. In India, SBD was conducted bi-annually, so results are presented as the average across four rounds.
[b] Analysis includes two years of SBD. In Malawi and Benin, SBD was conducted annually, so results are presented as the average of two rounds.
[c] Government staff include supervisory and implementing staff whose salaries are paid by the ministry of health. Examples include: nurses and health officers, teachers, and national and subnational government officials involved in the program.
[d] Routine and supporting activities and related resources are described in Appendix 2. Financial costs represent actual expenditure on goods and services purchased by the government or NGO implementing partner. Opportunity costs, on the other hand, include costs forgone by using a resource in a particular way. These opportunity costs recognize and value the cost of using resources, as these resources are then unavailable for productive use elsewhere. Opportunity costs in this analysis include: costs of donated albendazole, volunteer time spent on the project (such as volunteer drug distributors), and estimated government staff salary costs.

For peer review only

Additional programmatic costs

Costs of additional activities, such as an annual census, prevalence surveys, and coverage surveys are not included within cMDA and SBD unit cost estimates but are detailed in Appendix 5. In brief, costs of annual censuses ranged from $0.54 (India year 2) to $1.81 (Benin year 1) per person censused. Annual prevalence surveys where stool samples were analyzed using Kato-Katz ranged from $11.98 (India year 1) to $28.78 per person surveyed (India year 2); variability in costs was due to cross-country differences and shared laboratory costs in year 1 of the survey. Lastly, coverage surveys conducted post-MDA were estimated between $1.33 (India year 1) to $4.64 (Benin year 1) per person surveyed.

Sensitivity analyses

In one-way and two-way sensitivity analyses (Figure 2), the largest changes in cMDA and SBD unit costs were driven by altering coverage rates and supportive costs. Changing coverage rates in Malawi resulted in the largest change in estimated unit costs. Estimated deworming program coverage rates varied widely across clusters in Malawi (from 19-74% for SBD and 64-96% for cMDA), resulting in unit costs ranging from $1.26 to $4.91 per SBD treatment administered and $1.93 to $2.87 per cMDA treatment administered. Costs decreased in two-way analyses when supportive costs were removed and coverage rates were reduced to approximately 60% cMDA and SBD coverage (assuming that a reduction in support would result in a reduction in coverage); unit costs decreased by 30% or more in most cases. In these sensitivity analyses, the cost of cMDA and SBD became similar, with a net difference of $0.03 to $0.17. Unit costs did not fluctuate substantially in one-way sensitivity analyses exploring opportunity costs of drugs and volunteer time, and two-way sensitivity analyses exploring coverage and sensitization costs.

DISCUSSION

Costs and resource needs are important pieces of evidence for governments considering updating standards of care and related policies, such as a potential shift from SBD to cMDA. The DeWorm3 project provided a unique platform to assess and compare the costs of two deworming treatment strategies (SBD and cMDA) across heterogenous STH-endemic settings. We found the average unit cost per treatment administered to be higher for cMDA compared to SBD in India and Malawi, and comparable in Benin.

Costs of MDA for NTDs, including deworming, vary considerably in the literature, depending on treatment strategy, resources accounted for, and perspective. In a review of 34 studies of MDA costs, financial unit costs (excluding medicine) ranged from $0.01 to $8.50.[18] Typically, financial costs for STH SBD are estimated at less than $0.50 per treatment administered.[5] Our SBD routine financial costs align with these

For peer review only

estimates, however, our total economic costs are generally higher, due to the inclusion of planning costs, opportunity costs for teachers and other government staff, and supportive supervision and data collection activities. Few STH cMDA costs are available in the literature. The TUMIKIA study in Kenya estimated total program costs of bi-annual cMDA at $0.76 per treatment administered and routine program costs at $0.48.[12] These routine cost estimates are similar to DeWorm3 routine costs in India ($0.42), though are lower than routine costs in Malawi ($0.78) and Benin ($1.13). Our cMDA unit costs are comparable to other studies evaluating economic costs of cMDA for NTDs, such as trachoma costs (estimated at $1.53, excluding costs of antibiotics) and LF costs (ranging from $0.40 to $5.87, including drug costs).[13-15]

This study disaggregates routine program costs from supportive costs that are used to increase coverage (additional sensitization, NGO supervision, and electronic data collection). Average routine costs of cMDA were lower than SBD costs across countries. This is largely driven by salary costs for teachers and school directors who generally spend 1-3 days each year supportive SBD. Similar findings were observed in Niger, where deworming was delivered via SBD to children and via community-based treatment to children and at-risk adults (at fixed locations or their homes); unit costs of SBD were higher at $0.76 compared to $0.46 for community treatment. Differences in costs in Niger were attributed to CDDs treating more individuals than teachers.[16] Our results demonstrated that wages, per-diems, and opportunity costs (ex. time costs) for staff represented the largest share of total costs, a finding that was consistent across all sites and both implementation strategies. Similarly, the TUMIKIA trial found 67.5% of cMDA costs for STH in Kenya were financial and opportunity costs for personnel.[12] These findings highlight the importance of fully accounting for costs associated with the delivery workforce, including teachers involved in SBD and volunteer drug distributors in cMDA.

As it is not possible to disentangle the precise impact of supportive activities on coverage, sensitivity analyses were conducted to explore the potential impact of reducing supportive activities on unit costs. If supportive activities were removed and coverage reduced as a result, unit costs were estimated to drop between 10-50%. Although opportunities for electronic data collection during MDA are increasing (ex. ESPEN Collect), not all programs may choose to proceed with more resource-intensive mobile data collection.[17] However, evidence suggests high coverage of cMDA may be necessary to interrupt transmission, and thus the total costs presented in this study may be representative of costs incurred by elimination programs.

Given the experimental nature of cMDA and the DeWorm3 platform upon which it was implemented, cMDA costs may vary if launched within routine health systems. Depending on existing capacity within

For peer review only - http://bmjopen.bmj.com/site/about/guidelines.xhtml

countries, governments could see a reduction in costs due to cost-sharing between other community-based or NTD programs. Additionally, studies suggest that MDA costs are subject to economies of scale; according to one model, a 10-fold increase in individuals treated could reduce costs by approximately 70% in DeWorm3 countries.[11,18] Costs of cMDA collected over the first two years of implementation in DeWorm3 may have been high due to start-up costs, and therefore costs could reduce over time with experience, as observed in Haiti's integrated STH and lymphatic filariasis MDA program, which saw a decrease in cost per person treated from $2.23 during the first year of implementation in 2000, to $0.64 per person between 2008-2009.[15] Future modeled analyses of DeWorm3 costing data will explore costs of scaling cMDA programs, altering frequencies and sampling strategies for conducting additional program activities (e.g., censuses, prevalence surveys, and coverage surveys), and examining implications on drug costs if cMDA for STH were to be scaled up widely.

When examining average unit costs of cMDA and SBD across sites, we observed lowest costs in India, followed by Malawi, and Benin respectively. However, this pattern was not consistent when examining costs per round, by sub-activity, or by routine vs. opportunity cost. For example, unit costs of cMDA were highest in Malawi during rounds 1 and 2. Our results suggest unit costs of planning, training and community sensitization may be more similar across MDA treatment strategies and countries, while resources such as staffing and supervision for program management and drug delivery may be more setting specific. We briefly highlight several reasons for variation in unit costs across sites and a more extensive description of drivers of variation can be found in Appendix 6. Sites varied in the degree of NGO and government involvement. In Benin, the DeWorm3 team and the Ministry of Health worked closely together to implement cMDA and SBD. In Malawi, the DeWorm3 team led the implementation of both cMDA and SBD with supervisory support from the Government of Malawi. This close collaboration on implementation in Benin and Malawi incurred more allowances and opportunity costs for both "supporting" NGO staff and "routine" government staff. In India, there was a greater separation of responsibilities for cMDA and SBD, with the DeWorm3 team implementing cMDA and the Government of India implementing SBD. Given SBD was solely led by the Government of India, "supporting" costs were substantially lower. A driver of heterogeneity in SBD costs was variation in school staff involvement across sites. Opportunity costs for school staff were higher in India and Benin given a larger number of school staff such as teachers, Anganwadi Workers, and school directors involved, and higher salaries for school staff. Lastly, the different number of treatments administered, due to population sizes, population age-compositions, and coverage rates, affected unit costs. For example, total costs of SBD were similar in Benin and Malawi, however, more school-aged children were treated in Malawi resulting in lower unit costs. Previous studies have similarly reported differences in unit costs across countries, and wide intra-country variation. The

For peer review only

TUMIKIA study reported average unit costs of biannual cMDA in Kenya varied from $0.49—$1.85 across clusters.[10] Additionally, during nationwide scale-up of SBD in Uganda, costs varied $0.41—$0.91 across districts, given differences in number of children treated, community sensitization costs, and district-level supervision.[11] The inter- and intra-country variations highlight the many ways STH treatment strategies can be implemented, and how community-based health campaigns may need to be adjusted to adapt to specific population needs. We encourage future STH MDA costing studies to report details of implementation costs and to explore drivers of variation in costs and coverage within and across countries.

In addition to unit costs, other metrics should be considered to determine the relative value of cMDA and SBD. Cost-effectiveness analyses are important to compare costs to health benefits gained. If more children are treated through cMDA than SBD, and/or overall STH prevalence is reduced, costs per infection-year averted may be lower for cMDA compared to SBD.[12] If cMDA interrupts STH transmission, the long-term reduction in STH burden as a result of cMDA could be substantial. After DeWorm3 trial results are unblinded, further analyses will determine the incremental cost-effectiveness of cMDA compared to SBD under multiple time horizons to account for the long-term benefits of elimination.

There are several limitations to this analysis. As described above, there were different degrees of DeWorm3 involvement in SBD across sites; data sources and some driving assumptions thus necessarily varied. Although DeWorm3 trial costs were excluded from this costing analysis, we anticipate that program management, planning, and supervision costs may be higher than what would be observed routinely. Other assumptions are described in detail in Appendix 2.

CONCLUSION

This study provides evidence from a large micro-costing study, over twelve rounds of cMDA and eight rounds of SBD in Benin, India, and Malawi DeWorm3 sites. To our knowledge, this is the first study to directly compare costs of SBD to cMDA for STH programs.[5] On average, cost per treatment administered through cMDA was more expensive than SBD in India and Malawi, and comparable in Benin. The largest difference in sub-activity costs was related to drug delivery, where cMDA financial costs for routine resources (e.g. CDD allowances) and supporting activities (e.g. additional supervision) were notably higher than for SBD across all three countries. Although financial costs were higher for cMDA, opportunity costs for government-funded staff and volunteers were higher for SBD, mainly driven by teacher time. Overall, wages and per-diems represented the largest share of costs across countries and treatment strategies. Program planners should consider what changes in staffing and other resources are needed to implement

For peer review only

cMDA at scale, knowing that costs may vary given cross-country differences and economies of scale. Future budget-impact and cost-effective analyses will generate additional evidence on the value for money and affordability of cMDA compared to SBD.

DATA AVAILABILITY STATEMENT

Data collected from this study, including resources used, costs incurred, and the number of albendazole treatments administered, and a corresponding data dictionary will be made available upon reasonable request to the corresponding author. For inquiries, contact Dr. Arianna R. Means at aerubin@uw.edu.

RESEARCH ETHICS STATEMENT

This study was affiliated with a trial that was reviewed and approved by the Institut de Recherche Clinique au Bénin (IRCB) through the National Ethics Committee for Health Research (002-2017/CNERS-MS) from the Ministry of Health in Benin, The London School of Hygiene and Tropical Medicine (12013), The College of Medicine Research Ethics Committee (P.04/17/2161) in Malawi, and the Institutional Review Board at Christian Medical College, Vellore (10392). The DeWorm3 Project was also approved by the University of Washington (STUDY00000180).

ACKNOWLEDGEMENTS

The authors wish to thank all of the study participants, communities and community leaders, national NTD program staff, and local, regional, and national partners (Ministry of Health and Family Welfare, Delhi and the Directorate of Public Health, Chennai in India and Programme National de lutte contre les Maladies Transmissibles du Ministère de la Santé du Bénin) who have participated in or supported the implementation of the DeWorm3 study. We also would like to acknowledge the work of all members of the DeWorm3 study teams and affiliated institutions. We give special thanks to Dr. Kumudha Aruldas, Monrenike Bada, Miranda Benson, Chikondi Chikotichalera, Dr. Sobana Devavaram, Sean Galagan, Katherine Halliday, Noel Joyce Mary Hillari, Hugo Legge, Providence Nindi, Chinnaduraipandi Paulsamy, Emily Pearman, Rajeshkumar Rajendiran, Sarah Smith and Elodie Yard for assisting with data collection, cleaning, and interpretation.

For peer review only

AUTHOR CONTRIBUTIONS

KG, MS, KK, AL, MI, RB, RP, SSRA, JLW, and ARM conceptualized the project and methodology; JLW secured the funding. EA, SPK, JS, SPG, WL, and PH collected the data. EA, SPK, JS, SPG, WL, KG, MS, and CM curated the data. KG, MS, and CM analyzed the data. EA and MI were involved in the validation and interpretation of results from Benin; SPK and SPG were involved in validation and interpretation of results from India; JS, WL, and KK were involved in validation and interpretation of results from Malawi. ARM and CM drafted the manuscript and all authors revised and approved the manuscript for content.

COMPETING INTERESTS

The authors of this study declare no competing interests.

FUNDING

This work was funded by the Bill & Melinda Gates Foundation (INV-022149). The funders had no role in study design, data collection, data analysis, data interpretation, or writing of the article. We received no compensation from pharmaceutical companies or other agencies to write this article.

REFERENCES

1 Pullan RL, Smith JL, Jasrasaria R, et al. Global numbers of infection and disease burden of soil transmitted helminth infections in 2010. *Parasit Vectors* 2014; **7**: 37.

2 World Health Organization. Guideline: Preventive chemotherapy to control soil-transmitted helminth infections in at-risk population groups. Geneva, Switzerland: World Health Organization, 2017 http://www.who.int/nutrition/publications/guidelines/deworming/en/ (accessed Jan 5, 2021).

3 World Health Organization. Ending the neglect to attain the sustainable development goals: a road map for neglected tropical diseases 2021–2030: overview. World Health Organization, 2020 https://apps.who.int/iris/handle/10665/332094.

4 London Declaration on Neglected Tropical Diseases. Uniting to Combat Neglected Tropical Diseases, 2012 https://unitingtocombatntds.org.

For peer review only

5 Turner HC, Truscott JE, Hollingsworth TD, et al. Cost and cost-effectiveness of soil-transmitted helminth treatment programmes: systematic review and research needs. *Parasit Vectors* 2015; **8**. DOI:10.1186/s13071-015-0885-3.

6 Jia T-W, Melville S, Utzinger J, et al. Soil-transmitted helminth reinfection after drug treatment: a systematic review and meta-analysis. *PLoS Negl Trop Dis* 2012; **6**: e1621.

7 Anderson R, Truscott J, Hollingsworth TD. The coverage and frequency of mass drug administration required to eliminate persistent transmission of soil-transmitted helminths. *Philos Trans R Soc B Biol Sci* 2014; **369**: 20130435.

8 Ásbjörnsdóttir KH, Ajjampur SSR, Anderson RM, et al. Assessing the feasibility of interrupting the transmission of soil-transmitted helminths through mass drug administration: The DeWorm3 cluster randomized trial protocol. *PLoS Negl Trop Dis* 2018; **12**: e0006166.

9 Brooker SJ, Nikolay B, Balabanova D, et al. Global feasibility assessment of interrupting the transmission of soil-transmitted helminths: a statistical modelling study. *Lancet Infect Dis* 2015; **15**: 941–50.

10 Pullan RL, Halliday KE, Oswald WE, et al. Effects, equity, and cost of school-based and community-wide treatment strategies for soil-transmitted helminths in Kenya: a cluster-randomised controlled trial. *The Lancet* 2019; **393**: 2039–50.

11 Brooker S, Kabatereine N, Fleming F, et al. Cost and cost-effectiveness of nationwide school-based helminth control in Uganda. *Health Policy Plan* 2008; **23**: 24–35.

12 Turner HC, Bundy DAP. Programmatic implications of the TUMIKIA trial on community-wide treatment for soil-transmitted helminths: further health economic analyses needed before a change in policy. *Parasit Vectors* 2020; **13**: 102.

13 Kolaczinski JH, Robinson E, Finn TP. The Cost of Antibiotic Mass Drug Administration for Trachoma Control in a Remote Area of South Sudan. *PLoS Negl Trop Dis* 2011; **5**: e1362.

14 Goldman AS, Guisinger VH, Aikins M, et al. National Mass Drug Administration Costs for Lymphatic Filariasis Elimination. *PLoS Negl Trop Dis* 2007; **1**. DOI:10.1371/journal.pntd.0000067.

15 Goldman AS, Brady MA, Direny A, et al. Costs of Integrated Mass Drug Administration for Neglected Tropical Diseases in Haiti. *Am J Trop Med Hyg* 2011; **85**: 826–33.

For peer review only

16 Leslie J, Garba A, Oliva EB, et al. Schistosomiais and Soil-Transmitted Helminth Control in Niger: Cost Effectiveness of School Based and Community Distributed Mass Drug Administration. *PLoS Negl Trop Dis* 2011; **5**. DOI:10.1371/journal.pntd.0001326.

17 Expanded Special Project for Elimination of Neglected Tropical Diseases (ESPEN). ESPEN Collect. https://espen.afro.who.int/tools-resources/espen-collect (accessed May 25, 2021).

18 Fitzpatrick C, Fleming FM, Madin-Warburton M, et al. Benchmarking the Cost per Person of Mass Treatment for Selected Neglected Tropical Diseases: An Approach Based on Literature Review and Meta-regression with Web-Based Software Application. *PLoS Negl Trop Dis* 2016; **10**: e0005037.

19 Adamiak G. Methods for the economic evaluation of health care programmes, 3rd ed. *J Epidemiol Community Health* 2006; **60**: 822–3.

20 Claxton K, Revill P, Sculpher M, et al. The Gates Reference Case for Economic Evaluation. The Bill and Melinda Gates Foundation, 2014.

21 Galactionova K, Sahu M, Gideon S, et al. Costing interventions in the field: preliminary cost estimates and lessons from an evaluation of community-wide Mass Drug Administration for elimination of Soil-Transmitted Helminths in the DeWorm3 trial. *BMJ Open* 2021; **11**: e0409734

22 Means AR, Ajjampur SSR, Bailey R, et al. Evaluating the sustainability, scalability, and replicability of an STH transmission interruption intervention: The DeWorm3 implementation science protocol. *PLoS Negl Trop Dis* 2018; **12**: e0005988.

23 World Bank. Official exchange rate (LCU per US$, period average). https://data.worldbank.org/indicator/PA.NUS.FCRF (accessed Jan 27, 2021).

24 Anna Vassall, Sedona Sweeney, Jim Kahn, et al. Reference Case for Estimating the Costs of Global Health Services and Interventions. Global Health Cost Consortium, 2017 https://ghcosting.org/pages/standards/reference_case (accessed June 16, 2021).

25 GDP deflator (base year varies by country) | Data. https://data.worldbank.org/indicator/NY.GDP.DEFL.ZS (accessed June 16, 2021).

26 Turner HC, Lauer JA, Tran BX, et al. Adjusting for Inflation and Currency Changes Within Health Economic Studies. *Value Health* 2019; **22**: 1026–32.

For peer review only

27 Tamil Nadu State Government Department of Evaluation and Applied Research. Tamil Nadu - An Economic Appraisal 2011-12 to 2013-14.

28 International Labor Organization. ILOSTAT database. https://ilostat.ilo.org/data (accessed Aug 25, 2021).

FIGURES

Figure 1: Flow of DeWorm3 activities conducted in intervention and control clusters.
Activities include: census, prevalence survey, school-based deworming, community-wide mass drug administration, and coverage survey

Footnotes:

Acronyms: quarter (Q), school-based deworming (SBD), community-wide mass drug administration (cMDA).

[a] In India, SBD is also conducted in quarter 2, prior to the coverage survey.

[b] In Malawi, no prevalence survey was conducted in year 2.

Figure 2: One- and two-way sensitivity analyses of unit costs (2019 USD ($)).
a) community-wide mass drug administration (cMDA) costs in Benin; b) school-based deworming (SBD) costs in Benin; c) cMDA costs in India; d) SBD costs in India; e) cMDA costs in Malawi; f) SBD costs in Malawi. Details on how each parameter was varied can be found in Appendix 4.

For peer review only

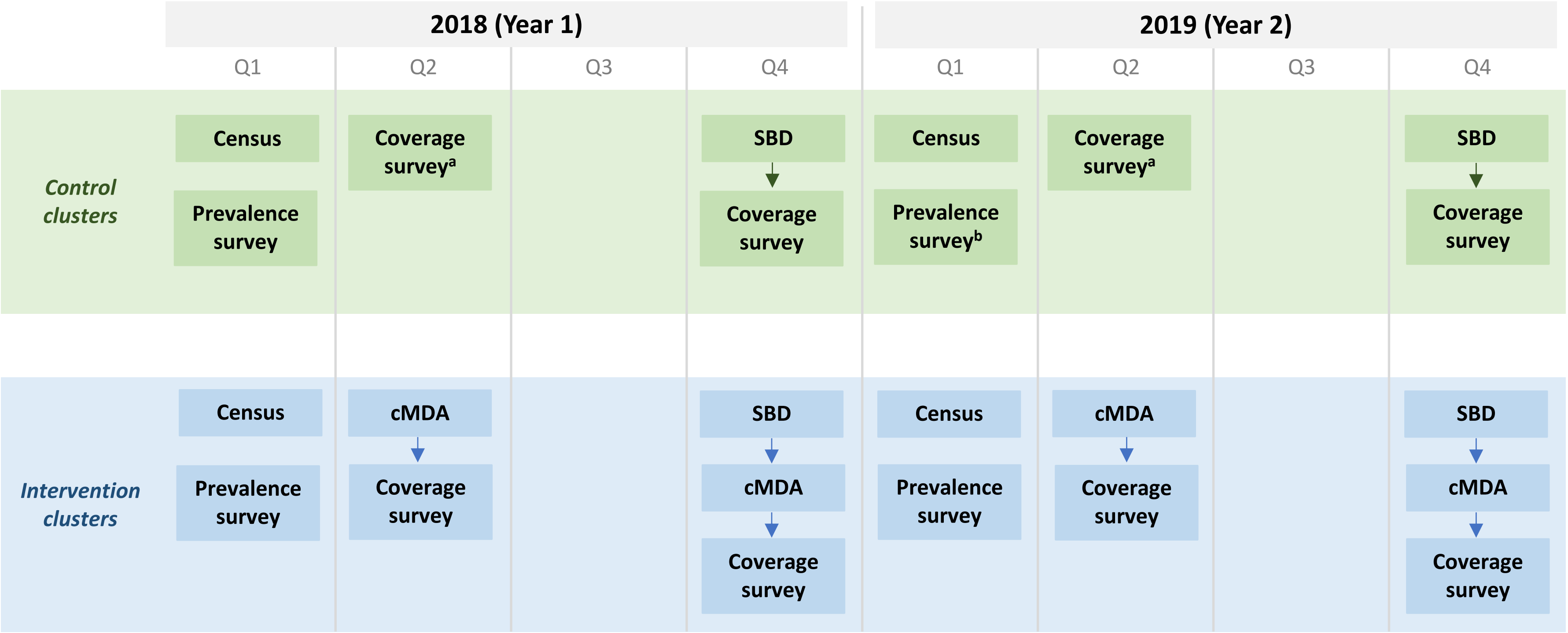
2018 (Year 1)
2019 (Year 2)
Q1
Q2
Q3
Q4
Q1
Q2
Q3
Q4
Control clusters
Census
Prevalence survey
Coverage survey[a]
SBD
Coverage survey
Census
Prevalence survey[b]
Coverage survey[a]
SBD
Coverage survey
Intervention clusters
Census
Prevalence survey
cMDA
Coverage survey
SBD
cMDA
Coverage survey
Census
Prevalence survey
cMDA
Coverage survey
SBD
cMDA
Coverage survey

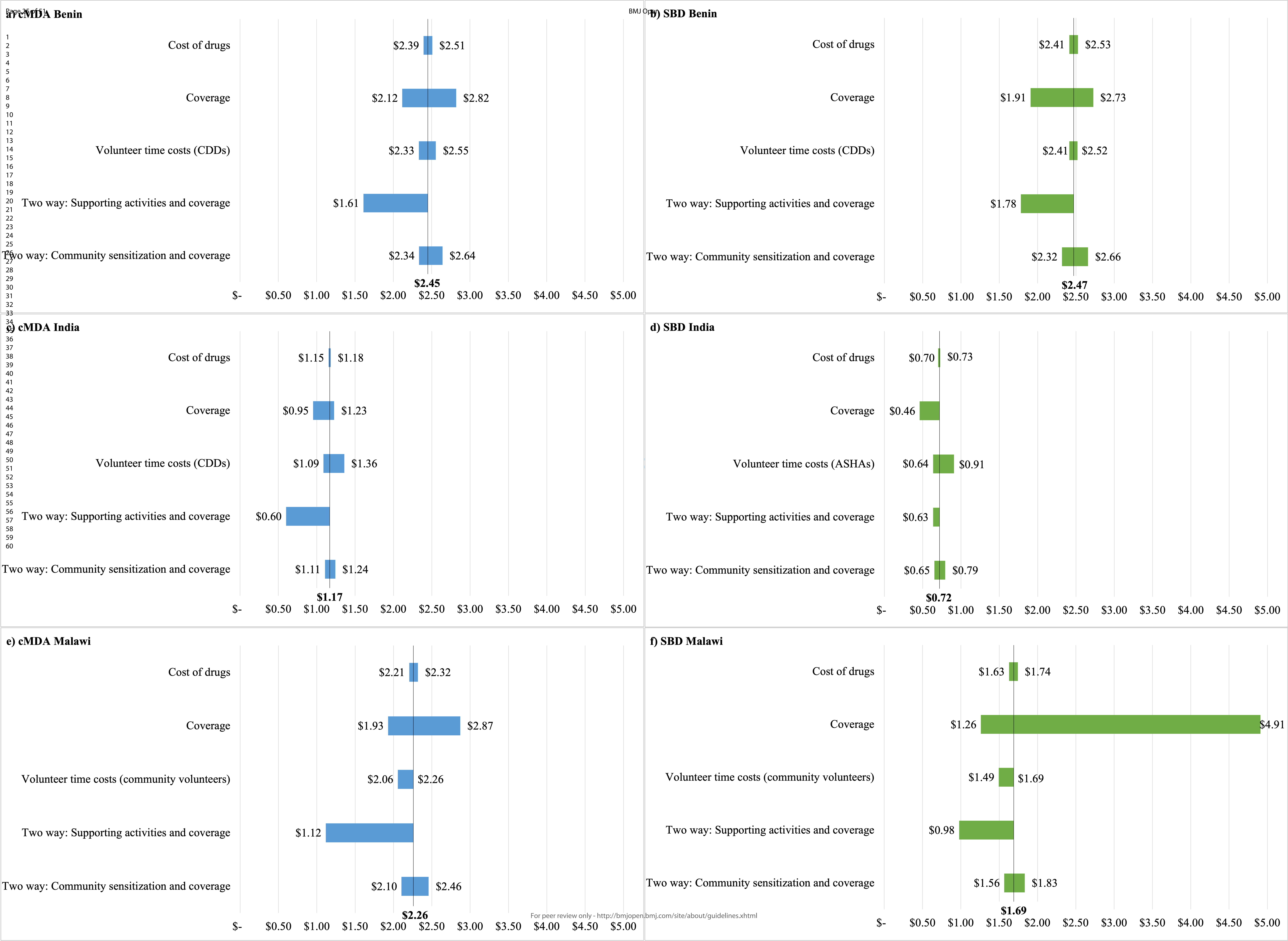
a) cMDA Benin
Cost of drugs
$2.39
$2.51
Coverage
$2.12
$2.82
Volunteer time costs (CDDs)
$2.33
$2.55
Two way: Supporting activities and coverage
$1.61
Two way: Community sensitization and coverage
$2.34
$2.64
$2.45
$-
$0.50
$1.00
$1.50
$2.00
$2.50
$3.00
$3.50
$4.00
$4.50
$5.00
b) SBD Benin
Cost of drugs
$2.41
$2.53
Coverage
$1.91
$2.73
Volunteer time costs (CDDs)
$2.41
$2.52
Two way: Supporting activities and coverage
$1.78
Two way: Community sensitization and coverage
$2.32
$2.66
$2.47
c) cMDA India
Cost of drugs
$1.15
$1.18
Coverage
$0.95
$1.23
Volunteer time costs (CDDs)
$1.09
$1.36
Two way: Supporting activities and coverage
$0.60
Two way: Community sensitization and coverage
$1.11
$1.24
$1.17
d) SBD India
Cost of drugs
$0.70
$0.73
Coverage
$0.46
Volunteer time costs (ASHAs)
$0.64
$0.91
Two way: Supporting activities and coverage
$0.63
Two way: Community sensitization and coverage
$0.65
$0.79
$0.72
e) cMDA Malawi
Cost of drugs
$2.21
$2.32
Coverage
$1.93
$2.87
Volunteer time costs (community volunteers)
$2.06
$2.26
Two way: Supporting activities and coverage
$1.12
Two way: Community sensitization and coverage
$2.10
$2.46
$2.26
f) SBD Malawi
Cost of drugs
$1.63
$1.74
Coverage
$1.26
$4.91
Volunteer time costs (community volunteers)
$1.49
$1.69
Two way: Supporting activities and coverage
$0.98
Two way: Community sensitization and coverage
$1.56
$1.83
$1.69

APPENDIX

Costs of community-wide mass drug administration and school-based deworming for soil-transmitted helminths: evidence from a randomized-controlled trial in Benin, India, and Malawi

TABLE OF CONTENTS

Appendix 1: Additional details on DeWorm3 activities implemented 2

Appendix 1: Table 1: Implementation characteristics of the DeWorm3 trial at study sites....2

Appendix 1: Table 2: Narrative description of DeWorm3 mass drug administration activities....3

Appendix 2: Additional details on costing methodology....6

Appendix 2: Table 1: DeWorm3 instruments for cost collection 6

Appendix 2: Table 2: Summary of resources included in community-wide mass drug administration and school-based deworming costing analyses, by routine and supporting program costs 7

Appendix 2: Table 3: Key costing inputs (non-exhaustive) for community-wide mass drug administration and school-based deworming, per country....9

Appendix 2: Table 4: Key assumptions regarding unit cost analysis for community-wide mass drug administration and school-based deworming....11

Appendix 3: Costs in local currency 13

Appendix 3: Table 1: Total economic costs and number of treatments administered through community-wide mass drug administration and school-based deworming, per country-round, in local currency....13

Appendix 3: Table 2: Average unit costs (2019 local currency) for community-wide mass drug administration across two years...14

Appendix 3: Table 3: Average unit costs (2019 local currency) for school-based deworming across two years 15

Appendix 3: Table 4: Annual costs of additional deworming program activities, including censuses, prevalence surveys, and coverage surveys, across two years of implementation (2019 local currency)....16

Appendix 4: Additional details of sensitivity analysis methodology 17

Appendix 5: Additional costing results....18

Appendix 5: Table 1: Percentage of DeWorm3 activity unit costs that are fixed vs. variable, by activity and country....18

Appendix 5: Figure 1: Total financial and opportunity costs of a) community-wide mass drug administration and b) school-based deworming by input-classification (including routine and supporting program costs). 19

Appendix 5: Table 2: Community-wide mass drug administration costs by input classification, by routine vs. supporting costs....20

Appendix 5: Table 3: School-based deworming costs by input classification, by routine vs. supporting costs....20

Appendix 5: Figure 2: Mean and range of unit costs per sub-activity across four rounds of community-wide mass drug administration in Benin, India, and Malawi; two rounds of school-based deworming in Benin and Malawi; and, four rounds of school-based deworming in India....21

Appendix 5: Table 4: Annual costs of additional deworming program activities, including censuses, prevalence surveys and coverage surveys, across two years of implementation (2019 USD ($))....22

Appendix 6: Description of cost differences across countries 23

Appendix 6: Table 1: Drivers of heterogeneity in costs across sites....23

Appendix 7: Appendix references....25

For peer review only

Appendix 1: Additional details on DeWorm3 activities implemented

In the below table, we provide additional details on how community-wide mass drug administration (cMDA) and school-based deworming (SBD) were implemented in each country.

Appendix 1: Table 1: Implementation characteristics of the DeWorm3 trial at study sites

	Benin	India	Malawi
Study location	Come Commune	Tamil Nadu State (Vellore and Thiruvanamalai districts)	Mangochi District
Implementing organizations	• Institut de Recherche Clinique du Benin<br>• Institut de Recherche pour le Développement<br>• Ministry of Health, Benin	• Christian Medical College, Vellore<br>• Ministry of Health and Family Welfare, New Delhi and Directorate of Public Health, Chennai	• Blantyre Institute for Community Outreach<br>• London School of Tropical Medicine and Hygiene<br>• Ministry of Health and Education, Malawi
cMDA strategy (20 intervention clusters)	• Bi-annual cMDA in all ages.<br>• Community drug distributors (volunteers) delivered drugs<br>• Implemented by DeWorm3	• Bi-annual cMDA in all ages; following National Deworming Day (described below)<br>• Community drug distributors (volunteers) delivered drugs<br>• Implemented by DeWorm3	• Bi-annual cMDA in all ages<br>• Health Surveillance Assistants, employed by the government, delivered drugs<br>• Implemented by DeWorm3
SBD strategy (20 intervention clusters and 20 control clusters)[a]	• SBD conducted annually<br>• Treatment of children 5-14 years old<br>• Implemented by the Ministry of Health (with a subcontract from DeWorm3)	• National Deworming Days, conducted bi-annually in schools and Anganwadi centers (pre-schools)<br>• Treatment of children 1-19 years old<br>• Implemented by the Ministry of Health and Family Welfare, New Delhi and Directorate of Public Health, Chennai	• SBD conducted annually, integrated with "Child Health Days"<br>• Community mop-up for non-enrolled children<br>• Treatment of children 1-14 years old<br>• Implemented by DeWorm3
Additional trial activities	• Planning meetings<br>• Annual census<br>• Two prevalence surveys<br>• Bi-annual coverage survey (after each round of MDA	• Planning meetings<br>• Annual census<br>• Two prevalence surveys<br>• Bi-annual coverage survey	• Planning meetings<br>• Annual census<br>• One prevalence survey Bi-annual coverage survey

Acronyms: mass drug administration (MDA), community-wide MDA (cMDA), school-based deworming (SBD)
[a] SBD was implemented in the entire Dw3 study area (40 clusters) per each country's national deworming strategy, however SBD was only costed in control clusters (n=20).

For peer review only - http://bmjopen.bmj.com/site/about/guidelines.xhtml

Appendix 1: Table 2: Narrative description of DeWorm3 mass drug administration activities

MALAWI		
**Sub-activity**	**Community-wide mass drug administration (cMDA)**	**School-based delivery (SBD)**
Supply chain	• *Shipment to country*: Drugs were donated, ordered through the WHO. One shipment for both cMDA and SBD was made for 1.5 million doses and sent by ship, which supplied all years of the project. The stock was kept at the Central Drug Stores in Lilongwe and then dispensed to the study.<br>• *Storage and disbursement*: Albendazole for each MDA round was stored in the Deworm3 office in Namwera. During cMDA, albendazole was dispensed daily to the enumerators, and the remaining stock was returned to the office each evening. Drug supply was monitored using stock control cards and excel files of stock issued to enumerators.	• *Shipment:* Same shipment as cMDA.<br>• *Storage and disbursement*: Albendazole for each MDA round was stored in the Deworm3 office in Namwera. Field officers (employed by DeWorm3) transported the drugs between the office and schools during SBD.
Sensitization	Several committees and community boards were engaged for MDA sensitization. In year 2, the DeWorm3 team employed additional sensitization measures to improve community engagement and maximize treatment coverage. Activities included:<br>• Area Development Council meetings with group village headmen and/or representatives from Village Development Committees.<br>• Village level community meetings were conducted by Health Surveillance Associates (HSAs) and volunteers.<br>• Village dramas and public announcements (year 2 only).<br>• Religious and Traditional Authority leaders of the Community Advisory Board visited communities that displayed signs of community tension or low participation to resolve any communication issues (year 2 only).	Sensitization for SBD was combined with cMDA sensitization activities.
Training	• *Health staff and volunteers*: Field officers trained HSAs at health centers and halls. Training sessions were one day long, though they were conducted over the course of two days to accommodate all health center staff. Afterward, HSAs oriented volunteers.<br>• *Enumerators:* Trial coordinator and field officers trained enumerators for two days, followed by a three-day pilot of data collection instruments used during MDA.	• *Health staff and volunteers:* Training for SBD was combined with cMDA training activities.<br>• *Enumerators:* Training for SBD was combined with cMDA training activities.<br>• *Teachers and other school staff:* Training of teachers and principal education assistants was conducted by field officers supported by the Ministry of Health STH Programme Manager.
Drug delivery	Drug delivery was conducted twice per year, in intervention clusters only (n=20), by teams of enumerators, HSAs, and volunteers. HSAs were responsible for a relatively large number of households. HSAs supervised volunteers (about 4 volunteers per HSA). Enumerators were driven daily from Namwera to the community with their drug stocks, and HSAs were picked up along the way. Area Development Council members helped in mobilizing the community on the day of MDA.	School-based deworming was conducted once per year in all DeWorm3 clusters (n=40); in intervention clusters, SBD was conducted prior to cMDA. Treatment was administered at each school by the link HSA, with administrative support from two school teachers and the headteacher.<br><br>Children were also treated for schistosomiasis, using praziquantel. Costs of praziquantel were excluded from this costing analysis.
Supervision	Supervision was conducted by the DeWorm3 trial coordinator, DeWorm3 field officers, local health officers (Environmental Health Officers, Assistant Environmental Health Officers, District Environmental Health Officer, District Health Officer), District Council Representative, District STH Coordinator, and the Ministry of Health STH Programme Manager.	Supervision was conducted by the DeWorm3 trial coordinator, DeWorm3 field officers, local health officers (Environmental Health Officers, Assistant Environmental Health Officers, District Health Officer), District Council Representative, District STH Coordinator, Primary Education Authorities, and a representative from the Ministry of Education.
Mop-up	Malawi did not have a distinct mop-up period for cMDA. Instead, progress on coverage was tracked by a DeWorm3 monitoring dashboard, informed by electronic data collection forms. MDA was only considered complete once the dashboard indicated that all households had been treated or visited three times; all individuals who were absent from the household, but not migrated, at the first visit were followed up at least two further times.<br><br>Mop-up costs were estimated in the analysis as approximately 1-2 days of work, to indicate the individuals who were followed up with more than once.	Village level MDA of children who weren't in school was conducted as "mop-up" for two days after SBD.

For peer review only - http://bmjopen.bmj.com/site/about/guidelines.xhtml

INDIA		
**Sub-activity**	**Community-wide mass drug administration (cMDA)**	**School-based delivery (SBD)**
Supply chain	• *Shipment to country*: Drugs were donated, ordered through the WHO. Drugs were ordered centrally by the Ministry of Health and Family Welfare, through the national NTD program.<br>• *Storage and disbursement*: Consignment was brought to the central DeWorm3 office in Vellore, and delivered to two subsite field offices. Field supervisors managed the tablets and provided them to fieldworkers daily to take to the villages for community drug distributors (CDDs) to dispense. The remaining tablets were returned to the office at end of the day.	• *Shipment to country*: Same shipment as cMDA.<br>• *Storage and disbursement*: Consignment was brought to the central DeWorm3 office in Vellore, and delivered to two subsite field offices. Field supervisors managed the tablets and provided them to Village Head Nurses to supply all schools and Anganwadi Centers.
Sensitization	• National Deworming Day sensitization materials were adapted to include information on cMDA; 1000 posters and 200 banners were posted in villages.<br>• Community sensitization meetings were conducted by DeWorm3 field staff using locally designed flipbooks to explain how STH are transmitted and what activities would be undertaken during cMDA.	Cloth banners provided by the government were put up by school staff outside schools and Anganwadi Centers one day before SBD.
Training	• *Health staff and volunteers*: CDDs had a half-day training, conducted by the DeWorm3 medical officer.<br>• *Enumerators:* DeWorm3 fieldworkers were trained by the DeWorm3 trial coordinator and data manager, followed by a short pilot period to test forms used during MDA.	• *Health staff and volunteers:* Training for SBD was combined with cMDA training activities.<br>• *Enumerators:* Training for SBD was combined with cMDA training activities.<br>• *Teachers and other school staff:* Workshops were held for teachers, Anganwadi Workers, and Village Health Nurses at every primary health center, conducted by respective primary health center medical officers.
Drug delivery	Drug delivery was conducted twice per year, in intervention clusters only (n=20), by teams of DeWorm3 fieldworkers (serving as enumerators) and CDDs, who walked door to door in the community. Nurses and medical officers supported with adverse events.	School-based deworming (called National Deworming Day) was conducted twice per year in all DeWorm3 clusters (n=40); in intervention clusters, SBD was conducted prior to cMDA. Drugs were delivered by Village Health Nurses in schools and Anganwadi Centers. ASHA workers and volunteers provided support as needed. DeWorm3 fieldworkers attended to deliver ink pens and treatment summary sheets. Nurses and medical officers helped with adverse events.
Supervision	Supervision was conducted by DeWorm3 field supervisors, DeWorm3 field managers, and local health workers (Village Head Nurses, Sector Health Nurses, and Community Health Nurses, and Block Medical Officers).	Supervision was conducted by local health workers (Village Head Nurses, Sector Health Nurses, and Community Health Nurses, and Block Medical Officers), central and sub-national level government health authorities.
Mop-up	After cMDA, a mop-up campaign was conducted for 1-4 days to reach absent individuals. Homes with absent individuals were visited up to three times.	One additional day of mop-up was conducted for children who were absent at school on National Deworming Day.

For peer review only

BENIN		
Sub-activity	**Community-wide mass drug administration (cMDA)**	**School-based delivery (SBD)**
Supply chain	• *Shipment to country*: Drugs were donated, ordered through the WHO. Drugs were ordered centrally by the Ministry of Health, for routine use, and stored in the national storage facility.<br>• *Storage and disbursement:* Drugs were dispatched to the zonal referral hospital in Come, by the National Communicable Disease Control Program (Programme National de Lutte contre les Maladies Transmissibles or PNLMT). Afterward, drugs were transferred to each health center affiliated with DeWorm3, with transit supervised by head doctors at commune level. Nurses collected drugs for the MDA campaign from the referral hospital after training. Nurses then dispensed drugs to CDDs for cMDA. After cMDA, the remaining drugs were transported from clusters to the central level.	• *Shipment to country*: Same shipment as cMDA.<br>• *Storage and disbursement*: Same disbursement process as cMDA, except nurses dispensed drugs to school headmasters rather than to CDDs.
Sensitization	• Information sessions were held with local authorities (town hall), leaders of opinion, religious leaders, professional associations, and town criers.<br>• Messages were passed to the community through town criers, radio broadcasts, specific groups (i.e. women's associations), and religious centers.<br>• Banners and posters were also placed in the community.	• Sensitization for SBD was combined with cMDA sensitization activities.<br>• Additional activities included: a meeting with the chief of the pedagogical region and his advisors (Ministry of Education responsible for Come commune), sensitization of teachers via meetings (year 2), and flier distribution in schools.
Training	• *Health staff and volunteers:* Ministry of Health staff trained 10 head health personnel (health center nurses, Chief Medical Officer, and District Medical Coordinator). Head nurses then trained CDDs. Supervision of training was done by PNLMT technical staff, doctors, and some district and departmental level staff.<br>• *Enumerators:* DeWorm3 staff trained enumerators and controllers (supervisors of enumerators).	• *Health staff and volunteers:* Training was combined with cMDA.<br>• *Teachers and school staff:* Ministry of Education officials and school headmasters were trained by 4 PNLMT staff and 2 DeWorm3 staff.
Drug distribution	Drug delivery was conducted twice per year, in intervention clusters only (n=20). Drugs were distributed by CDDs, joined by an enumerator, with the assumption that each CDD/enumerator pair would treat 60 people per day.	School-based deworming was conducted once per year in all DeWorm3 clusters (n=40); in intervention clusters, SBD was conducted prior to cMDA. Teachers administered drugs to children attending school. School directors/headmasters supervised and reported. CDDs treated non-enrolled children, who were invited to go to the closest school. Enumerators observed and fill out a treatment register.
Supervision	Supervision was conducted by DeWorm3 staff, central PNLMT staff, departmental staff, District Chief Doctors, and sub-district health center nurses.	The same supervisory staff as cMDA.
Mop-up	Two days of mop-up was conducted as needed. There was no mop-up in round 1 of cMDA. In round 4, flooding interrupted cMDA, and extensive mop-up was conducted.	No mop-up period.

Acronyms: World Health Organization (WHO), mass drug administration (MDA), community-wide mass drug administration (cMDA), school-based deworming (SBD), soil-transmitted helminths (STH), neglected tropical diseases (NTD), Programme National de Lutte contre les Maladies Transmissibles (PNLMT).

For peer review only - http://bmjopen.bmj.com/site/about/guidelines.xhtml

Appendix 2: Additional details on costing methodology

In the following tables, we provide additional details on the DeWorm3 costing methodology, including details on data collection tools and key model assumptions.

Appendix 2: Table 1: DeWorm3 instruments for cost collection

Source	Primary use	Type of cost	Content
DeWorm3 costing tool	Estimate resource use and costs of activities implemented by the DeWorm3 team	Financial and opportunity	Resource line items, corresponding prices, quantities, and expenditure recorded by sub-activity; separate modules for start-up and implementation
Activity table	Understand the purpose of resource use and how costs from the DeWorm3 costing tool relate to the implementation of activities	Financial and opportunity	Description of operational activities and sub-activities, number of project staff and other resources used, number of days
Activity calendar	Allocate shared costs to activities based on time spent on activities, such as staff salaries	N/A	Start, end dates, and duration of operational activities
Ministry of health costing tools	Estimate government costs of school-based deworming in DeWorm3 study area and Ministry of Health involvement in cMDA	Financial and opportunity	Budgets for routine school-based deworming at the national or state level across countries, government-funded employee salaries, and time spent on activities
MDA forms (i.e. digital treatment forms)	Estimate the number of persons treated, and time spent delivering treatments, to determine time spent by CDDs in each household	Opportunity costs, cost per person treated	Time spent per household to deliver community MDA; the number of persons treated
Census	Calculate relative DeWorm3 population size to district or state, in order to allocate district or state level costs to study area	N/A	DeWorm3 population size, control (SBD) and intervention (cMDA) cluster population size and demographic indicators such as age
School survey	Estimate teacher-related costs	Opportunity	Number of teachers trained, number of teachers involved in SBD, and time spent on activities
Literature	Collect relevant information where gaps persist	Financial and opportunity	District and state population sizes, number of schools per district/state, costs of equipment already owned

Acronyms: community-wide mass drug administration (cMDA), mass drug administration (MDA), school-based deworming (SBD), community drug distributor (CDD)

only

For peer review only - http://bmjopen.bmj.com/site/about/guidelines.xhtml

Appendix 2: Table 2: Summary of resources included in community-wide mass drug administration and school-based deworming costing analyses, by routine and supporting program costs

	Routine program costs	Supporting program costs
**Planning**		
Definition	None.	Start-up planning costs for DeWorm3, including developing IEC materials, mobile data collection forms, recruitment, and planning meetings with stakeholders.
Financial costs	None.	DeWorm3 salaries; travel, per-diems, and materials for planning meetings.
**Program management**		
Definition	Estimated operating costs to conduct routine program activities.	Estimated operating costs to conduct supporting program activities such as additional supervision and electronic data collection.
Financial costs	Salaries and overheads for DeWorm3 staff managing the project, including planning and reporting, building rent and utilities, equipment such as computers, vehicles, etc. Borrowed or pre-owned items, annualized across useful life years.	Same as routine program costs.
Opportunity costs	Time costs for government staff involved in the management of deworming programs.	None.
**Community sensitization**		
Definition	Sensitization activities varied across sites and also varied between school-based deworming and community-wide mass drug administration. For a complete list of activities conducted in each country, please see Appendix 1: Table 2. Examples include meetings with local committees/authorities/leaders, engagement with village chiefs, village dramas, door-to-door sensitization, posters and banners, radio advertisements, public criers.	Activities beyond those expected in routine programs, such as sensitizing the community to DeWorm3 research activities.
Financial costs	Per-diems and travel allowances, meeting costs such as refreshments and chair rentals, sensitization materials.	Examples include meeting costs for a Community Advisory Board, resources to hold a soccer competition/community event, and additional teacher sensitization.
Opportunity costs	Time costs for government-funded staff involved in sensitization (Health Surveillance Associates). Uncompensated time for volunteer staff who were involved in sensitization, such as community drug distributors. Time is valued using average national or regional salaries.	None.
**Training**		
Definition	Resources to train community drug distributors, volunteers, and health workers involved in drug delivery.	Resources to train enumerators involved in electronic data collection, as well as additional supervision by deworm3 implementing partners.
Financial costs	Per-diems, printed materials, refreshments, and hall rental.	Per-diems, printed materials, refreshments, and hall rental.

For peer review only - http://bmjopen.bmj.com/site/about/guidelines.xhtml

Opportunity costs	Time costs for government-funded staff involved in training (e.g., teachers, supervisors). Uncompensated time for volunteer staff who were trained, such as community drug distributors (Benin and India), ASHAs (India), and volunteers (Malawi). Time is valued using average national or regional salaries.	None.
**Drug delivery**		
Definition	Resources to deliver drugs either in the community or at schools, including mop-up.	Additional resources for enumerators to collect electronic monitoring data, and for supervision by deworm3 implementing partners.
Financial costs	Fuel, car rentals and per-diems for government supervisors, allowances/incentives for drug distributors, drugs for adverse events. Time for government supervisors, teachers.	Per-diems, mobile allowances for uploading data, fuel, and car hires.
Opportunity costs	Time costs for government-funded staff involved in drug delivery (e.g., teachers, supervisors). Uncompensated time for volunteer staff, such as community drug distributors (Benin and India), ASHAs (India), and volunteers (Malawi). Time is valued using average national or regional salaries. Costs of donated drugs.	None.

For peer review only

For peer review only - http://bmjopen.bmj.com/site/about/guidelines.xhtml

Appendix 2: Table 3: Key costing inputs (non-exhaustive) for community-wide mass drug administration and school-based deworming, per country

	Benin	India	Malawi	Data source
**DeWorm3 study site**				
Number villages	52	401	113	DeWorm3 Village Registry
Baseline population	94,969	140,932	121,819	DeWorm3 Census
**Days of drug delivery, including mop-up**				
cMDA: mean days (min-max)	12 (11—15)	13 (11—16)	16 (16)	DeWorm3 activity list
SBD: mean days (min-max)	2 (2)	2 (2)	5 (5)	DeWorm3 activity list
**Drug costs**				
Albendazole: opportunity cost, per tablet	$0.05	$0.01	$0.05	Benin and Malawi: GSK[1], India: National Deworming Day financial guidelines[a]
**Drug Distributors (CDDs and HSAs)**				
Staff involved: mean (min-max)	90 (90)	127 (114—164)	56 (56)	DeWorm3 costing tool
Monthly salary: approximate	$125	$126	$203	Benin: ILO[2]; India: State salary estimates[3], Malawi: DeWorm3 Ministry of Health costing survey
Time spent on cMDA training and sensitization: days	2	2	2	DeWorm3 costing tool
Time spent per cMDA visit, including travel: median minutes	17	11	14	DeWorm3 MDA forms
Number of cMDA visits conducted per drug distributor, per round: median visits	181	177	328	DeWorm3 MDA forms
Daily allowances[b] for drug delivery	$3.41	$3.55	$5.37	DeWorm3 costing tool
**Teachers**[c]				
Number of schools: median (min-max)	55 (54—55)	254 (228—298)	35 (29—40)	SBD forms
Teachers involved in SBD: mean (min-max)	304 (288—320)	339 (331—347)	147 (121—173)	DeWorm3 costing tool and school survey
Monthly salary: approximate[d]	$380	$456	$203	DeWorm3 Ministry of Health costing survey
Time spent on training and reporting: median days	0.25	0.625	0.5	Ministry of Health costing tool
Time spent on drug delivery: median days	0.33	0.25	1	DeWorm3 school survey
Allowances given	None	Per training	Per training, per day of drug delivery	Ministry of Health costing tool, DeWorm3 costing tool
Allowance rate	—	$1.42	$5.41	DeWorm3 costing tool
**Other school staff**[c]				
Position	School Directors	Anganwadi Workers	—	DeWorm3 costing tool and school survey
Number staff involved in SBD: mean (min-max)	55 (54—55)	126 (124—127)	—	DeWorm3 costing tool and school survey
Monthly salary: approximate	$539	$188	—	DeWorm3 Ministry of Health costing survey

Time spent on training and reporting: median days	1.5	0.625	—	Ministry of Health costing tool
Time spent on drug delivery: median days	2	0.25	—	DeWorm3 school survey
Allowances given	Per training	Per training	—	Ministry of Health costing tool, DeWorm3 costing tool
Allowance rate	$17.01	$1.42	—	DeWorm3 costing tool
**DeWorm3 Enumerators**				
Number staff involved: mean (range)	90 (90)	84 (73—107)	57 (50—65)	DeWorm3 costing tool
Daily compensation and allowances	$8.55	$6.50	$14—$34[e]	DeWorm3 costing tool
**DeWorm3 Field Supervisors (Controllers, Field Supervisors, Field Officers)**				
Number staff involved	10	13	4	DeWorm3 costing tool
Daily compensation and allowances: approximate	$21	$12	$20	DeWorm3 costing tool
**DeWorm3 Vehicle Costs**				
Project vehicles	2	1	5	DeWorm3 costing tool
Make of vehicles	Nissan 4x, 5-seater	Mahindra Thar CRDe	Land cruiser 4.2 Diesel 13-seater	DeWorm3 costing tool
Net cost	$37,807	$13,755	$41,137	DeWorm3 costing tool
Useful life years assumed	9	9	9	WHO CHOICE
**DeWorm3 Central Personnel**				
DeWorm3 central key program staff (providing program management and higher level supervision) involved	11	11	8	DeWorm3 costing tool
DeWorm3 central support staff (drivers, accountants, etc.) involved	10	7	10	DeWorm3 costing tool

Acronyms: community-wide mass drug administration (cMDA), school-based deworming (SBD), Gross Domestic Product (GDP), National Deworming Day (NDD), GlaxoSmithKline (GSK)
Note: Dashes (–) represent situations where no data was observed (e.g. no allowances given, no staff involved).
[a] GlaxoSmithKline (GSK) is currently donating albendazole for lymphatic filariasis and soil-transmitted helminth control. The estimated opportunity costs of donated albendazole is $0.45 per tablet. We have also included the cost of shipping, raising the total estimated costs to $0.047. Costs per tablet administered also include 10% wastage, bringing the total to 0.052. Although GSK-donated albendazole was used in the DeWorm3 project, this analysis used the estimated costs of locally procured albendazole in India, as is routinely used in National Deworming Days. Estimated cost per tablet of locally procured albendazole was acquired from the Tamil Nadu State Budget for National Deworming Day.
[b] Type of allowance varied per country (i.e., lunch allowance, mobile data, travel allowance, etc.). Given the travel nature of the work, and the descriptions of these costs, we have chosen to present these costs as allowances rather than compensation for work done. In some countries, the allowances vary based on number of days involved or number of persons reached.
[c] Information on schools, teachers, and other school staff is specific to control clusters only within the DeWorm3 study. Although SBD was implemented within all clusters in the DeWorm3 study (n=40) per each country's national deworming strategy, SBD was only costed within control clusters (n=20).
[d] Salary varies based on level of school.
[e] Some nurses functioned as enumerators were paid a higher rate.

For peer review only - http://bmjopen.bmj.com/site/about/guidelines.xhtml

Appendix 2: Table 4: Key assumptions regarding unit cost analysis for community-wide mass drug administration and school-based deworming

Type of cost	Description of costs	Assumptions	Analysis decisions
Trial/research costs	Costs related to conducting the trial component of DeWorm3, such as trial insurance, developing IRB materials, etc.	Trial-related costs exclusively related to research did not affect MDA coverage.	Trial-related costs were excluded from cMDA and SBD unit cost analyses.
Planning costs	Activities related to starting up the trial such as micro-planning, recruitment, procurement, trial sensitization meetings, and development of IEC and training materials.	Planning was relevant to all field activities (census, prevalence survey, cMDA, SBD, and coverage survey).	Planning costs were annualized over 3 years of program implementation and split across activities based on the number of days activities were implemented. When monthly or annual costs needed to be split by days, we assumed 20.5 workdays per month.
Program management costs	Program management costs were fixed costs and included large capital items, rent, and salaried project staff. Program management resources were used in multiple trial activities, (generally) purchased/ employed/ rented/ donated in the planning stages of the trial, and were retained for the duration of the trial.	Program management was relevant to all field activities (Census, prevalence survey, cMDA, SBD, and coverage survey).<br><br>There may have been inefficiencies in resource use. For example, a vehicle that was purchased by DeWorm3 may not be driven every day.<br><br>Some materials that were already owned by the DeWorm3 team would need to be purchased by future implementing organizations.	Capital items were annualized over their useful life years, with a 3% discount rate.<br><br>Costs were split among annual activities based on the number of days spent on each activity. When monthly or annual costs needed to be split by days, we assumed 20.5 workdays per month.<br><br>When costs were shared among multiple programs within the implementing institution, we allocated a percentage of costs towards DeWorm3 (i.e. only a portion of total rent costs for an implementing organization were allocated to DeWorm3, if the organization had multiple grants/projects). When resources were used only by the DeWorm3 project, we assumed full costs of resources, even if not used at full capacity.<br><br>Resources that were already owned by the DeWorm3 team (i.e., vehicles, computers, etc.) were categorized as financial costs in this analysis.
Census costs	All costs to run an annual census conducted prior to MDA in all 40 clusters.	Censuses did not affect MDA coverage.	Census costs were excluded from the cMDA and SBD unit cost analysis.
Prevalence survey costs	An annual prevalence survey was used to assess STH prevalence across the 40 clusters.	In year 1, a longitudinal monitoring cohort (LMC) of approximately 6,000 persons was conducted, in addition to a cross-sectional survey of 20,000 persons, per country. In year 2, only the longitudinal monitoring cohort was conducted in Benin and India (no prevalence surveys were conducted in Malawi year 2). It is therefore assumed that approximately 1/4 of shared prevalence survey costs were relevant to the LMC, and 3/4 to the cross-sectional survey, in year 1.<br><br>Prevalence surveys did not affect MDA coverage.	We have presented only the costs of the LMC in this manuscript. Approximately ¼ of shared prevalence survey costs in year 1 were allocated to the LMC.<br><br>Prevalence survey costs were excluded from the cMDA and SBD unit cost analysis and were presented separately.
Coverage surveys	All costs related to conducting post-MDA coverage surveys: conducted after each round of cMDA, sampling approximately 8,000 individuals from the 40 clusters.	Coverage surveys did not affect cMDA coverage.	Coverage survey costs were excluded from the cMDA and SBD unit cost analysis and were presented separately.
DeWorm3 vehicle costs	DeWorm3 project vehicles and related costs (fuel, maintenance, etc), as well as hired vehicles.	Project and hired vehicles were used for additional supervision by DeWorm3 field staff and enumerator transport.	DeWorm3 project vehicles and hired vehicles used in cMDA and SBD were designated as “supporting” costs unless specified as a routine cost (i.e., vehicle hired for government supervisor, fuel reimbursement for training participant, etc.).

Shared MDA (cMDA/SBD) costs	Resources or costs that were described as shared between cMDA and SBD.	In rounds where cMDA was implemented directly after SBD, many sensitization activities were relevant to both cMDA and SBD.<br><br>In rounds where cMDA was implemented directly after SBD, most training activities were relevant to both cMDA and SBD.	Shared costs were split between cMDA and SBD proportionally based on the number of days of each activity (for example, for training costs), or by population treated (for example, for side effects medication).
Input classification for per-diems and allowances	Costs that were described as per-diems or allowances to implementers, trainers, supervisors, or community members.	Unless specified that costs were incentives or compensation, allowances and per-diems were assumed to be used for their designated purpose (for example, lunch allowances used to purchase lunch, travel allowances used for transport).	Per-diems and allowances that were specified as transport allowances, were assigned "vehicles and overheads" as the input classification.<br><br>Per-diems and allowances that were not specified as transport allowances, were assigned "wages and per-diems" as the input classification.<br><br>Unless specified that costs were incentives or compensation, allowances and per-diems were not considered compensation and were not subtracted from estimated opportunity costs. For example, if CDDs were provided a lunch or travel allowance during fieldwork, this was not considered compensation for work done.

Acronyms: community-wide mass drug administration (cMDA), mass drug administration (MDA), school-based deworming (SBD), community drug distributor (CDD), longitudinal monitoring cohort (LMC).

For peer review only

Appendix 3: Costs in local currency

In the following tables, we present key costing data from the manuscript, presented in local currency. Costs are presented in 2019 West African Francs (XOF) for Benin, 2019 Indian Rupees (INR) for India, and 2019 Malawian Kwacha (MWK) for Malawi.

Appendix 3: Table 1: Total economic costs and number of treatments administered through community-wide mass drug administration and school-based deworming, per country-round, in local currency

Metric	Benin (XOF)		India (INR)		Malawi (MWK)	
	cMDA	SBD	cMDA	SBD	cMDA	SBD
Number of treatments administered[a]						
Round 1	45,280	–	55,953	15,266	49,518	–
Round 2	37,913	9,298	55,758	19,152	38,641	16,077
Round 3	42,398	–	57,353	21,396	52,122	–
Round 4	32,529	10,343	57,398	20,586	49,709	12,964
Total costs[b]						
Round 1	61,148,760	–	5,068,089	975,635	94,544,024	–
Round 2	47,069,592	12,912,370	4,536,205	992,149	71,212,140	16,716,181
Round 3	57,116,293	–	4,656,850	900,980	70,936,162	–
Round 4	54,342,916	14,882,855	4,352,396	898,775	72,724,580	17,999,058
Cost per treatment administered						
Round 1	1,350	–	91	64	1,909	–
Round 2	1,242	1,389	81	52	1,843	1,040
Round 3	1,347	–	81	42	1,361	–
Round 4	1,671	1,439	76	44	1,463	1,388

Acronyms: community-wide mass drug administration (cMDA), school-based deworming (SBD)
Note: Dashes (–) represent situations where no data was collected. SBD was only implemented annually in Benin and Malawi, so no data were available for rounds 1 and 3.
[a] Treatments administered for cMDA includes all eligible individuals who received treatment by DeWorm3 through cMDA in the intervention clusters (Source: DeWorm3 MDA treatment logs). Population treated for SBD includes all children treated in schools within the DeWorm3 control clusters (Source: SBD treatment logs).
[b] Total costs include both financial and opportunity costs.

For peer review only

Appendix 3: Table 2: Average unit costs (2019 local currency) for community-wide mass drug administration across two years
Total economic costs are presented, as well as a breakdown of costs by routine vs. supporting activities, and financial vs. opportunity costs

	Benin (XOF)[a]	India (INR)[a]	Malawi (MWK)[a]
**Planning**	**61**	**3**	**5**
Supporting (financial)	61	3	5
**Program management**	**371**	**28**	**376**
Routine (financial)	164	11	113
Routine (opportunity) – time costs for government staff[b]	6	–	0
Supporting (financial)	200	17	263
**Community sensitization**	**143**	**12**	**125**
Routine (financial)	64	1	46
Routine (opportunity) – time costs for government staff and volunteers	8	0	27
Supporting (financial) – additional sensitization activities	4	0	10
Supporting (financial) – NGO supervision	66	10	42
**Training costs**	**196**	**8**	**190**
Routine (financial)	70	0	49
Routine (opportunity) – time costs for government staff and volunteers	14	2	18
Supporting (financial) – training of electronic data collectors	63	4	41
Supporting (financial) – NGO supervision and training support	50	1	82
**Drug delivery**	**631**	**32**	**948**
Routine (financial)	210	5	152
Routine (opportunity) – time costs for government staff and volunteers	90	8	136
Routine (opportunity) – donated drugs	0	1	0
Supporting (financial) – electronic data capture	172	13	228
Supporting (financial) – NGO supervision	159	5	433
Average unit costs[c]	***1402***	***82***	***1644***
Routine (financial)	*509*	*18*	*360*
Routine (opportunity)	*118*	*11*	*181*
Supporting (financial)	*776*	*53*	*1103*

Acronyms: non-governmental organization (NGO)
Note: Dashes (–) represent situations where no costs were observed.
[a] Analysis includes two years of cMDA. As cMDA was conducted bi-annually in each country, results are presented as the average across four rounds.
[b] Government staff include supervisory and implementing staff whose salaries are paid by the Ministry of Health. Examples include nurses and health officers, HSAs (Malawi only), as well as national and subnational government officials involved in the program.
[c] Routine and supporting activities and related resources are described in Appendix 2: Table 2. Financial costs represent actual expenditure on goods and services purchased by the government or NGO implementing partner. Opportunity costs, on the other hand, include costs forgone by using a resource in a particular way. These opportunity costs recognize and value the cost of using resources, as these resources are then unavailable for productive use elsewhere. Opportunity costs in this analysis include costs of donated albendazole, volunteer time spent on the project (such as volunteer drug distributors), and estimated government staff salary costs.

For peer review only

Appendix 3: Table 3: Average unit costs (2019 local currency) for school-based deworming across two years
Total economic costs are presented, as well as a breakdown of costs by routine program vs. supporting program activities, and financial vs. opportunity costs

	Benin (XOF)[a]	India (INR)[b]	Malawi (MWK)[a]
**Planning**	**43**	**0**	**4**
Supporting (financial)	43	0	4
**Program management**	**406**	**14**	**299**
Routine (financial)	–	–	110
Routine (opportunity) – time costs for government staff[c]	146	8	1
Supporting (financial)	260	6	187
**Community sensitization**	**153**	**0**	**89**
Routine (financial)	83	0	29
Routine (opportunity) – time costs for government staff and volunteers	–	–	39
Supporting (financial) – additional sensitization activities	27	–	5
Supporting (financial) – NGO supervision	44	–	15
**Training costs**	**357**	**13**	**189**
Routine (financial)	157	1	57
Routine (opportunity) – time costs for government staff and volunteers	119	10	83
Supporting (financial) – training of electronic data collectors	34	1	18
Supporting (financial) – NGO supervision and training support	47	1	31
**Drug delivery**	**454**	**23**	**634**
Routine (financial)	72	1	165
Routine (opportunity) – time costs for government staff and volunteers	329	20	130
Routine (opportunity) – donated drugs	0	1	0
Supporting (financial) – electronic data capture	9	1	154
Supporting (financial) – NGO supervision	44	0	186
Average unit costs[d]	***1414***	***50***	***1214***
Routine (financial)	*311*	*2*	*360*
Routine (opportunity)	*594*	*38*	*253*
Supporting (financial)	*508*	*10*	*601*

Acronyms: non-governmental organization (NGO)
Note: Dashes (–) represent situations where no costs were observed.
[a] Analysis includes two years of SBD. In India, SBD was conducted bi-annually, so results are presented as the average across four rounds.
[b] Analysis includes two years of SBD. In Malawi and Benin, SBD was conducted annually, so results are presented as the average of two rounds.
[c] Government staff include supervisory and implementing staff whose salaries are paid by the Ministry of Health. Examples include nurses and health officers, teachers, and national and subnational government officials involved in the program.
[d] Routine and supporting activities and related resources are described in Appendix 2: Table 2. Financial costs represent actual expenditure on goods and services purchased by the government or NGO implementing partner. Opportunity costs, on the other hand, include costs forgone by using a resource in a particular way. These opportunity costs recognize and value the cost of using resources, as these resources are then unavailable for productive use elsewhere. Opportunity costs in this analysis include costs of donated albendazole, volunteer time spent on the project (such as volunteer drug distributors), and estimated government staff salary costs.

For peer review only

Appendix 3: Table 4: Annual costs of additional deworming program activities, including censuses, prevalence surveys, and coverage surveys, across two years of implementation (2019 local currency)

Activity	Metric	Benin (XOF)		India (INR)		Malawi (MWK)	
		Year 1	Year2	Year 1	Year2	Year 1	Year2
Census[a]	Population censused	94,969	88,647	140,932	146,321	121,819	119,418
	Total cost	100,718,503	58,290,985	6,521,106	5,603,244	151,397,733	101,756,373
	Cost per person censused	1,061	658	46	38	1,243	852
Prevalence survey[a]	Population surveyed	6,814	5,283	6,503	6,158	6,935	–
	Total cost	74,426,119	67,624,070	5,487,075	12,480,670	93,810,394	–
	Cost per person surveyed	10,923	12,800	844	2,027	13,527	–
Coverage survey[a]	Population surveyed	16,339	16,130	15,573	14,809	16,796	17,166
	Total cost	44,466,307	38,946,531	1,459,531	1,392,526	51,862,081	41,869,871
	Cost per person surveyed	2,721	2,415	94	94	3,088	2,439

[a]For activities that spanned all 40 clusters, about 50% of the individuals surveyed were from intervention clusters, and the other 50% from control clusters.
Note: Dashes (–) represent situations where no data was collected. A prevalence survey was not conducted in Malawi in year 2.

For peer review only

Appendix 4: Additional details of sensitivity analysis methodology

One-way sensitivity analyses: In one-way sensitivity analyses, opportunity costs of drugs, opportunity costs of volunteer time, and coverage rates were explored.

Opportunity costs for albendazole in the costing analysis were valued using the estimated valuation of donated albendazole from GlaxoSmithKline (GSK) plus estimated shipping costs in Malawi and Benin, and the market price of locally procured albendazole in Tamil Nadu, India.[4] To date, GSK has committed to donating albendazole to combat STH until 2025.[5] After 2025, the cost of albendazole to STH programs is unknown. In sensitivity analyses, costs of albendazole were explored by removing opportunity costs as the low input (to explore financial costs to governments during albendazole donation programs) and doubling the opportunity costs of albendazole as the high input (doubling the global valuation of donated albendazole and doubling the India market price to explore how increases in albendazole costs could affect unit costs).

Opportunity costs for volunteers' time in the costing analysis were valued using national (Benin, Malawi) and subnational (India) average wage rates acquired from labor surveys.[6-7] In sensitivity analyses, volunteer time costs were altered by removing opportunity costs for the low input (with the assumption that lunch and travel allowances were sufficient forms of compensation). For the high input, opportunity costs for community volunteers who played a health-delivery role were valued using the estimated salaries of an equivalent health worker.[8]

Total treatments administered per country-round were used in the costing analysis. In sensitivity analyses, coverage rates (and therefore total treatments administered) were altered by applying the highest and lowest observed cluster coverage in a given country to the eligible population for treatment, demonstrating the observed ranges in coverage possible in a given location.

Two-way sensitivity analyses: Two-way sensitivity analyses were also conducted to determine the influence of reductions in supporting activities or sensitization activities alongside reductions in coverage.

The DeWorm3 Project prioritized high coverage of cMDA, intending to reach 90% coverage in each cluster.[9] To do so, the project employed additional supervision and electronic data collection to track coverage in real-time (e.g. "supportive activities"), and respond with mop-up in low coverage areas. These additional activities were resource-intensive, and may not be included in future routine programs. However, removing these additional activities may affect program coverage. In sensitivity analyses, we have explored a two-way analysis where cMDA routine costs are removed, and cMDA coverage rates were reduced by 30% to align more closely with historic deworming coverage rates.[3] Additionally, although SBD is routinely implemented by the governments of India, Benin, and Malawi, the interventions were altered to different extents for delivery during DeWorm3. For example, in Malawi, SBD was implemented through the DeWorm3 project team, rather than via the government of Malawi, leading to different program management costs. In Benin, the government implemented SBD, though the DeWorm3 team provided additional support in the form of supervision and additional sensitization. In sensitivity analyses, SBD coverage was reduced 10% alongside the removal of supportive activities, to reflect how these supportive activities might be increasing coverage during the trial. The relationship between supporting activity costs and coverage rates has not been validated, and future analyses may explore additional changes to input values.

To reach a goal of 90% coverage in each cluster, the DeWorm3 project implemented multiple community sensitization efforts that may have gone above and beyond activities implemented by the government. In two-way sensitivity analyses, the relationship between sensitization costs and coverage rates was explored. For the high-input: sensitization costs were increased 30% with an increase of 10% in coverage rates (not exceeding 100% coverage of eligible populations). For the low-input, sensitization costs were decreased 30% with a decrease of 10% in coverage rates. The relationship between sensitization costs and coverage rates has not been validated, and future analyses may explore additional changes to input values.

Future directions for sensitivity analyses: Given the many costing resources that were included in this analysis, there are endless possibilities of costs that could be altered in sensitivity analyses. Decisions regarding which sensitivity analyses to conduct in this study were based upon field team and expert input regarding influential factors, and differences in implementation across DeWorm3 sites. Future discussions with government stakeholders may provide opportunities to explore how costs may vary in scaled-up programs (e.g. specific allowances for CDDs, frequency and resources needed for training, days of MDA, etc.) allowing for tailored sensitivity analyses.

For peer review only

For peer review only - http://bmjopen.bmj.com/site/about/guidelines.xhtml

Appendix 5: Additional costing results

In the following tables and figures, we present supplemental costing data not presented in the manuscript, including a further breakdown of costs by supporting vs routine activities, fixed vs variable inputs, and costs across rounds.

Appendix 5: Table 1: Percentage of DeWorm3 activity unit costs that are fixed vs. variable, by activity and country

	Benin		India		Malawi	
activity	Fixed unit cost	Variable unit cost	Fixed unit cost	Variable unit cost	Fixed unit cost	Variable unit cost
**Census1**	26%	74%	27%	73%	15%	85%
**Census2**	59%	41%	28%	72%	26%	74%
**Coverage survey1**	25%	75%	27%	73%	13%	87%
**Coverage survey2**	27%	73%	32%	68%	15%	85%
**Coverage survey3**	16%	84%	28%	72%	17%	83%
**Coverage survey4**	14%	86%	28%	72%	17%	83%
**Prevalence survey1**	34%	66%	40%	60%	14%	86%
**Prevalence survey2**	63%	37%	42%	58%	–	–
**cMDA1**	23%	77%	23%	77%	16%	84%
**cMDA2**	25%	75%	26%	74%	17%	83%
**cMDA3**	33%	67%	23%	77%	19%	81%
**cMDA4**	26%	74%	24%	76%	18%	82%
**SBD1**	–	–	7%	93%	–	–
**SBD2**	16%	84%	9%	91%	19%	81%
**SBD3**	–	–	8%	92%	–	–
**SBD4**	21%	79%	8%	92%	19%	81%

Acronyms: community-wide mass drug administration (cMDA), school-based deworming (SBD)
Note: Dashes (–) represent situations where no data was collected. SBD was only implemented annually in Benin and Malawi, so no data were available for rounds 1 and 3.

For peer review only

Appendix 5: Figure 1: Total financial and opportunity costs of a) community-wide mass drug administration and b) school-based deworming by input-classification (including routine and supporting program costs).

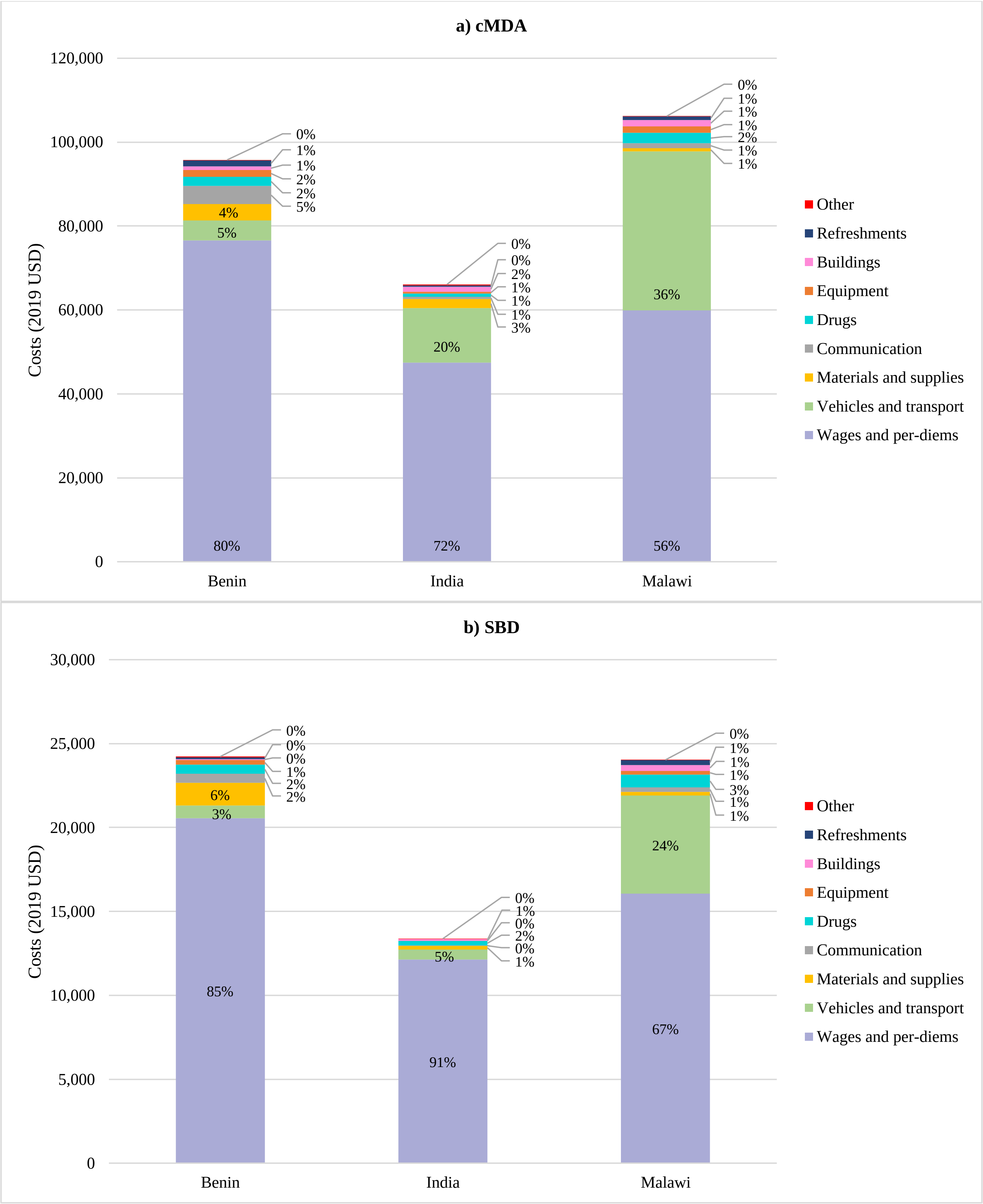

Note: For simplicity, labels were rounded to the nearest whole number. In situations where 0% is listed, category costs contributed less than 0.5% of total costs.

Appendix 5: Table 2: Community-wide mass drug administration costs by input classification, by routine vs. supporting costs

Category	Benin		India		Malawi	
	Routine	Supporting	Routine	Supporting	Routine	Supporting
Buildings and overheads	1%	1%	2%	2%	1%	1%
Communication	5%	5%	1%	1%	1%	1%
Drugs	0%	–	3%	–	0%	–
Equipment and overheads	2%	2%	1%	1%	3%	1%
Materials and supplies	7%	2%	6%	2%	1%	1%
Other	0%	0%	0%	0%	0%	0%
Refreshments	2%	1%	1%	0%	2%	0%
Vehicles and overheads	5%	5%	4%	28%	13%	48%
Wages and per-diems	78%	85%	81%	66%	78%	48%

Note: Dashes (–) represent situations where no costs were observed.

Appendix 5: Table 3: School-based deworming costs by input classification, by routine vs. supporting costs

Category	Benin		India		Malawi	
	Routine	Supporting	Routine	Supporting	Routine	Supporting
Buildings and overheads	0%	1%	0%	3%	1%	2%
Communication	2%	3%	–	1%	1%	1%
Drugs	0%	–	2%	–	0%	–
Equipment and overheads	0%	3%	–	1%	1%	1%
Materials and supplies	7%	3%	1%	2%	1%	1%
Other	0%	0%	0%	0%	0%	0%
Refreshments	0%	0%	–	–	2%	0%
Vehicles and overheads	2%	5%	0%	21%	11%	40%
Wages and per-diems	88%	85%	96%	71%	83%	55%

Note: Dashes (–) represent situations where no costs were observed.

For peer review only - http://bmjopen.bmj.com/site/about/guidelines.xhtml

Appendix 5: Figure 2: Mean and range of unit costs per sub-activity across four rounds of community-wide mass drug administration in Benin, India, and Malawi; two rounds of school-based deworming in Benin and Malawi; and, four rounds of school-based deworming in India.

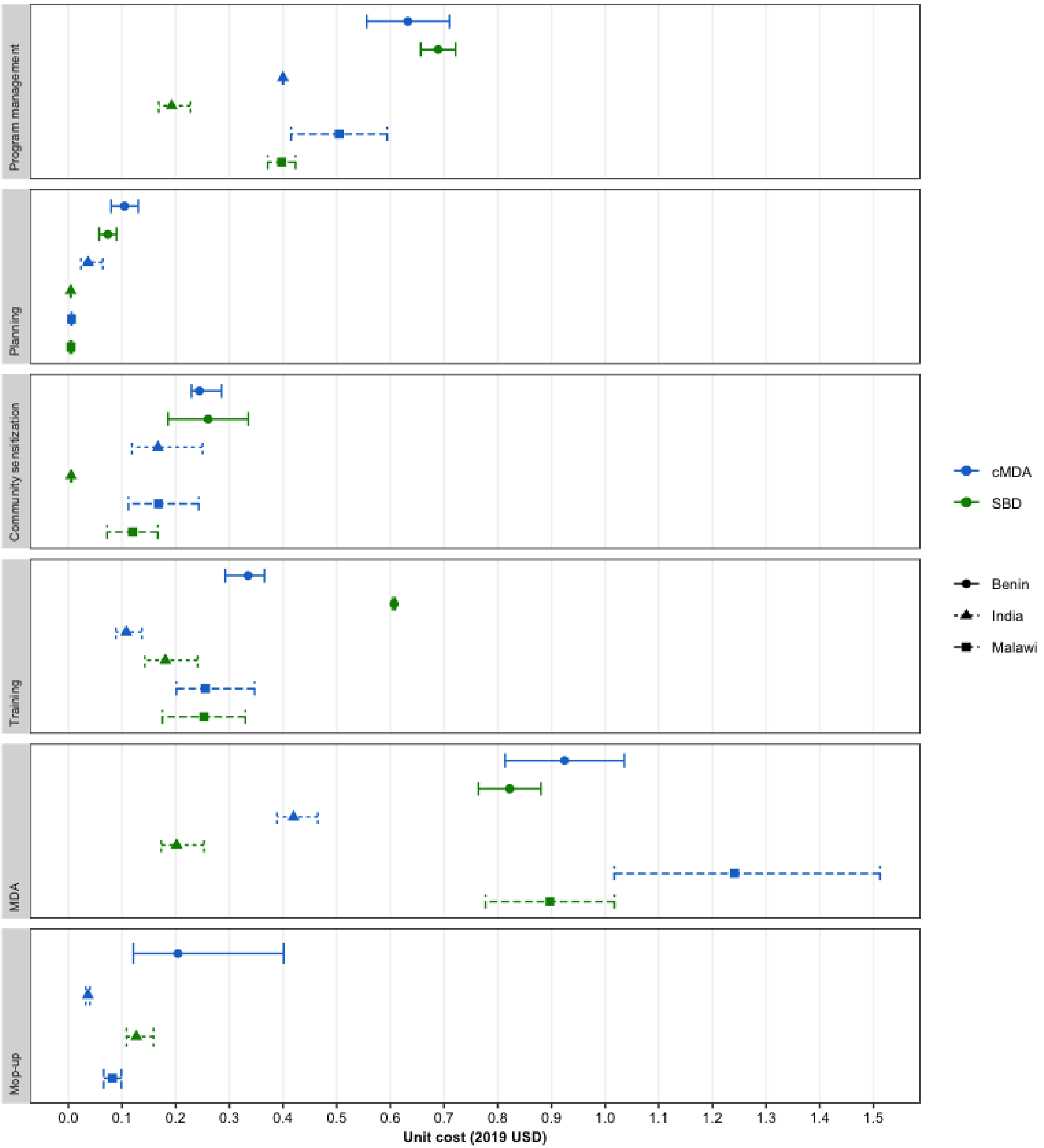

Acronyms: community-wide mass drug administration (cMDA), school-based deworming (SBD)

Appendix 5: Table 4: Annual costs of additional deworming program activities, including censuses, prevalence surveys and coverage surveys, across two years of implementation (2019 USD ($))

	Benin: Year 1	Benin: Year 2	India: Year 1	India: Year 2	Malawi: Year 1	Malawi: Year 2
**Annual census**[a]						
Population censused	94,969	88,647	140,932	146,321	121,819	119,418
Total cost	$ 171,889	$ 99,481	$ 92,603	$ 79,569	$ 203,071	$ 136,487
Cost per person censused	$ 1.81	$ 1.12	$ 0.66	$ 0.54	$ 1.67	$ 1.14
**Annual prevalence survey** following a cohort of approximately 5,000-7,000 individuals[a]						
Population surveyed	6,814	5,283	6,503	6,158	6,935	—
Total cost	$ 127,018	$ 115,409	$ 77,919	$ 177,231	$ 125,829	—
Cost per person surveyed	$ 18.64	$ 21.85	$ 11.98	$ 28.78	$ 18.14	—
**Bi-annual coverage survey** conducted after each round of cMDA, sampling approximately 8,000 individuals[a]						
Population surveyed	16,339	16,130	15,573	14,809	16,796	17,166
Total cost	$ 75,887	$ 66,467	$ 20,726	$ 19,774	$ 69,563	$ 56,160
Cost per person surveyed	$ 4.64	$ 4.12	$ 1.33	$ 1.34	$ 4.14	$ 3.27

[a] Activity spanned all 40 clusters, with about 50% of the individuals surveyed were from intervention clusters, and the other 50% from control clusters.
Note: Dashes (–) represent situations where no data was collected. A prevalence survey was not conducted in Malawi in year 2.

For peer review only - http://bmjopen.bmj.com/site/about/guidelines.xhtml

Appendix 6: Description of cost differences across countries

In the following tables, we provide further details and reasoning regarding differences in observed cMDA and SBD costs across countries.

Appendix 6: Table 1: Drivers of heterogeneity in costs across sites

Type of difference	Description	Differences in costs across countries
Number of persons targeted and treated	The number of persons censused in each study site varied, with the smallest population in Benin (approx. 90,000), followed by a larger population in Malawi (approx. 120,000), and the largest censused population in India (approx. 145,000). These differences in overall population sizes affected the total number of persons targeted and treated via community-wide mass drug administration (cMDA).<br><br>Additionally, the age composition in each site varied, leading to variability in the number of targeted children for school-based delivery (SBD). The percent of the population that was pre-school or school-aged was lowest in India, followed by Benin, and highest in Malawi. The number of children who were treated through SBD was lowest in Benin, then increased in India, and was highest in Malawi.	The number of persons treated may have affected overall costs per round of treatment, as more resources may have been required to reach a larger targeted population.<br><br>The number of persons treated had a large effect on the unit costs (cost per treatment administered). For example, the total costs per round of SBD were similar in Malawi and Benin (see Table 1, main text), however, the unit costs were much lower in Malawi given the larger number of children treated via SBD (approximately 50% more children were treated in Malawi than in Benin).
Costs per input-classification	Vehicle costs in Malawi were substantially higher than in Benin and India. Reasons for higher costs include more project vehicles (5 total vehicles were used in Malawi, compared to 2 vehicles in Benin and 1 in India). Additionally, the Malawi DeWorm3 team chose to organize travel for enumerators and HSAs centrally, requiring more car hires and fuel. When cars and drivers were hired to support activities, they were hired for more days in Malawi than in other countries, as MDA was generally 3-4 days longer in Malawi (see Appendix 2: Table 3). In India and Benin, enumerators and CDDs were provided travel allowances, which resulted in lower overall vehicle costs.	When examining cMDA and SBD costs by input-classification (Appendix 5: Figure 1), Malawi had a substantially higher percentage of costs that were allocated to vehicles and overheads, compared to India and Benin. Total costs per round of cMDA were generally highest in Malawi, in partial, due to vehicle costs. The highest cost of cMDA was observed in Malawi round 1, driven by a larger number of vehicles rented.
Planning and program management costs	Resources for planning and program management varied across sites.<br><br>More time was spent on planning in Benin, leading to higher planning costs. Additionally, full-time equivalent costs for central DeWorm3 staff were higher in Benin, leading to higher program management costs.<br><br>Involvement of the DeWorm3 team in SBD varied across countries, and therefore the share of DeWorm3 program management costs allocated to SBD varied across countries. In India, SBD was implemented by the government through the bi-annual National Deworming Day (NDD). Therefore, the DeWorm3 team was only minorly involved in SBD delivery, mainly to observe and record data. In Malawi, the DeWorm3 team was solely responsible for implementing SBD in DeWorm3 clusters, with light supervision from the government. In Benin, the implementation of SBD was led by the government, however, the DeWorm3 team was heavily involved in the coordination and supervision.	cMDA planning and supervision costs were highest in Benin, followed by Malawi, and lowest in India, generally driven by wages (Benin) and vehicles (Malawi).<br><br>Program management costs for SBD were much lower in India, given the DeWorm3 team provided less implementation support and supervision compared to the other countries.
Opportunity costs for albendazole	Albendazole used in the project was donated, however, common practice in costing analyses is to estimate the opportunity costs of drugs (i.e., the costs of the drugs if they were used for other purposes, rather than donated). Albendazole is locally produced in India, so we estimated opportunity costs in India using the local per-tablet price. In Benin and Malawi, albendazole is procured from global suppliers. We estimated opportunity costs in Benin and Malawi as the GlaxoSmithKline valuation of donated albendazole, as $0.045 per-tablet, plus the estimated costs of shipping at $0.0019, for a total value of $0.052.[1] We also estimated approximately 10% buffer stock.	Opportunity costs of albendazole are lower in India than in Benin and Malawi, resulting in about a $0.05 difference in unit costs.

Opportunity costs for government-funded staff and volunteers	The number, type, salaries, and time involved for currently employed government staff and volunteers varied across settings.<br><br>CDDs and volunteers: In Malawi, the primary drug distributors (HSAs), were government-funded staff whereas the primary drug distributors in Benin and India (CDDs) were volunteers. Fewer HSAs were involved in Malawi compared to CDDs in Benin and India, however, salaries were higher in Malawi.<br><br>Teachers and school staff: Malawi had the fewest number of teachers and school staff involved in drug delivery, with more staff involved in Benin, and the greatest number in India. School directors were also involved in SBD in Benin. However, the time spent by teachers on delivery varied, with the smallest amount of time in India, and the greatest amount in Malawi. Monthly salaries for teachers in Benin and India were similar; teacher salaries were approximately 50% lower in Malawi. See Appendix 2: Table 3 for more details.<br><br>Government supervisors: Fewer government staff were involved in the supervision of SBD and cMDA in Malawi, as the DeWorm3 team was the primary implementer.	Opportunity costs were similar across countries for cMDA.<br><br>A large number of school staff in Benin and India (including additional involvement of Anganwadi Workers in India and school directors in Benin) and higher teachers' salaries led to higher opportunity costs for SBD. School staff opportunity costs were lowest in Malawi.
Financial resources for community sensitization	Community sensitization activities varied across sites.<br><br>Benin activities included community-level meetings, public criers, radio broadcasts, and printed materials. Benin also included teacher sensitization for SBD in year 2.<br><br>India activities focused on distributing printed materials (banners and flyers), and community-level meetings (cMDA only).<br><br>Malawi activities included community-level meetings, public announcements, drama groups, and a football bonanza (round 4).<br><br>See Appendix 1: Table 2, for more details.	Sensitization costs were highest in Benin, due to more activities implemented.<br><br>Costs for SBD sensitization were substantially lower in India, as costs were only related to printed materials.
Financial resources for training	In India, SBD is routinely conducted bi-annually and resources for implementation are kept quite low. The only routine financial costs reported by the government were transport allowances provided to teachers. The DeWorm3 team's involvement in SBD training was minimal.<br><br>In Benin and Malawi, the DeWorm3 team was involved in training, and therefore more financial costs were incurred such as printed materials, refreshments, and equipment and hall hires for training sessions.<br><br>Similarly, Benin and Malawi used more financial resources such as equipment, mobile minutes, and refreshments for cMDA training, compared to India.	Financial costs for SBD and cMDA training were substantially lower in India, likely because it was completely government-led.
Financial resources for drug delivery: SBD	In India, SBD is routinely conducted bi-annually and resources for implementation are kept quite low. In the DeWorm3 project, SBD continued to be implemented through the government routinely. Few financial resources are required during drug delivery, only allowances for some key staff (VHNs, ASHAs, and for supervision).<br><br>In Benin and Malawi, the DeWorm3 team was more involved in drug delivery. Therefore, more resources were used such as fuel, allowances (such as travel, communication, lunch) for CDDs/HSAs, refreshments, and allowances for DeWorm3 coordinating and supervisory staff.	Financial costs for SBD were substantially lower in India, likely because it was completely government-led with involvement from DeWorm3 limited to data collection.
Financial resources for drug delivery: cMDA	In Benin, the DeWorm3 team collaborated closely with the Ministry of Health to implement cMDA. Therefore, many allowances and travel costs were incurred for supervision and coordination efforts by both the Ministry of Health and the DeWorm3 team. Additionally, cMDA mop-up required more resources in Benin, mainly due to a large mop-up campaign in cMDA round 4. In Benin, cMDA round 4 was interrupted by a natural disaster (flooding). To reach higher coverage rates, a more involved mop-up campaign was implemented one month after MDA, with additional sensitization and training.<br><br>In India and Malawi, the DeWorm3 was primarily responsible for cMDA drug delivery, with few allowances paid to government supervisors.	Routine financial costs were higher in Benin, due to more supervision costs and the more involved mop-up campaign in round 4 of MDA.

Appendix 7: Appendix references

1 Turner HC, Bettis AA, Chu BK, et al. Investment Success in Public Health: An Analysis of the Cost-Effectiveness and Cost-Benefit of the Global Programme to Eliminate Lymphatic Filariasis. *Clin Infect Dis* 2017; **64**: 728–35.

2 International Labor Organization. ILOSTAT database. https://ilostat.ilo.org/data (accessed Aug 25, 2021).

3 Tamil Nadu State Government Department of Evaluation and Applied Research. Tamil Nadu - An Economic Appraisal 2011-12 to 2013-14.

4 Turner HC, Bettis AA, Chu BK, et al. Investment Success in Public Health: An Analysis of the Cost-Effectiveness and Cost-Benefit of the Global Programme to Eliminate Lymphatic Filariasis. *Clin Infect Dis* 2017; **64**: 728–35.

5 GlaxoSmithKline. Neglected Tropical Diseases: Our commitment. 2021; published online Jan. https://www.gsk.com/media/6543/ntd-factsheet-jan2021.pdf.

6 International Labor Organization. ILOSTAT database. https://ilostat.ilo.org/data (accessed Aug 25, 2021).

7 Tamil Nadu State Government Department of Evaluation and Applied Research. Tamil Nadu - An Economic Appraisal 2011-12 to 2013-14.

8 Serje J, Bertram MY, Brindley C, et al. Global health worker salary estimates: an econometric analysis of global earnings data. *Cost Eff Resour Alloc* 2018; **16**: 10.

9 Werkman M, Toor J, Vegvari C, et al. Defining stopping criteria for ending randomized clinical trials that investigate the interruption of transmission of soil-transmitted helminths employing mass drug administration. *PLoS Negl Trop Dis* 2018; **12**: e0006864.

For peer review only

CHEERS Checklist
Items to include when reporting economic evaluations of health interventions

The **ISPOR CHEERS Task Force Report**, *Consolidated Health Economic Evaluation Reporting Standards (CHEERS)—Explanation and Elaboration: A Report of the ISPOR Health Economic Evaluations Publication Guidelines Good Reporting Practices Task Force*, provides examples and further discussion of the 24-item CHEERS Checklist and the CHEERS Statement. It may be accessed via the *Value in Health* or via the ISPOR Health Economic Evaluation Publication Guidelines – CHEERS: Good Reporting Practices webpage: http://www.ispor.org/TaskForces/EconomicPubGuidelines.asp

Section/item	Item No	Recommendation	Reported on page No/ line No
**Title and abstract**			
Title	1	Identify the study as an economic evaluation or use more specific terms such as "cost-effectiveness analysis", and describe the interventions compared.	
Abstract	2	Provide a structured summary of objectives, perspective, setting, methods (including study design and inputs), results (including base case and uncertainty analyses), and conclusions.	
**Introduction**			
Background and objectives	3	Provide an explicit statement of the broader context for the study.<br>Present the study question and its relevance for health policy or practice decisions.	
**Methods**			
Target population and subgroups	4	Describe characteristics of the base case population and subgroups analysed, including why they were chosen.	
Setting and location	5	State relevant aspects of the system(s) in which the decision(s) need(s) to be made.	
Study perspective	6	Describe the perspective of the study and relate this to the costs being evaluated.	
Comparators	7	Describe the interventions or strategies being compared and state why they were chosen.	
Time horizon	8	State the time horizon(s) over which costs and consequences are being evaluated and say why appropriate.	
Discount rate	9	Report the choice of discount rate(s) used for costs and outcomes and say why appropriate.	
Choice of health outcomes	10	Describe what outcomes were used as the measure(s) of benefit in the evaluation and their relevance for the type of analysis performed.	
Measurement of effectiveness	11a	*Single study-based estimates:* Describe fully the design features of the single effectiveness study and why the single study was a sufficient source of clinical effectiveness data.	

For peer review only

	11b	*Synthesis-based estimates:* Describe fully the methods used for identification of included studies and synthesis of clinical effectiveness data.	
Measurement and valuation of preference based outcomes	12	If applicable, describe the population and methods used to elicit preferences for outcomes.	
Estimating resources and costs	13a	*Single study-based economic evaluation:* Describe approaches used to estimate resource use associated with the alternative interventions. Describe primary or secondary research methods for valuing each resource item in terms of its unit cost. Describe any adjustments made to approximate to opportunity costs.	
	13b	*Model-based economic evaluation:* Describe approaches and data sources used to estimate resource use associated with model health states. Describe primary or secondary research methods for valuing each resource item in terms of its unit cost. Describe any adjustments made to approximate to opportunity costs.	
Currency, price date, and conversion	14	Report the dates of the estimated resource quantities and unit costs. Describe methods for adjusting estimated unit costs to the year of reported costs if necessary. Describe methods for converting costs into a common currency base and the exchange rate.	
Choice of model	15	Describe and give reasons for the specific type of decision-analytical model used. Providing a figure to show model structure is strongly recommended.	
Assumptions	16	Describe all structural or other assumptions underpinning the decision-analytical model.	
Analytical methods	17	Describe all analytical methods supporting the evaluation. This could include methods for dealing with skewed, missing, or censored data; extrapolation methods; methods for pooling data; approaches to validate or make adjustments (such as half cycle corrections) to a model; and methods for handling population heterogeneity and uncertainty.	
**Results**			
Study parameters	18	Report the values, ranges, references, and, if used, probability distributions for all parameters. Report reasons or sources for distributions used to represent uncertainty where appropriate. Providing a table to show the input values is strongly recommended.	
Incremental costs and outcomes	19	For each intervention, report mean values for the main categories of estimated costs and outcomes of interest, as well as mean differences between the comparator groups. If applicable, report incremental cost-effectiveness ratios.	
Characterising uncertainty	20a	*Single study-based economic evaluation:* Describe the effects of sampling uncertainty for the estimated incremental cost and incremental effectiveness parameters, together with the impact	

For peer review only

		of methodological assumptions (such as discount rate, study perspective).	
	20b	*Model-based economic evaluation:* Describe the effects on the results of uncertainty for all input parameters, and uncertainty related to the structure of the model and assumptions.	
Characterising heterogeneity	21	If applicable, report differences in costs, outcomes, or cost-effectiveness that can be explained by variations between subgroups of patients with different baseline characteristics or other observed variability in effects that are not reducible by more information.	
**Discussion**			
Study findings, limitations, generalisability, and current knowledge	22	Summarise key study findings and describe how they support the conclusions reached. Discuss limitations and the generalisability of the findings and how the findings fit with current knowledge.	
**Other**			
Source of funding	23	Describe how the study was funded and the role of the funder in the identification, design, conduct, and reporting of the analysis. Describe other non-monetary sources of support.	
Conflicts of interest	24	Describe any potential for conflict of interest of study contributors in accordance with journal policy. In the absence of a journal policy, we recommend authors comply with International Committee of Medical Journal Editors recommendations.	

For consistency, the CHEERS Statement checklist format is based on the format of the CONSORT statement checklist

The **ISPOR CHEERS Task Force Report** provides examples and further discussion of the 24-item CHEERS Checklist and the CHEERS Statement. It may be accessed via the *Value in Health* link or via the ISPOR Health Economic Evaluation Publication Guidelines – CHEERS: Good Reporting Practices webpage: http://www.ispor.org/TaskForces/EconomicPubGuidelines.asp

The citation for the CHEERS Task Force Report is:
Husereau D, Drummond M, Petrou S, et al. Consolidated health economic evaluation reporting standards (CHEERS)—Explanation and elaboration: A report of the ISPOR health economic evaluations publication guidelines good reporting practices task force. Value Health 2013;16:231-50.

For peer review only
